# Socio-Cognitive Determinants of Lifestyle Behavior in the Context of Dementia Risk Reduction: A Population-Based Study in the Netherlands

**DOI:** 10.3233/JAD-231369

**Published:** 2024-05-28

**Authors:** Jeroen Bruinsma, Vasileios S. Loukas, Thomas Kassiotis, Irene Heger, Anna Rosenberg, Leonie N. C. Visser, Francesca Mangialasche, Dimitrios I. Fotiadis, Sten Hanke, Rik Crutzen

**Affiliations:** a Department of Health Promotion of the Care and Public Health Research Institute at Maastricht University, Maastricht, The Netherlands; b Department of Materials Science and Engineering, Unit of Medical Technology and Intelligent Information Systems, University of Ioannina, Ioannina, Greece; c Biomedical Research Institute, Foundation for Research and Technology–Hellas, FORTH-BRI, Ioannina, Greece; d Computational BioMedicine Laboratory, Institute of Computer Science, Foundation for Research and Technology – Hellas, FORTH-ICS-CBML, Heraklion, Greece; e Department of Psychiatry and Neuropsychology of the School for Mental Health and Neuroscience at Maastricht University, Maastricht, The Netherlands; f Finnish Institute for Health and Welfare, Helsinki, Finland; g Department of Neurobiology, Care Sciences and Society, Division of Clinical Geriatrics, Center for Alzheimer Research, Karolinska Institutet, Stockholm, Sweden; h Department of Medical Psychology, Amsterdam University Medical Centre, University of Amsterdam, Amsterdam, The Netherlands; i Amsterdam Public Health Research Institute, Quality of Care/Personalized Medicine, Amsterdam, The Netherlands; j FINGERS Brain Health Institute, Stockholm, Sweden; k Theme Inflammation and Aging, Medical Unit Aging, Karolinska University Hospital, Solna, Sweden; l Institute of eHealth at University of Applied Science at FH Joanneum, Graz, Austria

**Keywords:** Alzheimer’s disease, behavioral medicine, dementia, health promotion, health risk behaviors, prevention, public health

## Abstract

**Background:**

Unhealthy behavior increases the risk of dementia. Various socio-cognitive determinants influence whether individuals persist in or alter these unhealthy behaviors.

**Objective:**

This study identifies relevant determinants of behavior associated to dementia risk.

**Methods:**

4,104 Dutch individuals (40–79 years) completed a screening questionnaire exploring lifestyle behaviors associated with dementia risk. Subsequently, 3,065 respondents who engaged in one or more unhealthy behaviors completed a follow-up questionnaire investigating socio-cognitive determinants of these behaviors. Cross-tables were used to assess the accuracy of participants’ perceptions regarding their behavior compared to recommendations. Confidence Interval-Based Estimation of Relevance (CIBER) was used to identify the most relevant determinants of behavior based on visual inspection and interpretation.

**Results:**

Among the respondents, 91.3% reported at least one, while 65% reported two or more unhealthy lifestyle behaviors associated to dementia risk. Many of them were not aware they did not adhere to lifestyle recommendations. The most relevant determinants identified include attitudes (i.e., lacking a passion for cooking and finding pleasure in drinking alcohol or smoking), misperceptions on social comparisons (i.e., overestimating healthy diet intake and underestimating alcohol intake), and low perceived behavioral control (i.e., regarding changing physical inactivity, altering diet patterns, and smoking cessation).

**Conclusions:**

Individual-level interventions that encourage lifestyle change should focus on enhancing accurate perceptions of behaviors compared to recommendations, while strengthening perceived control towards behavior change. Given the high prevalence of dementia risk factors, combining interventions at both individual and environmental levels are likely to be the most effective strategy to reduce dementia on a population scale.

## INTRODUCTION

A growing body of evidence has identified modifiable risk and protective factors for dementia [1, 2]. These insights pave the way for the development of individual and environmental level preventive interventions to reduce the rising total prevalence of dementia. The current strategy strongly revolves around multidomain approaches to encourage healthy lifestyle changes among individuals at-risk [3], particularly in middle and older age. Across the lifespan, a wide variety of modifiable lifestyle factors have been associated with dementia risk [1], such as physical inactivity, unhealthy diet, psychological stress, smoking, low sleep quality, and limited social and cognitive stimulating activities [4–6]. All these lifestyle factors are partly influenced by behavior specific socio-cognitive determinants that determine if individuals persist in or alter their behavior [7, 8]. Examples of socio-cognitive determinants are attitudes (i.e., a latent disposition or tendency to respond with favorableness or unfavorableness to a behavior), social norms (i.e., the perceived social pressure to perform or not perform a behavior), and perceived behavioral control (i.e., the perceived degree of being capable of, or have control over, performing a behavior) [9].

These socio-cognitive determinants influence behavioral choices, yet environmental factors also play a pivotal role [10, 11]. For instance, such environmental factors include the availability of green spaces for exercise, the affordability of fruits and vegetables, as well as policies that prohibit the sale of alcohol or impose taxes on tobacco products. These environmental factors can impact behavior directly or indirectly by influencing socio-cognitive determinants such as attitudes, norms, and perceived behavioral control [8]. This underscores the complexity of behavior change. While there is extensive literature on determinants of behavior [8], insights into the specific determinants relevant to dementia risk reduction are limited. For example, the Motivation to Change Lifestyle and Health Behaviors for Dementia Risk Reduction Scale (MCLHB-DRR) offers understanding of determinants of generic lifestyle change [12], without delving into determinants of specific behaviors, such as attitudes about diet or self-efficacy towards smoking cessation. This represents a limitation, as individuals are often more positive towards broader lifestyle change than to altering particular unhealthy behaviors [13]. Furthermore, it is well-known that determinants are highly behavior specific, which makes it impossible to intuitively select relevant determinants, even for experts in behavior change [14].

With this study, we aim to obtain a better understanding of relevant socio-cognitive determinants in the context of dementia risk reduction, to inform the selection of appropriate behavior change methods to change these determinants through interventions [7, 8]. The current focus is primarily at the individual level, as this research is part of a larger project that includes a clinical trial on a digitally supported lifestyle program to promote brain health among older adults (LETHE, clinicaltrial.gov identifier NCT05565170) [15, 16]. Nevertheless, the findings may offer valuable insights for new research to explore how environmental factors influence behavior directly and through socio-cognitive determinants.

## METHODS

In this cross-sectional study, we used a screening questionnaire to identify individuals with room to improve their lifestyle behavior and we invited them for a follow-up questionnaire to explore socio-cognitive determinants of these behaviors. The study protocol was pre-registered [17] and materials and data to replicate the findings are open access available [18]. The findings are reported using the Strengthening the Reporting of Observational studies in Epidemiology (STROBE) checklist [19].

### Recruitment

In September 2022, respondents were recruited via the International Organization Standardization (ISO)-certified Internet research agency Flycatcher that has a large panel in the Netherlands. Dutch speaking respondents aged 40 to 79 years were eligible for participation because lifestyle behavior is associated with dementia in mid-life and late-life [20, 21].

A sample size estimation indicated that at least 4,000 respondents were required for the screening questionnaire to ensure sufficient statistical power for analyses using data from the follow-up. This estimation accounted for population prevalence of lifestyle behaviors (i.e., smoking had the lowest population prevalence, ≈20% [22]), the response rate expectancy of Flycatcher (≈75%), and the ability to detect correlations coefficients of 0.2 with a half-width of 0.1 using a confidence interval of 95% [23]).

### Questionnaire development

Questionnaires were developed in triangulation with literature, an interview study (*n* = 23) [13] and think-aloud sessions with experts in dementia risk reduction (*n* = 4). The questionnaires were pre-tested with three middle-aged and older individuals of low socio-economic status (SES) who participate in an advisory board consulting on research. This iterative and collaborative development process refined our approach. For instance, the screening questionnaire included twelve modifiable risk and protective factors from the LIfestyle for BRAin health (LIBRA) index [6, 24] to identify respondents with room for lifestyle improvement and experts assisted with setting inclusion cut-offs based on their experience and research.

The follow-up questionnaire assessed socio-cognitive determinants derived from various theories to achieve a comprehensive understanding of their impact [8, 25]. Definitions and measurement instructions for behavioral determinant research informed the item development [26]. Insights from a prior interview study [13] led to the development of items targeting behavior-specific determinants. For example, interviewees frequently mentioned they did not enjoy cooking, which resulted in an item to assess attitudes towards cooking. Expert think-aloud sessions and a pre-test with low SES individuals supported further refinement of the items in terms of face and content validity.

### The screening questionnaire

The screening questionnaire, based on the LIBRA index, aimed to identify respondents who actively engaged in one or more unhealthy lifestyle behaviors. This included physical inactivity, low adherence to a Mediterranean diet, overconsumption of alcohol, smoking, and low engagement in social and cognitive activities. The 9-item Rapid Assessment of Physical Activity (RAPA) [27] was used to identify respondents who were physically inactive, according to Dutch recommendations of doing at least 150 minutes of moderate-to-vigorous activity per week, spread over several days [28]. The 14-item Mediterranean Diet Adherence Screener (MEDAS14; scores ranging from 0–14) [29] was utilized to select respondents with low adherence to the Mediterranean Diet (i.e., score≤5). According to Dutch diet guidelines, the Mediterranean diet aligns with most recommendations by emphasizing high consumption of vegetables, fruits, whole grain products, nuts, legumes, oils, unsaturated fats, poultry, and fish as well as low intake of red or processed meat, high-fat dairy, hard fats, salt, and sugar-sweetened beverages [30]. Two items from the Alcohol Use Disorders Identification Test (AUDIT) [31, 32] were utilized to identify respondents who consumed more than 1 unit of alcohol per day on average. Although research on dementia risk suggests higher alcohol intake as harmful (i.e., 14 to 21 units per week), the approach used in this study aligns with existing recommendations in the Netherlands to prevent disease in general [30]. Self-constructed items were employed to identify respondents who smoked and to assess the number of cigarettes (or other tobacco products) they consumed daily [33]. Engagement in social and cognitive activities was assessed using a self-constructed list of 12 activities, based on previous questionnaires used in reseaerch on dementia risk reduction [34]. Respondents were classified as socially and cognitively inactive based on a < median-split of weekly activities within our sample [33].

Other health-related modifiable risk factors included in LIBRA were assessed by asking respondents if a doctor had ever informed them about having coronary heart disease, renal dysfunction, diabetes, high cholesterol, hypertension, or depression [35]. Obesity (body mass index, BMI≥30) was determined using self-reported weight and height (i.e., BMI = kg/m^2^).

### The follow-up questionnaire

Respondents who actively engaged in one or more unhealthy lifestyle behaviors were invited to participate in the follow-up questionnaire exploring socio-cognitive determinants of these behaviors. The follow-up questionnaire employed self-constructed items assessing determinants separately for physical activity, Mediterranean diet, alcohol intake, smoking, and engagement in social and cognitive activities. All respondents completed items on physical activity, Mediterranean diet, and social and cognitive activities because, to an extent, everyone engages in these behaviors. Items on alcohol and smoking were only completed by participants who indicated to drink alcohol or smoke.

The follow-up questionnaire utilized tailored items for each lifestyle behavior to investigate potentially relevant determinants. Overall, the following categories of socio-cognitive determinants were assessed for each lifestyle behavior separately: (a) beliefs about engaging in sufficient levels of a particular behavior, (b) intentions to change behavior, (c) attitudes, (d) risk perceptions, (e) social and environmental influences, and (f) perceived behavioral control.

### Data analyses

Distribution and spread were calculated for sample characteristics in SPSS. A summative LIBRA-score was calculated, ranging from –5.9 to +12.7, with higher scores indicating a higher relative risk for dementia [6]. Cross-tables were calculated to assess the extent to which respondents perceived they engaged in sufficient levels of a specific lifestyle behavior, in comparison to Dutch lifestyle recommendations, which served as cut-offs for inviting respondents for the follow-up questionnaire.

Per lifestyle behavior the most relevant determinants were identified using Confidence Interval-Based Estimation of Relevance (CIBER) [14, 36]. CIBER was favored over regression analysis due to potential distortion caused by theoretical overlap among determinants [37]. Utilizing diamond plots, CIBER visualizes univariate distributions of items that measure socio-cognitive determinants and their bivariate association with behavior [14]. These plots allow to identify the relevance of determinants based on visual inspection and interpretation. Specifically, scoring distribution and means provide an indication of the room for improvement at the determinant level [36]. In an intervention context, altering a behavioral determinant is relevant only when there is room for improvement. For instance, enhancing knowledge is meaningful if respondents have limited knowledge. Additionally, plotted zero-order associations between determinants and behavior indicate if it is relevant to alter a determinant [36]. For instance, increasing limited knowledge is only relevant if it is associated with healthier behavior.

The *CIBER* function is part of the R package *behaviorchange* [38]. We used the *binaryCIBER* function to identify relevant socio-cognitive determinants for physical inactivity, given the outcome of RAPA is binary (i.e., physically inactive versus physically active). For assessing determinants of the other lifestyle behaviors, we employed the regular CIBER function, which uses continuous outcome scores. Specifically, separate CIBER plots were calculated using summative outcome scores for Mediterranean diet, weekly units of alcohol intake, daily number of tobacco products, and weekly social and cognitive activities.

### Ethics

The study was approved by the Research Ethics Committee of the Faculty of Health, Medicine and Life Sciences of Maastricht University, the Netherlands (FHML-REC/2022/064). Participants gave digital consent and received a standard compensation through Flycatcher of € 0.60 (≈$0.65) for the screening questionnaire and € 2.00 (≈$2.18) for the follow-up questionnaire.

## RESULTS

The screening questionnaire was sent to 6,228 panel members and was completed by 4,104 respondents (65.9%). Of these respondents, 91.3% (*n* = 3,746) reported at least one unhealthy lifestyle behavior (Table 1), while 65% (*n* = 2,666) reported two or more. The follow-up questionnaire was sent to 3,746 respondents and was completed by 81.6% (*n* = 3,056).

**Table 1:** Respondent characteristics and risk factors (N = 4,104)

*Age in years*, Mean (SD); min to max	59.1 (10.57); 40 to 79
*Gender*, male (%)/female (%)	1,710 (41.7)/2,394 (58.3)
*Migration background*, *N* (%)
First generation	158 (3.8)
Second generation	347 (8.5)
*Highest completed educational level^1^, N* (%)
Low	823 (20.1)
Medium	1,545 (37.6)
High	1,736 (42.3)
*LIBRA-factors*, *N* (%)
Physical inactivity^*^	2,172 (52.9%)
Low adherence to Mediterranean diet^*^	2,237 (54.5%)
Overconsumption of alcohol^*^	1,005 (24.5%)
Smoking^*^	456 (11.1%)
Low social and cognitive activity^*^	2,057 (50.1%)
Coronary heart disease	652 (15.9%)
Renal dysfunction	84 (2.0%)
Diabetes	434 (10.6%)
High blood cholesterol	1,257 (30.6%)
Obesity^**^	918 (22.4%)
Hypertension	1,400 (34.1%)
Depression	734 (17.9%)
*LIBRA-score*, Mean (SD); min to max	0.35 (2.90); –5.9 to+10.6
Percentiles, 25;50;75	–2.6; –0.5; 1.6

Protective factors are inverted and represented as risk factors to enhance interpretability. ^1^Based on educational classification (SOI 21) of Statistics Netherlands ^*^Behaviors used to invite respondents for the follow-up questionnaire. ^**^Calculated with data of 4,099 respondents.

### Perceptions about behavior versus lifestyle recommendations

Cross-tables (Table 2) demonstrate that 39.4% of the respondents, whom we considered to be physically inactive, perceived that they engaged in sufficient levels of physical activity. For Mediterranean diet, this figure was 54.8%; for alcohol consumption, it was 43.2%; and for cognitive and social engagement, it was 51.5%.

**Table 2:** Perceptions about behavior versus lifestyle recommendations

**Do you think you do enough weekly physical activity? (*n*** = **3,065)**		
	*Physically inactive , N (%)*	*Physically active, N (%)*
Yes	701 (39.4%)	985 (76.5%)
Somewhat	680 (38.2%)	256 (19.9%)
No	397 (22.3%)	46 (3.6%)
**Do you think you eat healthy? (*n*** = **3,065)**		
	*Low Mediterranean diet, N (%)*	*Medium-high Mediterranean diet, N (%)*
Yes	1007 (54.8%)	943 (76.8%)
Somewhat	753 (41.0%)	273 (22.2%)
No	77 (4.2%)	12 (1.0%)
**Do you think you overconsume alcohol? (*n*** = 2,410)^*^		
	*Overconsumption of alcohol , N (%)*	*Regular consumption of alcohol, N (%)*
Yes	93 (11.1%)	4 (.2%)
Somewhat	384 (45.7%)	122 (5.5%)
No	363 (43.2%)	1444 (64.9%)
**Do you think that you are socially and actively engaged in life? (*n*** = **3,065)**		
	*Low activities , N (%)*	*Medium-high activities, N (%)*
Yes	423 (51.5%)	1506 (67.1%)
Somewhat	321 (39.1%)	601 (26.8%)
No	77 (9.4%)	137 (6.1%)

Physically inactive is less than 150 min of moderate or 60 min of vigorous activity spread over several days of the week. Low adherence to Mediterranean diet is a summative score≤5 on the MEDAS14. Overconsumption of alcohol is drinking over 1 unit per day on average (these items were only completed by respondents who indicated to consume alcohol). Low activity is based on the lowest 25% of weekly social and cognitive activities.

### Interpretating CIBER-plots to indicate relevant behavioral determinants

**Fig. 1:**
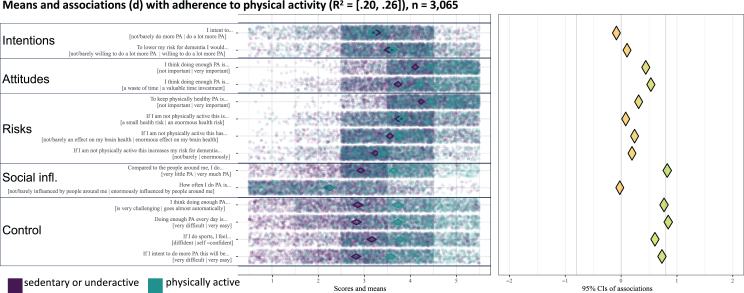
Socio-cognitive determinants of physical activity.

**Fig. 2:**
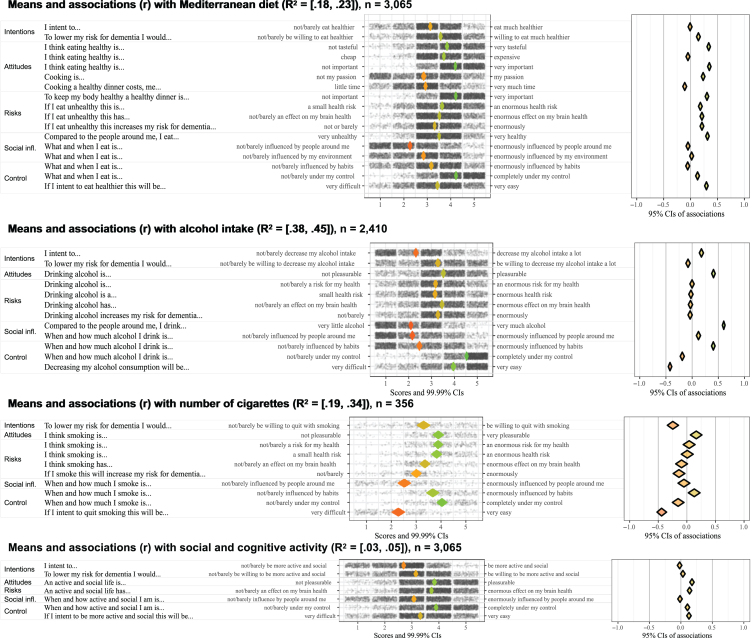
Socio-cognitive determinants of diet, alcohol intake, smoking, and social and cognitive activity.

Figures 1and 2 present the CIBER-plots, visualizing scoring distributions with scatterplots and means, and illustrating associations between determinants and outcome behavior. Tables with the underlying data are available in the Supplementary Material.

### Intentions to change lifestyle behavior

Scoring distributions indicate respondents had moderate intentions to change lifestyle behaviors, with scores averaging around 3 on a 5-point scale (Figs. 1 and 2). Intentions to reduce future dementia risk were slightly higher than general behavior change intentions. Despite this, the weak associations between intentions and outcome behavior suggest restricted relevance.

### Attitudes

Distributions and associations highlight the relevance of passion for cooking and pleasure from alcohol or smoking (Figs. 1 and 2). Scores for cooking passion average below 3 on a 5-point scale, indicating room for improvement, which has a moderate positive association with a healthier diet. Scoring distributions for pleasure derived from alcohol or smoking are above 3.5 on a 5-point scale, suggesting an opportunity to reduce these positive attitudes, especially in light of their moderate to strong associations with higher consumption levels.

### Risk perceptions

Distributions show respondents had realistic risk perceptions about the negative effects of unhealthy behavior on physical health, brain health, and dementia risk (Figs. 1 and 2). Scores concentrate between 3 and 4 on a 5-point scale, indicating limited room for enhancement of these risk perceptions. Restricted relevance is further supported by weak associations with outcome behaviors.

### Social influences

Respondents viewed their diet as relatively healthy compared to others, with scores between 3 and 4 on a 5-point scale (Figs. 1 and 2). This indicates that there is room for improvement, considering that 54.5% of the respondents had a low adherence to the Mediterranean diet. Similarly, there is room to improve accurate perceptions regarding alcohol intake compared to others. Scores ranged mainly between 1 and 3 on a 5-point scale, while 24.5% of the respondents indicated that their alcohol consumption exceeds the recommended level.

### Behavioral control

Across behaviors, perceived control was identified as the most relevant determinant (Figs. 1 and 2). Mean scores among physically inactive respondents fall below 3 on a 5-point scale, suggesting room for improvement, while strong associations indicate that more control is associated with higher activity levels. Although respondents felt in control of their eating behavior, they perceived less control over initiating dietary changes, with scores averaging 3.5 on a 5-point scale. Higher perceived control was moderately correlated with a healthier diet, which indicates relevance. Additionally, perceived control over smoking cessation was low, as scores average around 2 on a 5-point scale. This underscores its relevance, especially since higher perceived control towards cessation strongly correlates with less smoking. In contrast to smoking, respondents perceived high control over alcohol intake, suggesting limited room for enhancement.

### Explained variance

The CIBER-plots had varying explanatory power (95% CI of R^2^). The plot for alcohol intake had the highest explanatory power (R^2^ = 38–45%), followed by smoking (R^2^ = 19–34%), physical activity (R^2^ = 20–26%), and diet (R^2^ = 18–23%). The plot for social and cognitive activities had low explanatory power (R^2^ = 3% to 5%), with no determinants identified as relevant based on scoring distributions and associations.

## DISCUSSION

### Key findings

Most of our respondents had room for improvement in lifestyle behavior but were unaware about this room for improvement. Other relevant socio-cognitive determinants identified are attitudes (i.e., limited passion for cooking and deriving pleasure from alcohol or smoking), misperceptions on social comparisons (i.e., overestimating healthy diet intake and underestimating alcohol intake), and low perceived control over behavior change (i.e., being more physically active, diet improvements, and smoking cessation).

### Interpretation

Misconceptions about adhering to lifestyle recommendations can stem from insufficient awareness about guidelines, combined with overestimation of healthy behaviors and an underestimation of unhealthy ones. The Protection Motivation Theory provides a framework for understanding how these misconceptions about behavior can reduce the motivation to change [39, 40] by influencing threat appraisal (i.e., perceived susceptibility and severity) [41]. For instance, misconceptions about adhering to recommendations may lead to lower perceived susceptibility, which decreases the inclination to act [40]. This may explain why our respondents had limited intentions towards initiating change, which aligns with other research showing only 32% of the physically inactive individuals, 18% of those not adhering to diet recommendations, and 29% of high alcohol consumers have intentions to change [42].

Our findings further highlight the relevance of perceived behavioral control, a concept akin to self-efficacy [41, 43]. Individuals who feel more in control over their behavior, particularly in overcoming barriers [43], are more inclined to initiate behavior change [44]. However, many of our respondents perceived to have limited control over initiating behavior change, potentially diminishing their intentions. Notably, respondents who drank alcohol perceived high levels of control while underestimating their consumption compared to others. These findings are consistent with earlier indicators that drinkers are often overconfident about their ability to control alcohol intake [45], while they overestimate others’ and underestimate their own consumption levels [46].

Based on the findings, motivating individuals to reduce dementia risk through lifestyle changes should prioritize enhancing accurate perceptions about behavior while strengthening behavioral control. Individual-level interventions could use self-monitoring methods like measuring step count or completing a food diary to decrease misconceptions about the adherence to recommendations. Self-monitoring may already be a steppingstone towards positive lifestyle change, through the mere measurement effect [47, 48]. Providing timely, individualized, non-punitive, and actionable feedback may further encourage healthy lifestyle improvements by making individuals aware of their own behavior and by providing specific cues to action [49, 50]. Goal setting and action planning methods are also well-known methods to enhance perceived behavioral control, preferably by gradually increasing the achievability of certain behavioral changes [30]. Formulating coping responses to overcome specific barriers and cope with difficult situations may further facilitate goal achievement [51]. In earlier qualitative research [13], we observed that low perceived control frequently resulted from earlier failed attempts to change lifestyle behavior. This underscores the importance of providing support to individuals who experience relapse, for example by encouraging them to view it as a part of the behavior change process and use it as an opportunity to learn from the experience [52]. Based on these findings, we strongly advocate for the adoption of specific, evidence-based behavior change methods to support individuals with changing their lifestyles to reduce dementia risk. This requires careful reconsideration of intervention ellements to prioritize methods that are able to target relevant socio-cognitive determinants to increase the likelihood of behavior change. For instance, by emphasizing enjoyable cooking activities, drawing inspiration from successful programs [53] to make participants enhousiastic about creating new eating habits.

While individual-level interventions are important, they must be supplemented by environmental-level strategies to reduce dementia prevalence [54]. Although our sample is only partially representative of the Dutch public, many of our participants had room to improve one or multiple lifestyle behaviors to reduce their future risk of dementia. We observed slightly lower prevalence rates for unhealthy lifestyle behaviors compared to the broader Dutch population [55], indicating that our results might understate the potential for dementia risk reduction in the Netherlands. Given the high prevalence of both unhealthy behavior and dementia, as well as the substantial impact of the environment on behavior, the effectiveness of individual-level interventions alone is questionable [54]. Therefore, we advocate for a multi-faceted approach that combines individual and environmental interventions to facilitate healthy lifestyle behaviors and reduce the risk of dementia at the population level.

### Practical applications

The research findings are currently used to shape the development of individual-level behavior change applications that are integrated in the digital LETHE intervention to prevent cognitive decline and dementia [15, 16]. LETHE utilizes a smartphone app and Fitbit smartwatch to monitor lifestyle behavior. One of the features under development is a system to classify participants into weekly adherence pathways (i.e., green, yellow, red). This system would enable lifestyle monitoring to offer personalized digital and in person feedback and support. Furthermore, smartphone functionalities are under development to empower participants to actively monitor a range of lifestyle behaviors. For instance, through a weekly performance score with motivational feedback messages. These examples show how the research findings can guide the development of individual-level applications to support behavior change.

### Strengths and limitations

This cross-sectional study employed several measures to enhance credibility, including pre-registration [17] of the protocol to ensure transparency and minimize reporting bias. All materials and data are openly accessible, supporting reproducibility. A key strength of this study is the recruitment through the research agency Flycatcher. Unlike health-related panels, Flycatcher surveys a wide range of topics, including marketing and policy research. This may have helped us to reach a broad audience that is not only interested in participating in health-related research. However, this approach might have introduced selection bias because individuals who volunteer for panels may not accurately reflect the entire population. For instance, individuals with limited digital skills might find it more challanging to participate in an Internet-based panel. Although 80% of the Dutch public has basic digital skills [56] this indicates a substantial part might be excluded from our research.

The study utilized a robust screening process with the validated LIBRA-index, but we relied on unvalidated items to assess social and cognitive activities, which may have affected measurement accuracy that limited the exploratory power. Additionally, future research is needed to explore the determinants of social and cognitive activities more thoroughly and to investigate the impact of environmental factors and demographic differences, particularly socio-economic status, on lifestyle behavior in the context of dementia risk.

### Conclusions

Promoting lifestyle changes to reduce dementia risk is complex. Individual-level interventions that encourage lifestyle change should prioritize enhancing accurate perceptions of lifestyle behaviors in comparison to recommendations, while strengthening perceived control over initiating behavioral changes.

## AUTHOR CONTRIBUTIONS

Jeroen Bruinsma (Conceptualization; Data curation; Formal analysis; Investigation; Methodology; Visualization; Writing – original draft; Writing – review & editing); Vasileios S. Loukas (Conceptualization; Investigation; Writing – original draft; Writing – review & editing); Thomas Kassiotis (Conceptualization; Methodology); Irene Heger (Conceptualization; Methodology; Writing – review & editing); Anna Rosenberg (Writing – review & editing); Leonie N.C. Visser (Writing – review & editing); Francesca Mangialasche (Writing – review & editing); Dimitrios I. Fotiadis (Funding acquisition; Writing – review & editing); Sten Hanke (Conceptualization; Funding acquisition; Writing – review & editing); Rik Crutzen (Conceptualization; Formal analysis; Funding acquisition; Investigation; Methodology; Supervision; Writing – original draft; Writing – review & editing).

## Supplementary Material

Supplementary Material Screening questionnaire

Supplementary Material Screening questionnaire (English translation)

Supplementary Material Follow-up questionnaire

Supplementary Material Follow-up questionnaire (English translation)

Supplementary Material Flycatcher Research Report follow-up questionnaire

Supplementary Material Flycatcher Research Report screening questionnaire

Supplementary Material 1. Section 3.1 Sample characteristics

Supplementary Material 2. Section 3.1 Table 1

Supplementary Material 3. Section 3.2 Table 2

Supplementary Material 4. Data CIBER plots (Fig 1 and Fig 2)

## Data Availability

The materials and data supporting the conclusions are available on the Open Science Framework through https://osf.io/eqbgr/.
