# Supplementary material for: Socio-Cognitive Determinants of Lifestyle Behavior in the Context of Dementia Risk Reduction: A Population-Based Study in the Netherlands
Source: J Alzheimers Dis. 2024 May 28;99(3):941–52. doi: 10.3233/JAD-231369 (PMC11191482; doi:10.3233/JAD-231369)
Supplement: Supplementary Material — Screening questionnaire [file jad-99-jad231369-s001.docx]

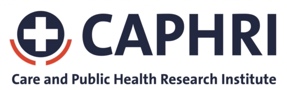


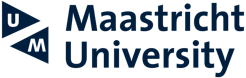
**Eerste Vragenlijst
Onderzoek: Leefstijl, hersengezondheid en dementie**

Bedankt voor uw interesse in ons wetenschappelijk onderzoek over leefstijl, (hersen)gezondheid en dementie. Welkom bij de **eerste vragenlijst**. Dit is een screeningsvragenlijst om te beoordelen of u in aanmerking komt voor het vervolgonderzoek middels een **tweede vragenlijst**.

Het invullen van deze vragenlijst duurt ongeveer 10 tot 15 minuten. Na het volledig afronden van de vragenlijst ontvangt u [XX] punten.

Als u in aanmerking komt voor het vervolgonderzoek dan ontvangt u binnen 21 dagen een uitnodiging tot deelname aan de **tweede vragenlijst**. De tweede vragenlijst bevat specifieke vragen op basis van uw antwoorden op deze vragenlijst. Het invullen van de tweede vragenlijst duurt ongeveer 15 tot 20 minuten.

We willen u vragen om de vragenlijst volledig en eerlijk in te vullen. U kunt geen goede of foute antwoorden geven.

Bij vragen kunt u contact opnemen met dr. Jeroen Bruinsma via [jeroen.bruinsma@maastrichtuniversity.nl](mailto:jeroen.bruinsma@maastrichtuniversity.nl)

[**start vragenlijst 1**] [**aantal items 51**]

Vanwege kwaliteitsdoeleinden stellen we u de volgende vraag.

Deze vragenlijs is bedoeld voor [naam]. Bent u deze persoon?

- ja
- nee

In het eerste deel van de vragenlijst stellen we u een aantal vragen over uw lengte en gewicht, maar ook over uw algemene gezondheid. Vervolgens stellen we u vragen over uw levensstijl bijvoorbeeld over uw voedings- en bewegingspatroon.

Maak een inschatting als u het niet zeker weet.

| Wat is uw lengte? | … cm |
| --- | --- |
| Wat is uw gewicht? | … Kg |
|  | |
| Heeft uw arts u ooit verteld dat u een hoog cholesterol heeft? | Ja Nee Weet ik niet |
| Heeft uw arts u ooit verteld dat u een blijvende (chronische) nierziekte heeft? | Ja Nee Weet ik niet |
| Heeft uw arts u ooit verteld dat u een hoge bloeddruk heeft? | Ja Nee Weet ik niet |
| Heeft uw arts u ooit verteld dat u suikerziekte (diabetes) heeft? | Ja Nee Weet ik niet |
| Heeft uw arts u ooit verteld dat u een aandoening aan hart- en/of bloedvaten heeft? | Ja Nee Weet ik niet |
| Heeft uw arts u ooit verteld dat u een depressie heeft of depressief bent? | Ja Nee Weet ik niet |
| Heeft u een beperking die het lastig maakt om lichamelijk te bewegen? | Ja  Nee |
| Kunt u toelichten waardoor u beperkt wordt tijdens het bewegen?  Conditie: lichamelijke beperking = ja | [vrije tekst] |

De volgende vragen gaan over roken en het drinken van alcohol.

| Rookt u? | Ja, ik rook  Ik heb gerookt, maar ben gestopt  Nee, ik rook niet |
| --- | --- |
| Hoeveel sigaretten (of andere tabak producten) rookt u ongeveer per dag?  Conditie: deelnemer rookt | … per dag |
| Hoeveel jaar geleden bent u gestopt met roken?  Conditie: deelnemer is gestopt met roken | … jaren geleden |
| Hoe vaak drinkt u alcoholhoudende drank?  *Bijvoorbeeld wijn, bier of whisky.* | Nooit  maandelijks of minder  2 tot 4 keer per maand  2 tot 3 keer per week  4 of meer keer per week  Dagelijks |
| Hoeveel glazen alcohol drinkt u gemiddeld als u drinkt?  Conditie: deelnemer alcoholhoudende drank | 1 of 2 glazen per keer 3 of 4 glazen per keer 5 of 6 glazen per keer 7 of 9 glazen per keer 10 of meer glazen per keer |

(conditie: at random krijgt 50% ‘bewegen 1’ de andere 50% ‘bewegen 2’)

De volgende vragen gaan over de hoeveelheid en intensiteit van uw lichamelijke beweging. Tijdens lichamelijke beweging verhoogt uw hartslag. U kunt bijvoorbeeld bewegen voor uw plezier, werk, het huishouden of tijdens het dagelijkse vervoer.

De intensiteit van lichamelijke beweging wordt bepaald aan de hand van de hoeveelheid energie die het u kost. Hieronder ziet u een aantal voorbeelden van lichamelijke beweging op verschillende intensiteit niveaus. De afbeeldingen dienen als hulpmiddel bij het beantwoorden van de volgende vragen.

| Ik doe zelden of nooit aan lichamelijke beweging. | Ja  Nee |
| --- | --- |
| **Vragen over licht intensieve activiteit** |  |
| Ik doe iedere week aan licht intensieve lichamelijke beweging. | Ja Nee |
| Ik doe aan licht of matig intensieve lichamelijke beweging maar niet iedere week. | Ja Nee |
| **Vragen over matig intensieve beweging** |  |
| Ik doe iedere week aan matig intensieve lichamelijke beweging maar minder dan 30 minuten per dag of minder dan vijf dagen per week. | Ja Nee |
| Ik doe per dag 30 minuten of meer aan matig intensieve lichamelijke beweging. Dit doe ik op 5 of meer dagen per week. | Ja Nee |
| **Vragen over zwaar intensieve beweging** |  |
| Ik doe iedere week aan zwaar intensieve lichamelijke beweging maar minder dan 20 minuten per dag of minder dan drie dagen per week. | Ja Nee |
| Ik doe per dag 20 minuten of meer aan zwaar intensieve lichamelijke beweging. Dit doe ik op 3 of meer dagen per week. | Ja Nee |
| **Vragen over krachttraining en rekoefeningen** |  |
| Ik doe één of meerdere keren per week oefeningen om sterker te worden zoals krachttraining, trainen met gewichten of fitness. | Ja Nee |
| Ik doe één of meerdere keren per week oefeningen om leniger te worden zoals rekoefeningen of yoga. | Ja Nee |

(conditie: at random krijgt 50% ‘bewegen 1’ de andere 50% ‘bewegen 2’)

De volgende vragen gaan over de hoeveelheid en intensiteit van uw lichamelijke beweging. Tijdens lichamelijke beweging verhoogt uw hartslag. U kunt bijvoorbeeld bewegen voor uw plezier, werk, het huishouden of tijdens het dagelijkse vervoer.

De intensiteit van lichamelijke beweging wordt bepaald aan de hand van de hoeveelheid energie die het u kost. Hieronder ziet u een aantal voorbeelden van lichamelijke beweging op verschillende intensiteit niveaus. De afbeeldingen dienen als hulpmiddel bij het beantwoorden van de volgende vragen.

| Ik doe zelden of nooit aan lichamelijke beweging. | Ja Nee |
| --- | --- |
| Ik doe aan licht of matig intensieve lichamelijke beweging maar niet iedere week. | Ja Nee |
| Ik doe iedere week aan licht intensieve lichamelijke beweging. | Ja Nee |
| Ik doe iedere week aan matig intensieve lichamelijke beweging maar minder dan 30 minuten per dag of minder dan vijf dagen per week. | Ja Nee |
| Ik doe iedere week aan zwaar intensieve lichamelijke beweging maar minder dan 20 minuten per dag of minder dan drie dagen per week. | Ja Nee |
| Ik doe per dag 30 minuten of meer aan matig intensieve lichamelijke beweging. Dit doe ik op 5 of meer dagen per week. | Ja Nee |
| Ik doe per dag 20 minuten of meer aan zwaar intensieve lichamelijke beweging. Dit doe ik op 3 of meer dagen per week. | Ja Nee |
| Ik doe één of meerdere keren per week oefeningen om sterker te worden zoals krachttraining, trainen met gewichten of fitness. | Ja Nee |
| Ik doe één of meerdere keren per week oefeningen om leniger te worden zoals rekoefeningen of yoga. | Ja Nee |

De volgende vragen gaan over uw voedingspatroon. Gezonde voeding is rijk aan vitamines, mineralen en andere bouwstoffen. Bij gezonde voeding kunt u denken aan verse groente, fruit en volkoren producten. Daarnaast is ook het eten van vis, olijfolie, noten en peulvruchten gezond.

| Gebruikt u vooral olijfolie in plaats van boter bij het bereiden van voedsel? | Ja  Nee  Geen van beide |
| --- | --- |
| Hoeveel eetlepels olijfolie gebruikt u gemiddeld per dag?  *Dit gaat om olijfolie die u gebruikt bij het koken of bijvoorbeeld in dressing van een salade.* | Geen of minder dan 1 eetlepel  1 eetlepel 2-3 eetlepels 4 of meer eetlepels |
| Hoeveel porties groente eet u gemiddeld per dag?  *Bijvoorbeeld gekookte of rauwe groente. Aardappelen tellen niet mee.*  *Eén portie is 150 gram (ongeveer een half bord).* | Geen of minder dan 1 portie 1 portie 2 porties 3 of meer porties |
| Hoeveel porties vers fruit eet u gemiddeld per dag?  *Bijvoorbeeld één sinaasappel, één banaan of één hand vol met bessen. Eén glas vers fruitsap telt ook als één portie.* | Geen of minder dan 1 portie 1 portie 2 porties 3 of meer porties |
| Eet u vlees? | Ja  Nee |
| Hoeveel porties rood vlees of bewerkt vlees eet u gemiddeld per week?  *Bijvoorbeeld rund-, varkens-, lams- of kalfsvlees.*  *Voorbeelden van bewerkt vlees zijn hamburgers, worst en boterhamworst.*  *Eén portie is 100-150 gram.* | 0-1 portie 2-4 porties 5-6 porties 7 meer porties |
| Eet u vaker kip, kalkoen, konijnenvlees, vis of een vegetarische vleesvervanger dan rood vlees of bewerkt vlees?  *Voorbeelden van rood vlees zijn rund-, varkens-, lams- of kalfsvlees.*  *Voorbeelden van bewerkt vlees zijn hamburgers, worst en boterhamworst.* | Ja  Nee |
| Hoeveel porties boter, margarine of room eet u gemiddeld per dag?  *Eén portie is één dessertlepel voor botermargarine of twee eetlepels voor room.* | Geen of minder dan 1 portie 1 portie Meer dan 1 portie |

| Hoeveel glazen frisdrank drinkt u gemiddeld per dag?  *Dit gaat bijvoorbeeld over aanlenglimonade, cola, dubbelfris, energydrank, en fruitsap dat niet vers is.* | Geen of minder dan 1 glas Meer dan 1 glas |
| --- | --- |
| Hoeveel glazen wijn drinkt u gemiddeld per week? | 0-1 glas 2-6 glazen 7-14 glazen Meer dan 14 glazen |
| Hoeveel porties peulvruchten eet u gemiddeld per week?  *Bijvoorbeeld bonen, (kikker)erwten of linzen.*  *Eén portie is 150 gram (ongeveer een half bord)* | Geen of minder dan 1 portie 1 portie 2 porties 3 of meer porties |
| Hoeveel porties vis of schaaldieren eet u gemiddeld per week?  *Bijvoorbeeld witvis, zalm, kibbeling, garnalen of mosselen.*  *Eén portie is 100-150 gram (ongeveer een kwart tot half bord).* | Geen of minder dan 1 portie 1 portie 2 porties 3 of meer porties |
| Hoe vaak per week eet u een niet zelfgemaakt toetje, snoep, gebak of koek?  *Bijvoorbeeld koekjes, taart, vlaai, yoghurt, vla, cake of ijs.* | Nooit of minder dan 1 keer 1 keer 2 keer 3 keer 4 of meerdere keren |
| Hoeveel porties ongezouten noten eet u per week?  *Bijvoorbeeld ongezouten pinda’s, amandelen, hazelnoten, kastanjes, walnoten of paranoten. Eén portie is 30 gram (ongeveer één hand vol).* | Geen of minder dan 1 portie 1 portie 2 porties 3 of meer porties |
| Hoe vaak per week eet u een maaltijd met olijfolie, verse tomatensaus, ui of knoflook?  *Bijvoorbeeld verse pastasaus, uiensoep of een salade met tomaat.* | Nooit of minder dan 1 keer 1 keer 2 of meerdere keren |

De volgende vragen hebben betrekking op hoe actief en sociaal u in het leven staat.

| Hoe vaak spreekt u af met anderen?  *Bijvoorbeeld met vrienden, kennissen of familieleden.* | Dagelijks 4-6 keer per week 2-3 keer per week 1 keer per week 2-3 keer per maand Een paar keer per jaar Nooit |
| --- | --- |
| Hoe vaak leest u?  *Bijvoorbeeld de krant, boeken of tijdschriften.* | Dagelijks 4-6 keer per week 2-3 keer per week 1 keer per week 2-3 keer per maand Een paar keer per jaar Nooit |
| Hoe vaak puzzelt u?  *Bijvoorbeeld kruiswoordpuzzels, legpuzzels of Sudoku.* | Dagelijks 4-6 keer per week 2-3 keer per week 1 keer per week 2-3 keer per maand Een paar keer per jaar Nooit |
| Hoe vaak schrijft u?  *Bijvoorbeeld verhalen, kaarten, brieven of artikelen.* | Dagelijks 4-6 keer per week 2-3 keer per week 1 keer per week 2-3 keer per maand Een paar keer per jaar Nooit |
| Hoe vaak speelt u spelletjes?  *Bijvoorbeeld bordspellen, spelcomputer, online spelletjes of kaarten.* | Dagelijks 4-6 keer per week 2-3 keer per week 1 keer per week 2-3 keer per maand Een paar keer per jaar Nooit |

| Hoe vaak maakt u muziek?  *Bijvoorbeeld door te zingen of een instrument te bespelen.* | Dagelijks 4-6 keer per week 2-3 keer per week 1 keer per week 2-3 keer per maand Een paar keer per jaar Nooit |
| --- | --- |
| Hoe vaak bent u actief binnen het verenigingsleven?  *Bijvoorbeeld bij de voetbalclub, kerkgenootschap of kaartclub.* | Dagelijks 4-6 keer per week 2-3 keer per week 1 keer per week 2-3 keer per maand Een paar keer per jaar Nooit |
| Hoe vaak besteed u tijd aan het leren van iets nieuws?  *Bijvoorbeeld een nieuwe taal, muziek spelen of een nieuw recept.* | Dagelijks 4-6 keer per week 2-3 keer per week 1 keer per week 2-3 keer per maand Een paar keer per jaar Nooit |
| Hoe vaak doet u aan handwerken?  *Bijvoorbeeld knutselen, kaarten maken of houtbewerking.* | Dagelijks 4-6 keer per week 2-3 keer per week 1 keer per week 2-3 keer per maand Een paar keer per jaar Nooit |
| Hoe vaak tuiniert u?  *Bijvoorbeeld het gras maaien of onkruid wieden.* | Dagelijks 4-6 keer per week 2-3 keer per week 1 keer per week 2-3 keer per maand Een paar keer per jaar Nooit  Niet van toepassing |

| Hoe vaak past u op?  *Bijvoorbeeld op de kleinkinderen of een huisdier.* | Dagelijks 4-6 keer per week 2-3 keer per week 1 keer per week 2-3 keer per maand Een paar keer per jaar Nooit  Niet van toepassing |
| --- | --- |
| Hoe vaak doet u iets voor een ander?  *Bijvoorbeeld de boodschappen, mantelzorg of vrijwilligerswerk.* | Dagelijks 4-6 keer per week 2-3 keer per week 1 keer per week 2-3 keer per maand Een paar keer per jaar Nooit |
| Hoe vaak gebruikt u digitale technologie?  *Bijvoorbeeld een mobiele telefoon, computer of de iPad.* | Dagelijks 4-6 keer per week 2-3 keer per week 1 keer per week 2-3 keer per maand Een paar keer per jaar Nooit |
| Welke digitale technologie gebruikt u?  *Meerdere antwoorden mogelijk.* | Mobiele telefoon  Apps zoals Whatsapp of Google Maps  IPad of tablet  Computer of laptop  Smartwatch  Spelcomputer  Overige: … |
| Overige: | Vrije tekst |
| Voelt u zich zelfverzekerd als u digitale technologie gebruikt?  *Bijvoorbeeld een mobiele telefoon, computer of iPad.* | Helemaal niet zelfverzekerd  1  2  3  4  5  Heel erg zelfverzekerd |

| Digitale hulpmiddelen kunnen me … helpen om mijn levensstijl te verbeteren.  *Voorbeelden van digitale hulpmiddelen zijn Apps of een mobiele telefoon, computer of iPad.* | niet of nauwelijks  1  2  3  4  5  erg veel |
| --- | --- |
| Kunt u toelichten waarom digitale hulpmiddelen u niet of weinig kunnen helpen om gezonder te leven?  Conditie = vorige item 1-2 | Vrije tekst |
| Kunt u toelichten waarom digitale hulpmiddelen u kunnen helpen om gezonder te leven?  Conditie = vorige item 3-5 | Vrije tekst |

Geef van onderstaande stellingen aan of ze helemaal niet of helemaal wel op u van toepassing zijn.

| Ik houd meer van ingewikkelde dan van eenvoudige vraagstukken. | Helemaal niet  Grotendeels niet  Deels wel en deels niet  Grotendeels wel  Helemaal wel |
| --- | --- |
| Ik ben graag verantwoordelijk voor situaties waarbij ik veel moet nadenken. | Helemaal niet  Grotendeels niet  Deels wel en deels niet  Grotendeels wel  Helemaal wel |
| Nadenken vind ik plezierig. | Helemaal niet  Grotendeels niet  Deels wel en deels niet  Grotendeels wel  Helemaal wel |
| Ik doe liever iets waarbij ik weinig hoef na te denken dan iets waarbij ik veel moet nadenken. | Helemaal niet  Grotendeels niet  Deels wel en deels niet  Grotendeels wel  Helemaal wel |
| Ik heb plezier in taken waarbij ik nieuwe oplossingen moet bedenken. | Helemaal niet  Grotendeels niet  Deels wel en deels niet  Grotendeels wel  Helemaal wel |

| Ik heb een voorkeur voor moeilijke en belangrijke taken, in plaats van makkelijke en minder belangrijke taken. | Helemaal niet  Grotendeels niet  Deels wel en deels niet  Grotendeels wel  Helemaal wel |
| --- | --- |

Bedankt voor het invullen van de vragenlijst! Als u nog vragen heeft over uw levensstijl of (hersen)gezondheid dan kunt u dit met uw huisarts bespreekbaar maken.

Als u in aanmerking komt voor het vervolgonderzoek ontvangt u van ons een uitnodiging van deelname aan het tweede onderzoek binnen 21 dagen.

Graag willen wij u nu nog enkele vragen stellen over de vragenlijst die u zojuist heeft ingevuld. Uw mening kan ons helpen toekomstige vragenlijsten verder te verbeteren. Als u deze vraag wilt overslaan, klikt u gewoon op Volgende om door te gaan naar het einde van de vragenlijst.

**Wat vond u van de vragenlijst?**

| interessant onderwerp | o | o | o | o | o | oninteressant onderwerp |
| --- | --- | --- | --- | --- | --- | --- |
| te kort | o | o | o | o | o | te lang |
| duidelijke vragen | o | o | o | o | o | onduidelijke vragen |
| prettig om in te vullen | o | o | o | o | o | onprettig om in te vullen |

**Indien u nog opmerkingen heeft over het onderwerp van deze vragenlijst, kunt u daarvoor de ruimte hieronder gebruiken.**

|  |
| --- |

Controleer uw gegevens. Indien de gegevens niet meer correct zijn, wordt u na het versturen van de vragenlijst automatisch doorgeleid naar een pagina waar u deze kunt aanpassen.

[link naar persoonlijke paspoort]

Heel erg bedankt voor uw medewerking! Klik op Volgende om uw antwoorden te versturen.

Klik op ‘volgende’ om deze vragenlijst af te sluiten.
