# Supplementary material for: Socio-Cognitive Determinants of Lifestyle Behavior in the Context of Dementia Risk Reduction: A Population-Based Study in the Netherlands
Source: J Alzheimers Dis. 2024 May 28;99(3):941–52. doi: 10.3233/JAD-231369 (PMC11191482; doi:10.3233/JAD-231369)
Supplement: Supplementary Material — Screening questionnaire (English translation) [file jad-99-jad231369-s002.docx]

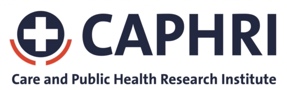


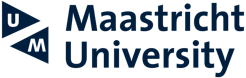
**First questionnaire
Study: Lifestyle, Brain Health, and Dementia**

Thank you for your interest in our scientific research on lifestyle, brain health, and dementia. Welcome to the **first questionnaire**. This is a screening survey to assess whether you qualify for the follow-up study via a **second questionnaire**.

Completing this questionnaire will take approximately 10 to 15 minutes. Upon completing the questionnaire in its entirety, you will receive [XX] points

If you qualify for the follow-up study, you will receive an invitation to participate in the second questionnaire within 21 days. The second questionnaire contains specific questions based on your answers to this questionnaire. Completing the second questionnaire will take about 15 to 20 minutes

We kindly ask you to fill out the questionnaire completely and honestly. There are no right or wrong answers.

If you have any questions, please contact Dr. Jeroen Bruinsma at [jeroen.bruinsma@maastrichtuniversity.nl](mailto:jeroen.bruinsma@maastrichtuniversity.nl)

[**start questionnaire 1**] [**number of items 51**]

For quality purposes, we have the following question for you.

This questionnaire is intended for [name]. Are you this person?

- yes
- no

In the first part of the questionnaire, we'll ask you several questions about your height and weight, as well as your general health. Subsequently, we'll ask questions about your lifestyle, such as your dietary and exercise habits.

Make an estimate if you do not know for sure.

| What is your height? | … cm |
| --- | --- |
| What is your weight? | … Kg |
|  | |
| Has your doctor ever told you that you have high cholesterol? | Yes  No  I don’t know |
| Has your doctor ever told you that you have a chronic kidney disease? | Yes  No  I don’t know |
| Has your doctor ever told you that you have high blood pressure? | Yes  No  I don’t know |
| Has your doctor ever told you that you have diabetes? | Yes  No  I don’t know |
| Has your doctor ever told you that you have a heart and/or vascular condition? | Yes  No  I don’t know |
| Has your doctor ever told you that you have depression? | Yes  No  I don’t know |
| Do you have any physical limitations that make it difficult for you to be physically active? | Yes  No |
| Could you explain what limits your movement? Condition: physical limitation = yes | [free text] |

The following questions are about smoking and alcohol consumption.

| Do you smoke? | Yes, I smoke  I used to smoke but I have quit  No, I do not smoke |
| --- | --- |
| Approximately how many cigarettes (or other tobacco products) do you smoke per day?  Condition: respondent smokes | … per day |
| How many years ago did you quit smoking?  Condition: respondent has quit smoking | … years ago |
| How often do you consume alcoholic beverages?  *For example, wine, beer, or whisky.* | Never  Monthly or less  2 to 4 times per month  2 to 3 times per week  4 or more times per week  Daily |
| On average, how many glasses of alcohol do you drink when you consume alcohol?  Condition: respondent drinks alcohol | 1 or 2 glasses at a time  3 or 4 glasses at a time  5 or 6 glasses at a time  7 or 9 glasses at a time  10 or more glasses at a time |

(Condition: at random 50% ‘physical activity 1’ and the other 50% ‘physical activity 2’)

The following questions concern the amount and intensity of your physical activity. During physical activity, your heart rate increases. This can include activities for pleasure, work, household chores, or during daily transportation.

The intensity of physical activity is determined by the amount of energy it requires. Below, you'll find examples of physical activities at different intensity levels. The images serve as a guide to answer the following questions.

**
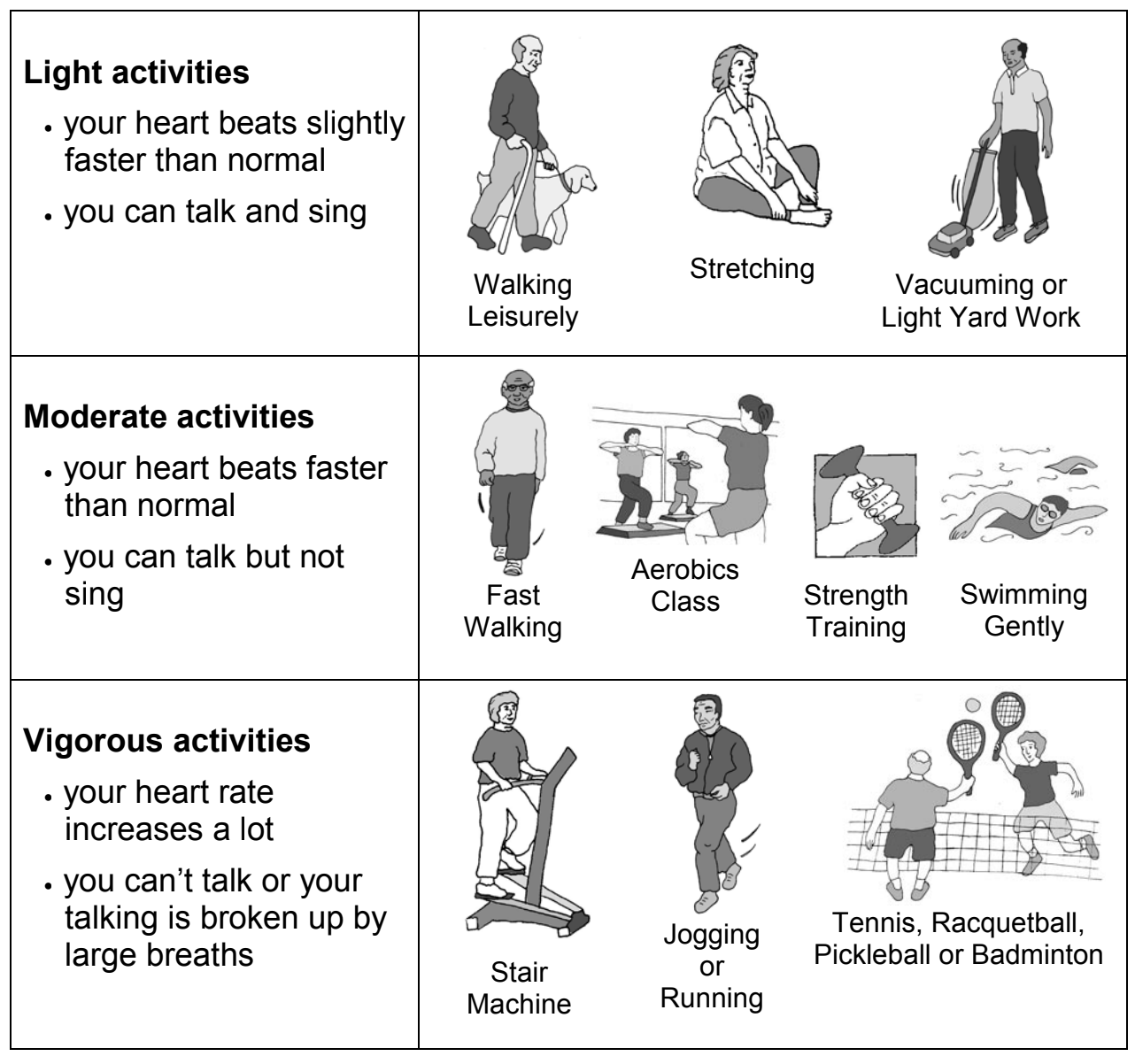
**

| I rarely or never do any physical activities. | Yes  No |
| --- | --- |
| Items on light physical activity |  |
| I do some **light** physical activity every week. | Yes  No |
| I do some **light** or **moderate** physical activities, but not every week. | Yes  No |
| Items on moderate physical activity |  |
| I do **moderate** physical activities every week, but less than 30 minutes a day or 5 days a week. | Yes  No |
| I do 30 minutes or more a day of **moderate** physical activities, 5 or more days a week. | Yes  No |
| Items on vigorous physical activity |  |
| I do **vigorous** physical activities every week, but less than 20 minutes a day or 3 days a week. | Yes  No |
| I do 20 minutes or more a day of **vigorous** physical activities, 3 or more days a week. | Yes  No |
| Items on strength and flexibility |  |
| I do activities to increase muscle **strength**, such as lifting weights or calisthenics, once a week or more. | Yes  No |
| I do activities to improve **flexibility**, such as stretching or yoga, once a week or more. | Yes  No |

(Condition: at random 50% ‘physical activity 1’ and the other 50% ‘physical activity 2’)

The following questions concern the amount and intensity of your physical activity. During physical activity, your heart rate increases. This can include activities for pleasure, work, household chores, or during daily transportation.

The intensity of physical activity is determined by the amount of energy it requires. Below, you'll find examples of physical activities at different intensity levels. The images serve as a guide to answer the following questions.

**
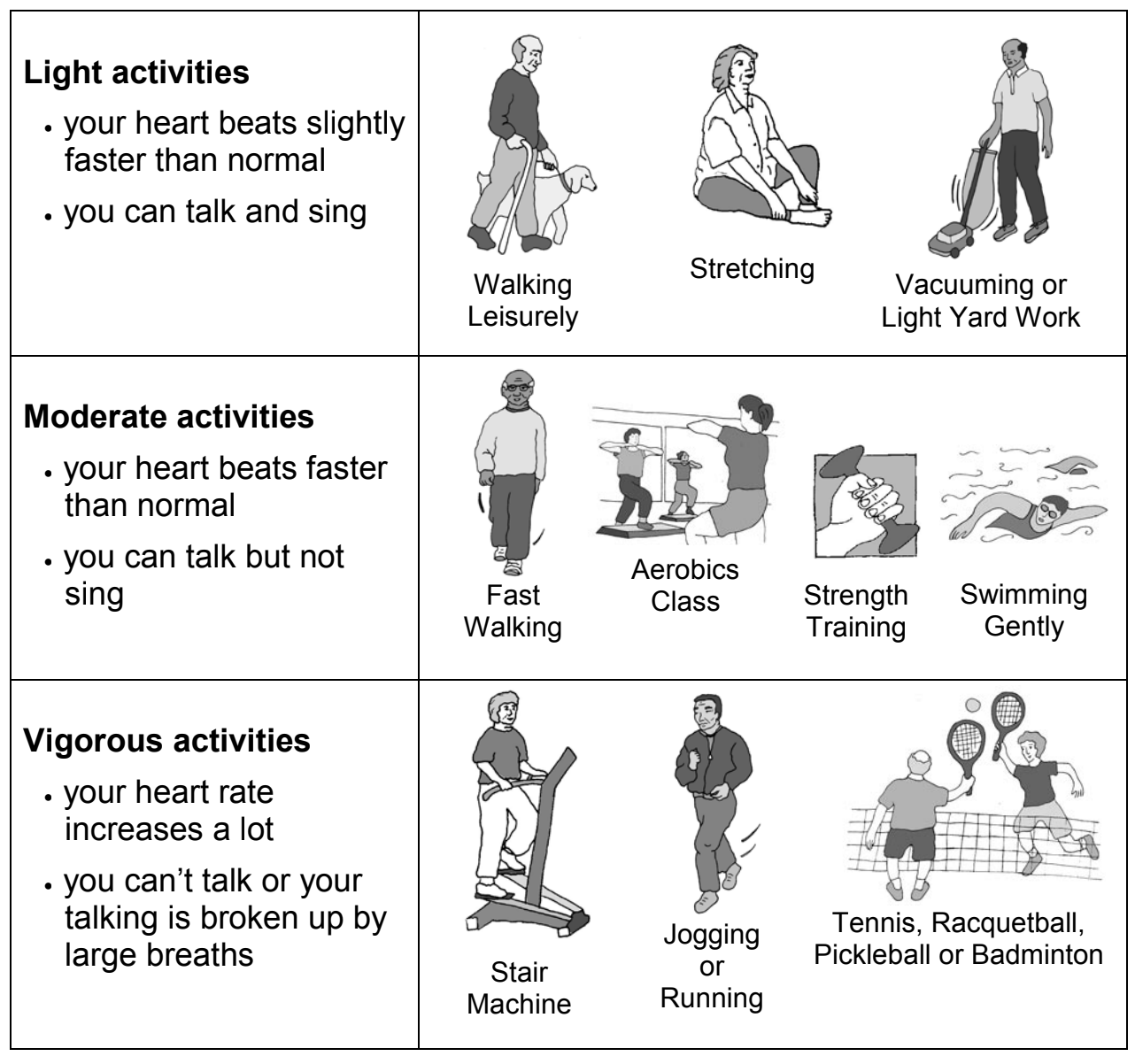
**

| I rarely or never do any physical activities. | Yes  No |
| --- | --- |
| I do some **light** or **moderate** physical activities, but not every week. | Yes  No |
| I do some **light** physical activity every week. | Yes  No |
| I do **moderate** physical activities every week, but less than 30 minutes a day or 5 days a week. | Yes  No |
| I do **vigorous** physical activities every week, but less than 20 minutes a day or 3 days a week. | Yes  No |
| I do 30 minutes or more a day of **moderate** physical activities, 5 or more days a week. | Yes  No |
| I do 20 minutes or more a day of **vigorous** physical activities, 3 or more days a week. | Yes  No |
| I do activities to increase muscle **strength**, such as lifting weights or calisthenics, once a week or more. | Yes  No |
| do activities to improve **flexibility**, such as stretching or yoga, once a week or more. | Yes  No |

The following questions pertain to your dietary pattern. Healthy nutrition is rich in vitamins, minerals, and other essential nutrients. When considering healthy eating, it includes fresh vegetables, fruits, and whole grain products. Additionally, consuming fish, olive oil, nuts, and legumes are also considered healthy.

| Do you mainly use olive oil instead of butter when preparing food? | Yes  No  Neither |
| --- | --- |
| How many tablespoons of olive oil do you use on average per day?  *This includes olive oil used in cooking or, for example, in salad dressing.* | None or less than 1 tablespoon  1 tablespoon  2-3 tablespoons  4 or more tablespoons |
| Hoe How many servings of vegetables do you consume on average per day?  *For example, cooked or raw vegetables. Potatoes are not included. One serving is 150 grams (approximately half a plate).* | None or less than 1 serving  1 serving  2 servings  3 or more servings |
| How many servings of fresh fruit do you consume on average per day?  *For example, one orange, one banana, or one handful of berries. One glass of fresh fruit juice also counts as one serving.* | None or less than 1 serving  1 serving  2 servings  3 or more servings |
| Do you eat meat? | Yes  No |
| How many servings of red meat or processed meat do you consume on average per week?  *For example, beef, pork, lamb, or veal. Examples of processed meat include hamburgers, sausages, and luncheon meats.* *One serving is 100-150 grams.* | 0-1 serving  2-4 servings  5-6 servings  7 or more servings |
| Do you consume chicken, turkey, rabbit meat, fish, or a vegetarian meat substitute more often than red meat or processed meat?  *Examples of red meat include beef, pork, lamb, or veal.*  *Examples of processed meat include hamburgers, sausages, and luncheon meats.* | Yes  No |
| How many servings of butter, margarine, or cream do you consume on average per day? *One serving is one dessert spoon for butter or margarine, or two tablespoons for cream.* | None or less than 1 serving  1 serving  More than 1 serving |

| How many glasses of soda do you consume on average per day?  *This includes drinks like soda, cola, fruit-flavored drinks, energy drinks, and non-fresh fruit juice.* | None or less than 1 glass  1 glass  More than 1 glass |
| --- | --- |
| How many glasses of wine do you consume on average per week? | 0-1 glass  2-6 glasses  7-14 glasses  More than 14 glasses |
| How many servings of legumes do you consume on average per week?  *For example, beans, chickpeas, or lentils. One serving is 150 grams (approximately half a plate).* | None or less than 1 serving  1 serving  2 servings  3 or more servings |
| How many servings of fish or shellfish do you consume on average per week?  *For example, white fish, salmon, fried fish, shrimp, or mussels. One serving is 100-150 grams (approximately a quarter to half a plate).* | None or less than 1 serving  1 serving  2 servings  3 or more servings |
| How often per week do you consume a non-homemade dessert, candy, pastry, or cookies?  *For example, cookies, cake, pudding, yogurt, custard, cake, or ice cream.* | Never or less than 1 time  1 time  2 times  3 times  4 or more times |
| How many servings of unsalted nuts do you consume per week?  *For example, unsalted peanuts, almonds, hazelnuts, chestnuts, walnuts, or Brazil nuts. One serving is 30 grams (approximately one handful).* | None or less than 1 serving  1 serving  2 servings  3 or more servings |
| How often per week do you have a meal containing olive oil, fresh tomato sauce, onion, or garlic?  *For example, fresh pasta sauce, onion soup, or a salad with tomatoes.* | Never or less than 1 time  1 time  2 or more times |

The following questions relate to how active and social you are in life.

| How often do you meet with others?  *For example, with friends, acquaintances, or family members.* | Daily  4-6 times per week  2-3 times per week  Once a week  2-3 times per month  A few times per year  Never |
| --- | --- |
| How often do you read?  *For example, newspapers, books, or magazines.* | Daily  4-6 times per week  2-3 times per week  Once a week  2-3 times per month  A few times per year  Never |
| How often do you do puzzles?  *For example, crosswords, jigsaw puzzles, or Sudoku.* | Daily  4-6 times per week  2-3 times per week  Once a week  2-3 times per month  A few times per year  Never |
| How often do you write?  *For example, stories, cards, letters, or articles.* | Daily  4-6 times per week  2-3 times per week  Once a week  2-3 times per month  A few times per year  Never |
| How often do you play games?  *For example, board games, video games, online games, or cards.* | Daily  4-6 times per week  2-3 times per week  Once a week  2-3 times per month  A few times per year  Never |

| How often do you make music?  *For instance, singing or playing a musical instrument.* | Daily  4-6 times per week  2-3 times per week  Once a week  2-3 times per month  A few times per year  Never |
| --- | --- |
| How often are you active in community organizations?  *For example, at a soccer club, church, or card club.* | Daily  4-6 times per week  2-3 times per week  Once a week  2-3 times per month  A few times per year  Never |
| How often do you spend time learning something new?  *For example, a new language, playing music, or trying a new recipe.* | Daily  4-6 times per week  2-3 times per week  Once a week  2-3 times per month  A few times per year  Never |
| How often do you do handicrafts?  *For example, crafting, card-making, or woodworking.* | Daily  4-6 times per week  2-3 times per week  Once a week  2-3 times per month  A few times per year  Never |
| How often do you garden?  *For example, mowing the lawn or weeding.* | Daily  4-6 times per week  2-3 times per week  Once a week  2-3 times per month  A few times per year  Never |

| How often do you babysit?  *For example, looking after grandchildren or a pet.* | Daily  4-6 times per week  2-3 times per week  Once a week  2-3 times per month  A few times per year  Never  Not applicable |
| --- | --- |
| How often do you do something for others?  *For example, grocery shopping, caregiving, or volunteering.* | Daily  4-6 times per week  2-3 times per week  Once a week  2-3 times per month  A few times per year  Never |
| How often do you use digital technology?  *For example, a mobile phone, computer, or iPad.* | Daily  4-6 times per week  2-3 times per week  Once a week  2-3 times per month  A few times per year  Never |
| Which digital technologies do you use?  *Multiple answers possible.* | Mobile phone  Apps like WhatsApp or Google Maps  iPad or tablet  Computer or laptop  Smartwatch  Game console  Other |
| Other | Free text |
| Do you feel confident using digital technology?  *For example, a mobile phone, computer, or iPad.* | Not at all confident  1  2  3  4  5  Very confident |

| Digitals tools can help me... improve my lifestyle.  Examples of digital tools are apps, a mobile phone, computer, or iPad. | Not or barely  1  2  3  4  5  Very much |
| --- | --- |
| Could you explain why digital tools cannot or do not significantly assist you in improving your health?  Condition = previous item 1-2 | Free text |
| Could you explain why digital tools can assist you in improving your health?  Condition = previous item 3-5 | Free text |

Indicate for the following statements whether they do not apply to you at all or apply completely to you.

| I prefer complex issues over simple ones. | Not at all  Mostly not  Partly yes and partly no  Mostly yes  Completely yes |
| --- | --- |
| I enjoy being responsible for situations that require a lot of thinking. | Not at all  Mostly not  Partly yes and partly no  Mostly yes  Completely yes |
| Thinking is enjoyable for me. | Not at all  Mostly not  Partly yes and partly no  Mostly yes  Completely yes |
| I prefer doing something that requires little thinking over something that requires a lot of thinking. | Not at all  Mostly not  Partly yes and partly no  Mostly yes  Completely yes |
| I find pleasure in tasks that involve devising new solutions | Not at all  Mostly not  Partly yes and partly no  Mostly yes  Completely yes |

| I prefer challenging and significant tasks over easy and less important tasks. | Not at all  Mostly not  Partly yes and partly no  Mostly yes  Completely yes |
| --- | --- |

Thank you for completing the questionnaire! If you have any further questions regarding your lifestyle or (brain) health, you can discuss this with your general practitioner.

If you qualify for the follow-up study, you will receive an invitation from us to participate in the second study within 21 days.

We would like to ask you a few more questions about the questionnaire you have just completed. Your feedback can help us further improve future questionnaires. If you wish to skip this question, simply click 'Next' to proceed to the end of the questionnaire.

What did you think of the questionnaire?

| interesting topic | o | o | o | o | o | uninteresting topic |
| --- | --- | --- | --- | --- | --- | --- |
| too short | o | o | o | o | o | too long |
| clear questions | o | o | o | o | o | unclear questions |
| pleasant to fill out | o | o | o | o | o | unpleasant to fill out |

**If you have any additional comments regarding the topic of this questionnaire, please use the space below.**

|  |
| --- |

Please review your details. If the information is no longer correct, after submitting the questionnaire, you will be automatically redirected to a page where you can make changes.

[link to personal profile]

Thank you very much for your cooperation! Click 'Next' to submit your answers.

Click 'next' to finish this questionnaire.
