# Supplementary material for: Socio-Cognitive Determinants of Lifestyle Behavior in the Context of Dementia Risk Reduction: A Population-Based Study in the Netherlands
Source: J Alzheimers Dis. 2024 May 28;99(3):941–52. doi: 10.3233/JAD-231369 (PMC11191482; doi:10.3233/JAD-231369)
Supplement: Supplementary Material — Follow-up questionnaire [file jad-99-jad231369-s003.docx]

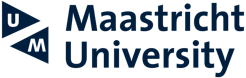

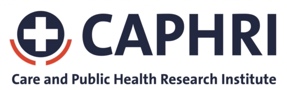
**Tweede Vragenlijst
Onderzoek: Leefstijl en hersengezondheid**

Bedankt voor het invullen van de eerste vragenlijst. Uit uw antwoorden blijkt dat er ruimte voor verbetering is in uw leefstijl om uw hersengezondheid te verbeteren. Door gezonder te gaan leven verkleint u de kans op dementie zo veel als mogelijk.

Om uw hersenen gezond te houden is het belangrijk om voldoende te bewegen, gezond te eten, sociaal en actief in het leven te staan, niet te roken en niet of met mate alcohol te drinken. In deze tweede vragenlijst stellen wij u hierover een aantal vragen. Het invullen van deze vragenlijst duurt ongeveer 15 tot 25 minuten. Na het volledig invullen van deze vragenlijst ontvangt u nog eens [XX] punten.

We willen u wederom vragen om alle vragen eerlijk in te vullen. Wij zijn benieuwd naar uw mening en ervaring. Er zijn geen goede of foute antwoorden.

Bij vragen over het onderzoek kunt u contact opnemen met [jeroen.bruinsma@maastrichtuniversity.nl](mailto:jeroen.bruinsma@maastrichtuniversity.nl).

Als u na het invullen van deze vragenlijst nog vragen heeft over uw levensstijl of (hersen)gezondheid dan kunt u dit met uw huisarts bespreken.

[**start vragenlijst**]

Vanwege kwaliteitsdoeleinden stellen we u de volgende vraag.

Bent u deze persoon?

- ja
- nee

De eerste vragen gaan over uw algemene gezondheid en levensstijl.

| [awareness_79n2w1bj](https://psycore.one/construct/?ucid=awareness_79n2w1bj) | Vindt u dat u een gezonde levensstijl heeft? | Ja Een beetje  Nee  Weet ik niet |
| --- | --- | --- |
|  | Kunt u toelichten waarom u dit vindt? |  |
|  |  | |
| U kunt met één cijfer tussen de 1 en 5 aangeven wat het beste past bij uw ervaring | | |
| [attitude_73dnt5zc](https://psycore.one/construct/?ucid=attitude_73dnt5zc) | Een gezonde levensstijl is voor mij … belangrijk | niet of nauwelijks  1  2  3  4  5  heel erg |
| [attitude_73dnt5zc](https://psycore.one/construct/?ucid=attitude_73dnt5zc)  [risk_perception_79n2fh4t](https://psycore.one/construct/?ucid=risk_perception_79n2fh4t) | Ik vind het veranderen van mijn huidige levensstijl… | overbodig  1  2  3  4  5  noodzakelijk |
| [capacity_73dnt602](https://psycore.one/construct/?ucid=capacity_73dnt602)  [perceivedBehavioralControl_73dnt603](https://psycore.one/construct/?ucid=perceivedBehavioralControl_73dnt603)  [self_control_79n2fh4t](https://psycore.one/construct/?ucid=self_control_79n2fh4t) | Als ik mijn levensstijl wil veranderen dan… | lukt me dat erg moeilijk  1  2  3  4  5  lukt me dat erg makkelijk |
| [risk_perception_79n2fh4t](https://psycore.one/construct/?ucid=risk_perception_79n2fh4t)  [instrAttitude_expectation_73dnt5z6](https://psycore.one/construct/?ucid=instrAttitude_expectation_73dnt5z6) | Mijn risico om ziek te worden … | heeft weinig te maken met mijn levensstijl  1  2  3  4  5  heeft veel te maken met mijn levensstijl |

| [risk_perception_79n2fh4t](https://psycore.one/construct/?ucid=risk_perception_79n2fh4t)  [instrAttitude_expectation_73dnt5z6](https://psycore.one/construct/?ucid=instrAttitude_expectation_73dnt5z6) | Het risico op het krijgen van dementie … | heeft weinig te maken met mijn levensstijl  1  2  3  4  5  heeft veel te maken met mijn levensstijl |
| --- | --- | --- |
| [intention_73dnt604](https://psycore.one/construct/?ucid=intention_73dnt604)  [motivation_79n2fh4q](https://psycore.one/construct/?ucid=motivation_79n2fh4q) | Om het risico op dementie te verminderen ben ik… | niet bereid om mijn levensstijl te veranderen  1  2  3  4  5  bereid om mijn levensstijl te veranderen |

Conditie (screeningsvragenlijst): Roken = ja

De volgende vragen gaan over roken.

| [intention_73dnt604](https://psycore.one/construct/?ucid=intention_73dnt604)  [motivation_79n2fh4q](https://psycore.one/construct/?ucid=motivation_79n2fh4q)  [action_plan_79n2w1bh](https://psycore.one/construct/?ucid=action_plan_79n2w1bh) | Heeft u plannen om minder te gaan roken? | Nee  Ja, ik wil minder gaan roken  Ja, ik wil stoppen met roken |
| --- | --- | --- |
| U kunt met een cijfer tussen de 1 en 5 aangeven wat het beste past bij uw ervaring | | |
| [intention_73dnt604](https://psycore.one/construct/?ucid=intention_73dnt604)  [motivation_79n2fh4q](https://psycore.one/construct/?ucid=motivation_79n2fh4q) | Om het risico op dementie te verminderen ben ik… | niet bereid om te stoppen met roken  1  2  3  4  5  bereid om te stoppen met roken |
| [attitude_73dnt5zc](https://psycore.one/construct/?ucid=attitude_73dnt5zc) | Roken vind ik… | onplezierig  1  2  3  4  5  plezierig |
| [awareness_79n2w1bj](https://psycore.one/construct/?ucid=awareness_79n2w1bj) | Roken vind ik… | niet schadelijk  1  2  3  4  5  erg schadelijk |
| [risk_perception_79n2fh4t](https://psycore.one/construct/?ucid=risk_perception_79n2fh4t) | Roken vind ik… | een erg klein gezondheidsrisico  1  2  3  4  5  een erg groot gezondheidsrisico |
| [risk_perception_79n2fh4t](https://psycore.one/construct/?ucid=risk_perception_79n2fh4t) | Roken heeft… | weinig effect op de gezondheid van mijn hersenen  1  2  3  4  5  veel effect op de gezondheid van mijn hersenen |

| [risk_perception_79n2fh4t](https://psycore.one/construct/?ucid=risk_perception_79n2fh4t)  [instrAttitude_expectation_73dnt5z6](https://psycore.one/construct/?ucid=instrAttitude_expectation_73dnt5z6) | Roken verhoogt mijn risico op dementie… | niet tot nauwelijks  1  2  3  4  5  erg veel |
| --- | --- | --- |
| [inflEnvironment_79n2r0sy](https://psycore.one/construct/?ucid=inflEnvironment_79n2r0sy)  [perceivedBehavioralControl_73dnt603](https://psycore.one/construct/?ucid=perceivedBehavioralControl_73dnt603)  [self_control_79n2fh4t](https://psycore.one/construct/?ucid=self_control_79n2fh4t) | Wanneer en hoeveel ik rook… | wordt niet of nauwelijks beïnvloed door mensen om mij heen  1  2  3  4  5  wordt sterk beïnvloed door mensen om mij heen |
| [habit_79n2r0sz](https://psycore.one/construct/?ucid=habit_79n2r0sz)  [perceivedBehavioralControl_73dnt603](https://psycore.one/construct/?ucid=perceivedBehavioralControl_73dnt603)  [self_control_79n2fh4t](https://psycore.one/construct/?ucid=self_control_79n2fh4t) | Wanneer en hoeveel ik rook… | niet of nauwelijks beïnvloed door gewoonten  1  2  3  4  5  sterk beïnvloed door gewoonten |
| [perceivedBehavioralControl_73dnt603](https://psycore.one/construct/?ucid=perceivedBehavioralControl_73dnt603)  [self_control_79n2fh4t](https://psycore.one/construct/?ucid=self_control_79n2fh4t) | Wanneer en hoeveel ik rook heb ik… | niet of nauwelijks zelf in de hand  1  2  3  4  5  helemaal zelf in de hand |
| [capacity_73dnt602](https://psycore.one/construct/?ucid=capacity_73dnt602) | Als ik me voorneem om te stoppen met roken dan… | lukt dat erg moeilijk  1  2  3  4  5  lukt dat erg makkelijk |
| [goal_79n2r0sz](https://psycore.one/construct/?ucid=goal_79n2r0sz) | Om te stoppen met roken heb ik een duidelijk doel nodig | Helemaal mee oneens  1  2  3  4  5  Helemaal mee eens |

| [action_plan_79n2w1bh](https://psycore.one/construct/?ucid=action_plan_79n2w1bh) | Om te stoppen met roken heb ik een goed plan nodig | Helemaal mee oneens  1  2  3  4  5  Helemaal mee eens |
| --- | --- | --- |
| [need_79n2fh4r](https://psycore.one/construct/?ucid=need_79n2fh4r) | Om te stoppen met roken heb ik veel persoonlijke begeleiding nodig | Helemaal mee oneens  1  2  3  4  5  Helemaal mee eens |
| [need_79n2fh4r](https://psycore.one/construct/?ucid=need_79n2fh4r) | Om te stoppen met roken heb ik goed advies nodig | Helemaal mee oneens  1  2  3  4  5  Helemaal mee eens |
| [attitude_73dnt5zc](https://psycore.one/construct/?ucid=attitude_73dnt5zc) | Digitale hulpmiddelen (bijvoorbeeld een smartwatch of een app) kunnen me … helpen om te stoppen met roken | niet of nauwelijks  1  2  3  4  5  erg veel |

De volgende vragen gaan over lichamelijke beweging. U kunt op verschillende manieren bewegen bijvoorbeeld door te wandelen, zwemmen of te fietsen.

| [awareness_79n2w1bj](https://psycore.one/construct/?ucid=awareness_79n2w1bj) | Vindt u dat u wekelijks voldoende beweegt? | Ja Een beetje  Nee |
| --- | --- | --- |
| U kunt met een cijfer tussen de 1 en 5 aangeven wat het beste past bij uw ervaring. | | |
| [intention_73dnt604](https://psycore.one/construct/?ucid=intention_73dnt604)  [motivation_79n2fh4q](https://psycore.one/construct/?ucid=motivation_79n2fh4q)  [action_plan_79n2w1bh](https://psycore.one/construct/?ucid=action_plan_79n2w1bh) | Ik ben van plan om … meer te bewegen | niet of nauwelijks  1  2  3  4  5  veel |
| [intention_73dnt604](https://psycore.one/construct/?ucid=intention_73dnt604)  [motivation_79n2fh4q](https://psycore.one/construct/?ucid=motivation_79n2fh4q) | Om het risico op dementie te verminderen ben ik… | niet bereid om veel meer te gaan bewegen  1  2  3  4  5  bereid om veel meer te gaan bewegen |
| [attitude_73dnt5zc](https://psycore.one/construct/?ucid=attitude_73dnt5zc) | Voldoende beweging vind ik… | overbodig  1  2  3  4  5  noodzakelijk |
| [attitude_73dnt5zc](https://psycore.one/construct/?ucid=attitude_73dnt5zc) | Voldoende beweging vind ik… | zonde van mijn tijd  1  2  3  4  5  een waardevolle dagbesteding |
| [attitude_73dnt5zc](https://psycore.one/construct/?ucid=attitude_73dnt5zc)  [habit_79n2r0sz](https://psycore.one/construct/?ucid=habit_79n2r0sz) | Voldoende bewegen lukt me… | vrijwel automatisch  1  2  3  4  5  erg moeilijk |

| [perceivedBehavioralControl_73dnt603](https://psycore.one/construct/?ucid=perceivedBehavioralControl_73dnt603)  [attitude_73dnt5zc](https://psycore.one/construct/?ucid=attitude_73dnt5zc) | Dagelijks voldoende bewegen vind ik… | erg lastig  1  2  3  4  5  erg makkelijk |
| --- | --- | --- |
| [attitude_73dnt5zc](https://psycore.one/construct/?ucid=attitude_73dnt5zc)  [instrAttitude_expectation_73dnt5z6](https://psycore.one/construct/?ucid=instrAttitude_expectation_73dnt5z6) | Om mijn lichaam gezond te houden is beweging… | niet zo belangrijk  1  2  3  4  5  heel erg belangrijk |
| [risk_perception_79n2fh4t](https://psycore.one/construct/?ucid=risk_perception_79n2fh4t) | Als ik te weinig beweeg dan is dit een… | erg klein gezondheidsrisico  1  2  3  4  5  erg groot gezondheidsrisico |
| [risk_perception_79n2fh4t](https://psycore.one/construct/?ucid=risk_perception_79n2fh4t) | Als ik te weinig beweeg dan heeft dit… | erg weinig effect op de gezondheid van mijn hersenen  1  2  3  4  5  erg veel effect op de gezondheid van mijn hersenen |
| [risk_perception_79n2fh4t](https://psycore.one/construct/?ucid=risk_perception_79n2fh4t)  [instrAttitude_expectation_73dnt5z6](https://psycore.one/construct/?ucid=instrAttitude_expectation_73dnt5z6) | Als ik te weinig beweeg dan verhoogt dit mijn risico op dementie… | heel erg weinig  1  2  3  4  5  heel erg veel |
| [referentBehavior_73dnt5zk](https://psycore.one/construct/?ucid=referentBehavior_73dnt5zk)  [perceivedNorms_73dnt5zq](https://psycore.one/construct/?ucid=perceivedNorms_73dnt5zq) | Vergeleken met de mensen om mij heen beweeg ik … | erg weinig  1  2  3  4  5  erg veel |

| [inflEnvironment_79n2r0sy](https://psycore.one/construct/?ucid=inflEnvironment_79n2r0sy)  [perceivedBehavioralControl_73dnt603](https://psycore.one/construct/?ucid=perceivedBehavioralControl_73dnt603)  [self_control_79n2fh4t](https://psycore.one/construct/?ucid=self_control_79n2fh4t) | Hoeveel ik beweeg wordt… | niet of nauwelijks beïnvloed door mensen om mij heen  1  2  3  4  5  sterk beïnvloed door mensen om mij heen |
| --- | --- | --- |
| [attitude_73dnt5zc](https://psycore.one/construct/?ucid=attitude_73dnt5zc) | Bewegen doe ik het liefst… | alleen  met iemand samen  in groepsverband |
| [attitude_73dnt5zc](https://psycore.one/construct/?ucid=attitude_73dnt5zc) | Als ik sport voel ik me… | onzeker  1  2  3  4  5  zelfverzekerd |
| [goal_79n2r0sz](https://psycore.one/construct/?ucid=goal_79n2r0sz) | Om voldoende te bewegen heb ik een duidelijk doel nodig | Helemaal mee oneens  1  2  3  4  5  Helemaal mee eens |
| [action_plan_79n2w1bh](https://psycore.one/construct/?ucid=action_plan_79n2w1bh) | Om voldoende te bewegen heb ik goed plan nodig | Helemaal mee oneens  1  2  3  4  5  Helemaal mee eens |
| [capacity_73dnt602](https://psycore.one/construct/?ucid=capacity_73dnt602) | Als ik me voorneem om meer te bewegen dan… | lukt dat erg moeilijk  1  2  3  4  5  lukt dat erg makkelijk |

| [need_79n2fh4r](https://psycore.one/construct/?ucid=need_79n2fh4r) | Om meer te bewegen heb ik veel persoonlijke begeleiding nodig | Helemaal mee oneens  1  2  3  4  5  Helemaal mee eens |
| --- | --- | --- |
| [need_79n2fh4r](https://psycore.one/construct/?ucid=need_79n2fh4r) | Om meer te bewegen heb ik goed advies nodig | Helemaal mee oneens  1  2  3  4  5  Helemaal mee eens |
| [attitude_73dnt5zc](https://psycore.one/construct/?ucid=attitude_73dnt5zc) | Digitale hulpmiddelen (bijvoorbeeld een smartwatch of een app) kunnen me … helpen om meer te bewegen | niet of nauwelijks  1  2  3  4  5  erg veel |

De volgende vragen gaan over gezonde voeding. Gezonde voeding is rijk aan vitamines, mineralen en andere bouwstoffen. Bij gezonde voeding kunt u denken aan verse groente, fruit en volkoren producten. Daarnaast is ook het eten van (vette) vis, olijfolie, noten en peulvruchten gezond.

| [awareness_79n2w1bj](https://psycore.one/construct/?ucid=awareness_79n2w1bj) | Vindt u dat u gezond eet? | | Ja Een beetje  Nee |
| --- | --- | --- | --- |
| U kunt met een cijfer tussen de 1 en 5 aangeven wat het beste past bij uw ervaring | | | |
| [intention_73dnt604](https://psycore.one/construct/?ucid=intention_73dnt604)  [motivation_79n2fh4q](https://psycore.one/construct/?ucid=motivation_79n2fh4q)  [action_plan_79n2w1bh](https://psycore.one/construct/?ucid=action_plan_79n2w1bh) | Ik ben van plan om … gezonder te eten | | niet of nauwelijks  1  2  3  4  5  veel |
| [intention_73dnt604](https://psycore.one/construct/?ucid=intention_73dnt604)  [motivation_79n2fh4q](https://psycore.one/construct/?ucid=motivation_79n2fh4q) | Om het risico op dementie te verminderen ben ik… | | niet bereid om veel gezonder te gaan eten  1  2  3  4  5  bereid om veel gezonder te gaan eten |
| [attitude_73dnt5zc](https://psycore.one/construct/?ucid=attitude_73dnt5zc) | Gezond eten vind ik… | | niet zo lekker  1  2  3  4  5  erg lekker |
| [attitude_73dnt5zc](https://psycore.one/construct/?ucid=attitude_73dnt5zc) | Gezond eten vind ik… | | goedkoop  1  2  3  4  5  duur |
| [attitude_73dnt5zc](https://psycore.one/construct/?ucid=attitude_73dnt5zc) | Gezond eten vind ik… | | niet zo belangrijk  1  2  3  4  5  heel erg belangrijk |
| [attitude_73dnt5zc](https://psycore.one/construct/?ucid=attitude_73dnt5zc) | | Koken is voor mij… | geen passie  1  2  3  4  5  een passie |
| [attitude_73dnt5zc](https://psycore.one/construct/?ucid=attitude_73dnt5zc) | | Gezond koken kost mij… | erg weinig tijd  1  2  3  4  5  erg veel tijd |
| [attitude_73dnt5zc](https://psycore.one/construct/?ucid=attitude_73dnt5zc)  [instrAttitude_expectation_73dnt5z6](https://psycore.one/construct/?ucid=instrAttitude_expectation_73dnt5z6) | | Om mijn lichaam gezond te houden is gezond eten… | niet zo belangrijk  1  2  3  4  5  heel erg belangrijk |
| [risk_perception_79n2fh4t](https://psycore.one/construct/?ucid=risk_perception_79n2fh4t) | | Als ik ongezond eet dan is dit een… | erg klein gezondheidsrisico  1  2  3  4  5  erg groot gezondheidsrisico |
| [risk_perception_79n2fh4t](https://psycore.one/construct/?ucid=risk_perception_79n2fh4t) | | Als ik ongezond eet dan heeft dit… | erg weinig effect op de gezondheid van mijn hersenen  1  2  3  4  5  erg veel effect op de gezondheid van mijn hersenen |
| [risk_perception_79n2fh4t](https://psycore.one/construct/?ucid=risk_perception_79n2fh4t)  [instrAttitude_expectation_73dnt5z6](https://psycore.one/construct/?ucid=instrAttitude_expectation_73dnt5z6) | | Als ik ongezond eet dan verhoogt dit mijn risico op dementie… | heel erg weinig  1  2  3  4  5  heel erg veel |

| [referentBehavior_73dnt5zk](https://psycore.one/construct/?ucid=referentBehavior_73dnt5zk)  [perceivedNorms_73dnt5zq](https://psycore.one/construct/?ucid=perceivedNorms_73dnt5zq) | Vergeleken met de mensen om mij heen eet ik… | heel erg ongezond  1  2  3  4  5  heel erg gezond |
| --- | --- | --- |
| [inflEnvironment_79n2r0sy](https://psycore.one/construct/?ucid=inflEnvironment_79n2r0sy)  [perceivedBehavioralControl_73dnt603](https://psycore.one/construct/?ucid=perceivedBehavioralControl_73dnt603)  [self_control_79n2fh4t](https://psycore.one/construct/?ucid=self_control_79n2fh4t) | Wat ik eet wordt… | niet of nauwelijks beïnvloed door mensen om mij heen  1  2  3  4  5  sterk beïnvloed door mensen om mij heen |
| [habit_79n2r0sz](https://psycore.one/construct/?ucid=habit_79n2r0sz)  [perceivedBehavioralControl_73dnt603](https://psycore.one/construct/?ucid=perceivedBehavioralControl_73dnt603)  [self_control_79n2fh4t](https://psycore.one/construct/?ucid=self_control_79n2fh4t) | Wat ik eet wordt… | niet of nauwelijks beïnvloed door gewoonten  1  2  3  4  5  sterk beïnvloed door gewoonten |
| [inflEnvironment_79n2r0sy](https://psycore.one/construct/?ucid=inflEnvironment_79n2r0sy)  [perceivedBehavioralControl_73dnt603](https://psycore.one/construct/?ucid=perceivedBehavioralControl_73dnt603)  [self_control_79n2fh4t](https://psycore.one/construct/?ucid=self_control_79n2fh4t) | Wat ik eet wordt… | niet of nauwelijks beïnvloed door de plaats waar ik ben  1  2  3  4  5  sterk beïnvloed door de plaats waar ik ben |
| [perceivedBehavioralControl_73dnt603](https://psycore.one/construct/?ucid=perceivedBehavioralControl_73dnt603)  [self_control_79n2fh4t](https://psycore.one/construct/?ucid=self_control_79n2fh4t) | Wat ik eet heb ik… | niet of nauwelijks zelf in de hand  1  2  3  4  5  volledig zelf in de hand |
| [goal_79n2r0sz](https://psycore.one/construct/?ucid=goal_79n2r0sz) | Om gezonder te eten heb ik een duidelijk doel nodig | Helemaal mee oneens  1  2  3  4  5  Helemaal mee eens |

U kunt met een cijfer tussen de 1 en 5 aangeven wat het beste past bij uw ervaring

| [action_plan_79n2w1bh](https://psycore.one/construct/?ucid=action_plan_79n2w1bh) | Om gezonder te eten heb ik een goed plan nodig | Helemaal mee oneens  1  2  3  4  5  Helemaal mee eens |
| --- | --- | --- |
| [capacity_73dnt602](https://psycore.one/construct/?ucid=capacity_73dnt602) | Als ik me voorneem om gezonder te eten dan… | lukt dat erg moeilijk  1  2  3  4  5  lukt dat erg makkelijk |
| [need_79n2fh4r](https://psycore.one/construct/?ucid=need_79n2fh4r) | Om gezonder te eten heb ik veel persoonlijke begeleiding nodig | Helemaal mee oneens  1  2  3  4  5  Helemaal mee eens |
| [need_79n2fh4r](https://psycore.one/construct/?ucid=need_79n2fh4r) | Om gezonder te eten heb ik goed advies nodig | Helemaal mee oneens  1  2  3  4  5  Helemaal mee eens |
| [attitude_73dnt5zc](https://psycore.one/construct/?ucid=attitude_73dnt5zc) | Digitale hulpmiddelen (bijvoorbeeld een smartwatch of een app) kunnen me … helpen om gezonder te eten | niet of nauwelijks  1  2  3  4  5  erg veel |

De volgende vragen gaan over actief en sociaal in het leven staan. Actief in het leven staan kunt u bijvoorbeeld doen door te puzzelen, lezen, creatief bezig zijn, spelletjes te spelen en door nieuwe dingen te leren, zoals een taal. Daarnaast kunt u actief zijn binnen het verenigingsleven, veel ondernemen met vrienden of familie, of iets doen voor een ander zoals vrijwilligerswerk.

| [self_identity_79n2fh4t](https://psycore.one/construct/?ucid=self_identity_79n2fh4t)  [awareness_79n2w1bj](https://psycore.one/construct/?ucid=awareness_79n2w1bj) | Vindt u dat u voldoende sociaal en actief in het leven staat? | Ja Een beetje  Nee |
| --- | --- | --- |
| [self_identity_79n2fh4t](https://psycore.one/construct/?ucid=self_identity_79n2fh4t) | Ik vind dat ik een betekenisvol leven leid | Ja  Een beetje  Nee |
| [self_identity_79n2fh4t](https://psycore.one/construct/?ucid=self_identity_79n2fh4t) | Ik respecteer mezelf en zorg goed voor mezelf | Ja  Een beetje  Nee |
| U kunt met een cijfer tussen de 1 en 5 aangeven wat het beste past bij uw ervaring | | |
| [intention_73dnt604](https://psycore.one/construct/?ucid=intention_73dnt604)  [motivation_79n2fh4q](https://psycore.one/construct/?ucid=motivation_79n2fh4q)  [action_plan_79n2w1bh](https://psycore.one/construct/?ucid=action_plan_79n2w1bh) | Ik ben van plan om … actiever en socialer te gaan leven | niet of nauwelijks  1  2  3  4  5  veel |
| [intention_73dnt604](https://psycore.one/construct/?ucid=intention_73dnt604)  [motivation_79n2fh4q](https://psycore.one/construct/?ucid=motivation_79n2fh4q) | Om het risico op dementie te verminderen ben ik… | niet bereid om veel actiever en socialer te gaan leven  1  2  3  4  5  bereid om veel actiever en socialer te gaan leven |
| [attitude_73dnt5zc](https://psycore.one/construct/?ucid=attitude_73dnt5zc) | Een actief en sociaal leven vind ik… | onplezierig  1  2  3  4  5  plezierig |

| [risk_perception_79n2fh4t](https://psycore.one/construct/?ucid=risk_perception_79n2fh4t) | Actief en sociaal zijn heeft… | erg weinig effect op de gezondheid van mijn hersenen  1  2  3  4  5  erg veel effect op de gezondheid van mijn hersenen |
| --- | --- | --- |
| [inflEnvironment_79n2r0sy](https://psycore.one/construct/?ucid=inflEnvironment_79n2r0sy)  [perceivedBehavioralControl_73dnt603](https://psycore.one/construct/?ucid=perceivedBehavioralControl_73dnt603)  [self_control_79n2fh4t](https://psycore.one/construct/?ucid=self_control_79n2fh4t) | Hoe actief en sociaal ik leef wordt… | niet of nauwelijks beïnvloed door mensen om mij heen  1  2  3  4  5  sterk beïnvloed door mensen om mij heen |
| [perceivedBehavioralControl_73dnt603](https://psycore.one/construct/?ucid=perceivedBehavioralControl_73dnt603)  [self_control_79n2fh4t](https://psycore.one/construct/?ucid=self_control_79n2fh4t) | Hoe actief en sociaal ik leef heb ik… | niet of nauwelijks zelf in de hand  1  2  3  4  5  volledig zelf in de hand |
| [capacity_73dnt602](https://psycore.one/construct/?ucid=capacity_73dnt602) | Als ik me voorneem om actiever en socialer te gaan leven dan… | lukt dat erg moeilijk  1  2  3  4  5  lukt dat erg makkelijk |
| [goal_79n2r0sz](https://psycore.one/construct/?ucid=goal_79n2r0sz) | Om actiever en socialer te leven heb ik een duidelijk doel nodig | Helemaal mee oneens  1  2  3  4  5  Helemaal mee eens |

| [action_plan_79n2w1bh](https://psycore.one/construct/?ucid=action_plan_79n2w1bh) | Om actiever en socialer te leven heb ik goed plan nodig | Helemaal mee oneens  1  2  3  4  5  Helemaal mee eens |
| --- | --- | --- |
| [need_79n2fh4r](https://psycore.one/construct/?ucid=need_79n2fh4r) | Om actiever en socialer te leven heb ik veel persoonlijke begeleiding nodig | Helemaal mee oneens  1  2  3  4  5  Helemaal mee eens |
| [need_79n2fh4r](https://psycore.one/construct/?ucid=need_79n2fh4r) | Om actiever en socialer te leven heb ik goed advies nodig | Helemaal mee oneens  1  2  3  4  5  Helemaal mee eens |
| [attitude_73dnt5zc](https://psycore.one/construct/?ucid=attitude_73dnt5zc) | Digitale hulpmiddelen (bijvoorbeeld een smartwatch of een app) kunnen me … helpen om actiever en socialer in het leven te staan | niet of nauwelijks  1  2  3  4  5  erg veel |

**Conditie**: alcohol drinken ≠ nooit

De volgende vragen gaan over het drinken van alcoholhoudende drank.

| [awareness_79n2w1bj](https://psycore.one/construct/?ucid=awareness_79n2w1bj) | Vindt u dat u overmatig drinkt? | | Ja Een beetje  Nee | |
| --- | --- | --- | --- | --- |
| U kunt met een cijfer tussen de 1 en 5 aangeven wat het beste past bij uw ervaring | | | | |
| [intention_73dnt604](https://psycore.one/construct/?ucid=intention_73dnt604)  [motivation_79n2fh4q](https://psycore.one/construct/?ucid=motivation_79n2fh4q)  [action_plan_79n2w1bh](https://psycore.one/construct/?ucid=action_plan_79n2w1bh) | Ik ben van plan om … minder alcohol te drinken | | niet of nauwelijks  1  2  3  4  5  veel | |
| [intention_73dnt604](https://psycore.one/construct/?ucid=intention_73dnt604)  [motivation_79n2fh4q](https://psycore.one/construct/?ucid=motivation_79n2fh4q) | Om het risico op dementie te verminderen ben ik… | | niet bereid om veel minder alcohol te drinken  1  2  3  4  5  bereid om veel minder alcohol te drinken | |
| [attitude_73dnt5zc](https://psycore.one/construct/?ucid=attitude_73dnt5zc) | Het drinken van alcohol vind ik… | | onplezierig  1  2  3  4  5  plezierig | |
| [awareness_79n2w1bj](https://psycore.one/construct/?ucid=awareness_79n2w1bj) | Het drinken van alcohol vind ik… | | niet tot nauwelijks schadelijk voor mijn gezondheid  1  2  3  4  5  erg schadelijk voor mijn gezondheid | |
| [risk_perception_79n2fh4t](https://psycore.one/construct/?ucid=risk_perception_79n2fh4t)  [awareness_79n2w1bj](https://psycore.one/construct/?ucid=awareness_79n2w1bj) | Het drinken van alcohol vind ik een… | | erg klein gezondheidsrisico  1  2  3  4  5  erg groot gezondheidsrisico | |
| [risk_perception_79n2fh4t](https://psycore.one/construct/?ucid=risk_perception_79n2fh4t) | | Het drinken van alcohol heeft… | | erg weinig effect op de gezondheid van mijn hersenen  1  2  3  4  5  erg veel effect op de gezondheid van mijn hersenen |
| [risk_perception_79n2fh4t](https://psycore.one/construct/?ucid=risk_perception_79n2fh4t)  [instrAttitude_expectation_73dnt5z6](https://psycore.one/construct/?ucid=instrAttitude_expectation_73dnt5z6) | | Het drinken van alcohol verhoogt mijn risico op dementie… | | niet tot nauwelijks  1  2  3  4  5  erg veel |
| [referentBehavior_73dnt5zk](https://psycore.one/construct/?ucid=referentBehavior_73dnt5zk)  [perceivedNorms_73dnt5zq](https://psycore.one/construct/?ucid=perceivedNorms_73dnt5zq) | | Vergeleken met de mensen om mij heen drink ik… | | heel erg weinig alcohol  1  2  3  4  5  heel erg veel alcohol |
| [inflEnvironment_79n2r0sy](https://psycore.one/construct/?ucid=inflEnvironment_79n2r0sy)  [perceivedBehavioralControl_73dnt603](https://psycore.one/construct/?ucid=perceivedBehavioralControl_73dnt603)  [self_control_79n2fh4t](https://psycore.one/construct/?ucid=self_control_79n2fh4t) | | Wanneer en hoeveel alcohol ik drink wordt… | | niet tot nauwelijks beïnvloed door mensen om mij heen  1  2  3  4  5  sterk beïnvloed door mensen om mij heen |
| [habit_79n2r0sz](https://psycore.one/construct/?ucid=habit_79n2r0sz)  [perceivedBehavioralControl_73dnt603](https://psycore.one/construct/?ucid=perceivedBehavioralControl_73dnt603)  [self_control_79n2fh4t](https://psycore.one/construct/?ucid=self_control_79n2fh4t) | | Wanneer en hoeveel alcohol ik drink wordt… | | niet tot nauwelijks beïnvloed door gewoonten  1  2  3  4  5  sterk beïnvloed door gewoonten |
| [perceivedBehavioralControl_73dnt603](https://psycore.one/construct/?ucid=perceivedBehavioralControl_73dnt603)  [self_control_79n2fh4t](https://psycore.one/construct/?ucid=self_control_79n2fh4t) | | Wanneer en hoeveel alcohol ik drink heb ik… | | niet of nauwelijks zelf in de hand  1  2  3  4  5  helemaal zelf in de hand |
| [goal_79n2r0sz](https://psycore.one/construct/?ucid=goal_79n2r0sz) | Om mijn alcohol gebruik te verminderen heb ik een duidelijk doel nodig | | Helemaal mee oneens  1  2  3  4  5  Helemaal mee eens | |
| [action_plan_79n2w1bh](https://psycore.one/construct/?ucid=action_plan_79n2w1bh) | Om mijn alcohol gebruik te verminderen heb ik een goed plan nodig | | Helemaal mee oneens  1  2  3  4  5  Helemaal mee eens | |
| [capacity_73dnt602](https://psycore.one/construct/?ucid=capacity_73dnt602) | Als ik me voorneem om met alcohol te minderen dan… | | lukt dat erg moeilijk  1  2  3  4  5  lukt dat erg makkelijk | |
| [need_79n2fh4r](https://psycore.one/construct/?ucid=need_79n2fh4r) | Om mijn alcoholgebruik te verminderen heb ik veel persoonlijke begeleiding nodig | | Helemaal mee oneens  1  2  3  4  5  Helemaal mee eens | |
| [need_79n2fh4r](https://psycore.one/construct/?ucid=need_79n2fh4r) | Om mijn alcoholgebruik te verminderen heb ik goed advies nodig | | Helemaal mee oneens  1  2  3  4  5  Helemaal mee eens | |
| [attitude_73dnt5zc](https://psycore.one/construct/?ucid=attitude_73dnt5zc) | Digitale hulpmiddelen (bijvoorbeeld een smartwatch of een app) kunnen me … helpen om minder alcohol te drinken | | niet of nauwelijks  1  2  3  4  5  erg veel | |

Wij danken u voor uw deelname! Als u vragen heeft over uw levensstijl, (hersen)gezondheid of dementie dan kunt u dit met uw huisarts bespreekbaar maken. Als u (nog) vragen heeft over het onderzoek dan kunt u contact opnemen met [jeroen.bruinsma@maastrichtuniversity.nl](mailto:jeroen.bruinsma@maastrichtuniversity.nl)

Graag willen wij u nu nog enkele vragen stellen over de vragenlijst die u zojuist heeft ingevuld. Uw mening kan ons helpen toekomstige vragenlijsten verder te verbeteren. Als u deze vraag wilt overslaan, klikt u gewoon op Volgende om door te gaan naar het einde van de vragenlijst.

**Wat vond u van de vragenlijst?**

| interessant onderwerp | o | o | o | o | o | oninteressant onderwerp |
| --- | --- | --- | --- | --- | --- | --- |
| te kort | o | o | o | o | o | te lang |
| duidelijke vragen | o | o | o | o | o | onduidelijke vragen |
| prettig om in te vullen | o | o | o | o | o | onprettig om in te vullen |

**Indien u nog opmerkingen heeft over het onderwerp van deze vragenlijst, kunt u daarvoor de ruimte hieronder gebruiken.**

|  |
| --- |

Pagina 16

Controleer uw gegevens. Indien de gegevens niet meer correct zijn, wordt u na het versturen van de vragenlijst automatisch doorgeleid naar een pagina waar u deze kunt aanpassen.

<paspoortje>

Heel erg bedankt voor uw medewerking! Klik op Volgende om uw antwoorden te versturen.

Klik op ‘volgende’ om deze vragenlijst af te sluiten.
