# Supplementary material for: Socio-Cognitive Determinants of Lifestyle Behavior in the Context of Dementia Risk Reduction: A Population-Based Study in the Netherlands
Source: J Alzheimers Dis. 2024 May 28;99(3):941–52. doi: 10.3233/JAD-231369 (PMC11191482; doi:10.3233/JAD-231369)
Supplement: Supplementary Material — Follow-up questionnaire (English translation) [file jad-99-jad231369-s004.docx]

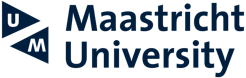

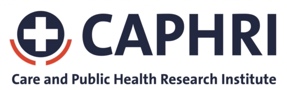
**Second questionnaire**

**Study: Lifestyle, Brain Health, and Dementia**

Thank you for completing the initial questionnaire. Your answers indicate room for improvement in your lifestyle to enhance brain health. By adopting healthier habits, you can reduce the risk of dementia as much as possible.

Maintaining brain health involves regular exercise, healthy eating, staying socially and mentally active, avoiding smoking, and consuming alcohol in moderation or abstaining altogether. In this second questionnaire, we'll ask you a series of questions related to these aspects. It should take approximately 15 to 25 minutes to complete. Upon completing this questionnaire, you will receive an additional [XX] points.

Once again, we kindly ask you to answer all questions honestly. We value your opinions and experiences, and there are no right or wrong answers.

If you have any questions, please contact Dr. Jeroen Bruinsma at [jeroen.bruinsma@maastrichtuniversity.nl](mailto:jeroen.bruinsma@maastrichtuniversity.nl)

If you have any further questions about your lifestyle or (brain) health after completing this questionnaire, feel free to discuss them with your primary care physician.

[**start questionnaire 2**]

For quality purposes, we have the following question for you.

This questionnaire is intended for [name]. Are you this person?

- yes
- no

The first questions pertain to your overall health and lifestyle.

| [awareness_79n2w1bj](https://psycore.one/construct/?ucid=awareness_79n2w1bj) | Do you believe you have a healthy lifestyle? | Yes  Somewhat  No  I don't know |
| --- | --- | --- |
|  | Could you elaborate on why you think this way? |  |
|  |  | |
| You can indicate with a single grade between 1 and 5 what best represents your experience | | |
| [attitude_73dnt5zc](https://psycore.one/construct/?ucid=attitude_73dnt5zc) | A healthy lifestyle to me is... important | not at all or barely  1  2  3  4  5  very much |
| [attitude_73dnt5zc](https://psycore.one/construct/?ucid=attitude_73dnt5zc)  [risk_perception_79n2fh4t](https://psycore.one/construct/?ucid=risk_perception_79n2fh4t) | I find changing my current lifestyle... | unnecessary  1  2  3  4  5  necessary |
| [capacity_73dnt602](https://psycore.one/construct/?ucid=capacity_73dnt602)  [perceivedBehavioralControl_73dnt603](https://psycore.one/construct/?ucid=perceivedBehavioralControl_73dnt603)  [self_control_79n2fh4t](https://psycore.one/construct/?ucid=self_control_79n2fh4t) | If I want to change my lifestyle, it's... | very difficult for me  1  2  3  4  5  very easy for me |
| [risk_perception_79n2fh4t](https://psycore.one/construct/?ucid=risk_perception_79n2fh4t)  [instrAttitude_expectation_73dnt5z6](https://psycore.one/construct/?ucid=instrAttitude_expectation_73dnt5z6) | My risk of getting ill... | has little to do with my lifestyle  1  2  3  4  5  has a lot to do with my lifestyle |

| [risk_perception_79n2fh4t](https://psycore.one/construct/?ucid=risk_perception_79n2fh4t)  [instrAttitude_expectation_73dnt5z6](https://psycore.one/construct/?ucid=instrAttitude_expectation_73dnt5z6) | The risk of developing dementia... | has little to do with my lifestyle  1  2  3  4  5  has a lot to do with my lifestyle |
| --- | --- | --- |
| [intention_73dnt604](https://psycore.one/construct/?ucid=intention_73dnt604)  [motivation_79n2fh4q](https://psycore.one/construct/?ucid=motivation_79n2fh4q) | To reduce the risk of dementia, I am.. | not willing to change my lifestyle  1  2  3  4  5  Willing to change my lifestyle |

Condition (screening questionnaire): Smoking = yes

The following questions are about smoking.

| [intention_73dnt604](https://psycore.one/construct/?ucid=intention_73dnt604)  [motivation_79n2fh4q](https://psycore.one/construct/?ucid=motivation_79n2fh4q)  [action_plan_79n2w1bh](https://psycore.one/construct/?ucid=action_plan_79n2w1bh) | Do you have plans to smoke less? | No  Yes, I want to smoke less  Yes, I want to quit smoking |
| --- | --- | --- |
| You can indicate with a single grade between 1 and 5 what best represents your experience | | |
| [intention_73dnt604](https://psycore.one/construct/?ucid=intention_73dnt604)  [motivation_79n2fh4q](https://psycore.one/construct/?ucid=motivation_79n2fh4q) | To lower my risk for dementia I would... | not/barely be willing to quit with smoking  1  2  3  4  5  be willing to quit with smoking |
| [attitude_73dnt5zc](https://psycore.one/construct/?ucid=attitude_73dnt5zc) | I think smoking is... | not pleasurable  1  2  3  4  5  very pleasurable |
| [awareness_79n2w1bj](https://psycore.one/construct/?ucid=awareness_79n2w1bj) | I think smoking is... | not/barely a risk for my health  1  2  3  4  5  an enormous risk for my health |
| [risk_perception_79n2fh4t](https://psycore.one/construct/?ucid=risk_perception_79n2fh4t) | I think smoking is... | a small health risk  1  2  3  4  5  an enormous health risk |
| [risk_perception_79n2fh4t](https://psycore.one/construct/?ucid=risk_perception_79n2fh4t) | I think smoking has... | not/barely an effect on my brain health  1  2  3  4  5  enormous effect on my brain health |

| [risk_perception_79n2fh4t](https://psycore.one/construct/?ucid=risk_perception_79n2fh4t)  [instrAttitude_expectation_73dnt5z6](https://psycore.one/construct/?ucid=instrAttitude_expectation_73dnt5z6) | If I smoke this will increase my risk for dementia... | not/barely  1  2  3  4  5  enormously |
| --- | --- | --- |
| [inflEnvironment_79n2r0sy](https://psycore.one/construct/?ucid=inflEnvironment_79n2r0sy)  [perceivedBehavioralControl_73dnt603](https://psycore.one/construct/?ucid=perceivedBehavioralControl_73dnt603)  [self_control_79n2fh4t](https://psycore.one/construct/?ucid=self_control_79n2fh4t) | When and how much I smoke is... | not/barely influenced by people around me  1  2  3  4  5  enormously influenced by people around me |
| [habit_79n2r0sz](https://psycore.one/construct/?ucid=habit_79n2r0sz)  [perceivedBehavioralControl_73dnt603](https://psycore.one/construct/?ucid=perceivedBehavioralControl_73dnt603)  [self_control_79n2fh4t](https://psycore.one/construct/?ucid=self_control_79n2fh4t) | When and how much I smoke is... | not/barely influenced by habits 1  2  3  4  5  enormously influenced by habits |
| [perceivedBehavioralControl_73dnt603](https://psycore.one/construct/?ucid=perceivedBehavioralControl_73dnt603)  [self_control_79n2fh4t](https://psycore.one/construct/?ucid=self_control_79n2fh4t) | When and how much I smoke is... | not/barely under my control  1  2  3  4  5  completely under my control |
| [capacity_73dnt602](https://psycore.one/construct/?ucid=capacity_73dnt602) | If I intent to quit smoking this will be... | very difficult  1  2  3  4  5  very easy |
| [goal_79n2r0sz](https://psycore.one/construct/?ucid=goal_79n2r0sz) | To quit smoking, I need a clear goal | Strongly Disagree  1  2  3  4  5  Strongly agree |

| [action_plan_79n2w1bh](https://psycore.one/construct/?ucid=action_plan_79n2w1bh) | To quit smoking, I need a good plan | Strongly Disagree  1  2  3  4  5  Strongly agree |
| --- | --- | --- |
| [need_79n2fh4r](https://psycore.one/construct/?ucid=need_79n2fh4r) | To quit smoking, I need a lot of personal guidance | Strongly Disagree  1  2  3  4  5  Strongly agree |
| [need_79n2fh4r](https://psycore.one/construct/?ucid=need_79n2fh4r) | To quit smoking, I need good advice | Strongly Disagree  1  2  3  4  5  Strongly agree |
| [attitude_73dnt5zc](https://psycore.one/construct/?ucid=attitude_73dnt5zc) | Digital tools (such as a smartwatch or an app) can help me … to quit smoking. | not/barely  1  2  3  4  5  very much |

The following questions are about physical activity. You can engage in various forms of movement, such as walking, swimming, or cycling.

| [awareness_79n2w1bj](https://psycore.one/construct/?ucid=awareness_79n2w1bj) | Do you think you do enough weekly physical activity? | Yes  Somewhat  No |
| --- | --- | --- |
| You can indicate with a single grade between 1 and 5 what best represents your experience | | |
| [intention_73dnt604](https://psycore.one/construct/?ucid=intention_73dnt604)  [motivation_79n2fh4q](https://psycore.one/construct/?ucid=motivation_79n2fh4q)  [action_plan_79n2w1bh](https://psycore.one/construct/?ucid=action_plan_79n2w1bh) | I intent to... | not/barely do more PA  1  2  3  4  5  do a lot more PA |
| [intention_73dnt604](https://psycore.one/construct/?ucid=intention_73dnt604)  [motivation_79n2fh4q](https://psycore.one/construct/?ucid=motivation_79n2fh4q) | To lower my risk for dementia I would... | not/barely willing to do a lot more PA  1  2  3  4  5  willing to do a lot more PA |
| [attitude_73dnt5zc](https://psycore.one/construct/?ucid=attitude_73dnt5zc) | I think doing enough PA is... | not important  1  2  3  4  5  very important |
| [attitude_73dnt5zc](https://psycore.one/construct/?ucid=attitude_73dnt5zc) | I think doing enough PA is... | a waste of time  1  2  3  4  5  a valuable time investment |
| [attitude_73dnt5zc](https://psycore.one/construct/?ucid=attitude_73dnt5zc)  [habit_79n2r0sz](https://psycore.one/construct/?ucid=habit_79n2r0sz) | I think doing enough PA... | goes almost automatically  1  2  3  4  5  is very challenging |

| [perceivedBehavioralControl_73dnt603](https://psycore.one/construct/?ucid=perceivedBehavioralControl_73dnt603)  [attitude_73dnt5zc](https://psycore.one/construct/?ucid=attitude_73dnt5zc) | Doing enough PA every day is... | very difficult  1  2  3  4  5  very easy |
| --- | --- | --- |
| [attitude_73dnt5zc](https://psycore.one/construct/?ucid=attitude_73dnt5zc)  [instrAttitude_expectation_73dnt5z6](https://psycore.one/construct/?ucid=instrAttitude_expectation_73dnt5z6) | To keep physically healthy PA is... | not important  1  2  3  4  5  very important |
| [risk_perception_79n2fh4t](https://psycore.one/construct/?ucid=risk_perception_79n2fh4t) | If I am not physically active this is… | a small health risk  1  2  3  4  5  an enormous health risk |
| [risk_perception_79n2fh4t](https://psycore.one/construct/?ucid=risk_perception_79n2fh4t) | If I am not physically active this has… | not/barely an effect on my brain health  1  2  3  4  5  enormous effect on my brain health |
| [risk_perception_79n2fh4t](https://psycore.one/construct/?ucid=risk_perception_79n2fh4t)  [instrAttitude_expectation_73dnt5z6](https://psycore.one/construct/?ucid=instrAttitude_expectation_73dnt5z6) | If I am not physically active this increases my risk for dementia... | not/barely  1  2  3  4  5  enormously |
| [referentBehavior_73dnt5zk](https://psycore.one/construct/?ucid=referentBehavior_73dnt5zk)  [perceivedNorms_73dnt5zq](https://psycore.one/construct/?ucid=perceivedNorms_73dnt5zq) | Compared to the people around me, I do... | very little PA  1  2  3  4  5  very much PA |

| [inflEnvironment_79n2r0sy](https://psycore.one/construct/?ucid=inflEnvironment_79n2r0sy)  [perceivedBehavioralControl_73dnt603](https://psycore.one/construct/?ucid=perceivedBehavioralControl_73dnt603)  [self_control_79n2fh4t](https://psycore.one/construct/?ucid=self_control_79n2fh4t) | How often I do PA is... | not/barely influenced by people around me  1  2  3  4  5  enormously influenced by people around me |
| --- | --- | --- |
| [attitude_73dnt5zc](https://psycore.one/construct/?ucid=attitude_73dnt5zc) | Being physically active I prefer to do… | alone  with someone  in a group |
| [attitude_73dnt5zc](https://psycore.one/construct/?ucid=attitude_73dnt5zc) | If I do sports, I feel... | diffident  1  2  3  4  5  self-confident |
| [goal_79n2r0sz](https://psycore.one/construct/?ucid=goal_79n2r0sz) | To do enough PA, I need a clear goal | Strongly Disagree  1  2  3  4  5  Strongly agree |
| [action_plan_79n2w1bh](https://psycore.one/construct/?ucid=action_plan_79n2w1bh) | To do enough PA, I need a good plan | Strongly Disagree  1  2  3  4  5  Strongly agree |
| [capacity_73dnt602](https://psycore.one/construct/?ucid=capacity_73dnt602) | If I intent to do more PA this will be... | very difficult  1  2  3  4  5  very easy |

| [need_79n2fh4r](https://psycore.one/construct/?ucid=need_79n2fh4r) | To be more physically active, I need a lot of personal guidance | Strongly Disagree  1  2  3  4  5  Strongly agree |
| --- | --- | --- |
| [need_79n2fh4r](https://psycore.one/construct/?ucid=need_79n2fh4r) | To be more physically active, I need good advice | Strongly Disagree  1  2  3  4  5  Strongly agree |
| [attitude_73dnt5zc](https://psycore.one/construct/?ucid=attitude_73dnt5zc) | Digital tools (such as a smartwatch or an app) can help me … to be more physically active | not or barely  1  2  3  4  5  very much |

The following questions are about healthy eating. Healthy nutrition is rich in vitamins, minerals, and other essential nutrients. Healthy eating includes fresh vegetables, fruits, and whole-grain products. Additionally, consuming (fatty) fish, olive oil, nuts, and legumes is also considered healthy.

| [awareness_79n2w1bj](https://psycore.one/construct/?ucid=awareness_79n2w1bj) | Do you think you eat healthy? | | Yes  Somewhat  No |
| --- | --- | --- | --- |
| You can indicate with a single grade between 1 and 5 what best represents your experience | | | |
| [intention_73dnt604](https://psycore.one/construct/?ucid=intention_73dnt604)  [motivation_79n2fh4q](https://psycore.one/construct/?ucid=motivation_79n2fh4q)  [action_plan_79n2w1bh](https://psycore.one/construct/?ucid=action_plan_79n2w1bh) | I intent to... | | not/barely eat healthier  1  2  3  4  5  eat much healthier |
| [intention_73dnt604](https://psycore.one/construct/?ucid=intention_73dnt604)  [motivation_79n2fh4q](https://psycore.one/construct/?ucid=motivation_79n2fh4q) | To lower my risk for dementia I would... | | not/barely be willing to eat healthier  1  2  3  4  5  willing to eat much healthier |
| [attitude_73dnt5zc](https://psycore.one/construct/?ucid=attitude_73dnt5zc) | I think eating healthy is... | | not tasteful  1  2  3  4  5  very tasteful |
| [attitude_73dnt5zc](https://psycore.one/construct/?ucid=attitude_73dnt5zc) | I think eating healthy is... | | Cheap  1  2  3  4  5  expensive |
| [attitude_73dnt5zc](https://psycore.one/construct/?ucid=attitude_73dnt5zc) | I think eating healthy is... | | not important  1  2  3  4  5  very important |
| [attitude_73dnt5zc](https://psycore.one/construct/?ucid=attitude_73dnt5zc) | | Cooking is... | not my passion  1  2  3  4  5  my passion |
| [attitude_73dnt5zc](https://psycore.one/construct/?ucid=attitude_73dnt5zc) | | Cooking a healthy dinner costs, me... | little time  1  2  3  4  5  very much time |
| [attitude_73dnt5zc](https://psycore.one/construct/?ucid=attitude_73dnt5zc)  [instrAttitude_expectation_73dnt5z6](https://psycore.one/construct/?ucid=instrAttitude_expectation_73dnt5z6) | | To keep my body healthy a healthy dinner is... | not important  1  2  3  4  5  very important |
| [risk_perception_79n2fh4t](https://psycore.one/construct/?ucid=risk_perception_79n2fh4t) | | If I eat unhealthy this is... | a small health risk  1  2  3  4  5  an enormous health risk |
| [risk_perception_79n2fh4t](https://psycore.one/construct/?ucid=risk_perception_79n2fh4t) | | If I eat unhealthy this is... | not/barely an effect on my brain health  1  2  3  4  5  enormous effect on my brain health |
| [risk_perception_79n2fh4t](https://psycore.one/construct/?ucid=risk_perception_79n2fh4t)  [instrAttitude_expectation_73dnt5z6](https://psycore.one/construct/?ucid=instrAttitude_expectation_73dnt5z6) | | If I eat unhealthy this increases my risk for dementia... | not or barely  1  2  3  4  5  enormously |

| [referentBehavior_73dnt5zk](https://psycore.one/construct/?ucid=referentBehavior_73dnt5zk)  [perceivedNorms_73dnt5zq](https://psycore.one/construct/?ucid=perceivedNorms_73dnt5zq) | Compared to the people around me, I eat... | very unhealthy  1  2  3  4  5  very healthy |
| --- | --- | --- |
| [inflEnvironment_79n2r0sy](https://psycore.one/construct/?ucid=inflEnvironment_79n2r0sy)  [perceivedBehavioralControl_73dnt603](https://psycore.one/construct/?ucid=perceivedBehavioralControl_73dnt603)  [self_control_79n2fh4t](https://psycore.one/construct/?ucid=self_control_79n2fh4t) | What and when I eat is... | not/barely influenced by people around me  1  2  3  4  5  enormously influenced by people around me |
| [habit_79n2r0sz](https://psycore.one/construct/?ucid=habit_79n2r0sz)  [perceivedBehavioralControl_73dnt603](https://psycore.one/construct/?ucid=perceivedBehavioralControl_73dnt603)  [self_control_79n2fh4t](https://psycore.one/construct/?ucid=self_control_79n2fh4t) | What and when I eat is... | not/barely influenced by habits  1  2  3  4  5  enormously influenced by habits |
| [inflEnvironment_79n2r0sy](https://psycore.one/construct/?ucid=inflEnvironment_79n2r0sy)  [perceivedBehavioralControl_73dnt603](https://psycore.one/construct/?ucid=perceivedBehavioralControl_73dnt603)  [self_control_79n2fh4t](https://psycore.one/construct/?ucid=self_control_79n2fh4t) | What and when I eat is... | not/barely influenced by my environment  1  2  3  4  5  enormously influenced by my environment |
| [perceivedBehavioralControl_73dnt603](https://psycore.one/construct/?ucid=perceivedBehavioralControl_73dnt603)  [self_control_79n2fh4t](https://psycore.one/construct/?ucid=self_control_79n2fh4t) | What and when I eat is... | not/barely under my control  1  2  3  4  5  completely under my control |
| [goal_79n2r0sz](https://psycore.one/construct/?ucid=goal_79n2r0sz) | To eat healthier, I need a clear goal | Strongly Disagree  1  2  3  4  5  Strongly agree |

| [action_plan_79n2w1bh](https://psycore.one/construct/?ucid=action_plan_79n2w1bh) | To eat healthier, I need a good plan | Strongly Disagree  1  2  3  4  5  Strongly agree |
| --- | --- | --- |
| [capacity_73dnt602](https://psycore.one/construct/?ucid=capacity_73dnt602) | If I intent to eat healthier this will be... | very difficult  1  2  3  4  5  very easy |
| [need_79n2fh4r](https://psycore.one/construct/?ucid=need_79n2fh4r) | To eat healthier, I need a lot of personal guidance | Strongly Disagree  1  2  3  4  5  Strongly agree |
| [need_79n2fh4r](https://psycore.one/construct/?ucid=need_79n2fh4r) | To eat healthier, I need good advice | Strongly Disagree  1  2  3  4  5  Strongly agree |
| [attitude_73dnt5zc](https://psycore.one/construct/?ucid=attitude_73dnt5zc) | Digital tools (such as a smartwatch or an app) can help me … to eat healthier | not or barely  1  2  3  4  5  very much |

The following questions are about being active and socially engaged in life. Being active in life can involve activities such as solving puzzles, reading, engaging in creative pursuits, playing games, and learning new things like a language. Additionally, being socially active can include participating in community groups, spending time with friends or family, or engaging in activities that involve helping others, such as volunteering.

| [self_identity_79n2fh4t](https://psycore.one/construct/?ucid=self_identity_79n2fh4t)  [awareness_79n2w1bj](https://psycore.one/construct/?ucid=awareness_79n2w1bj) | Do you think that you are socially and actively engaged in life? | Yes  Somewhat  No |
| --- | --- | --- |
| [self_identity_79n2fh4t](https://psycore.one/construct/?ucid=self_identity_79n2fh4t) | I believe I lead a meaningful life | Yes  Somewhat  No |
| [self_identity_79n2fh4t](https://psycore.one/construct/?ucid=self_identity_79n2fh4t) | I respect myself and take good care of myself | Yes  Somewhat  No |
| You can indicate with a single grade between 1 and 5 what best represents your experience | | |
| [intention_73dnt604](https://psycore.one/construct/?ucid=intention_73dnt604)  [motivation_79n2fh4q](https://psycore.one/construct/?ucid=motivation_79n2fh4q)  [action_plan_79n2w1bh](https://psycore.one/construct/?ucid=action_plan_79n2w1bh) | I intent to... | not/barely be more active and social  1  2  3  4  5  be more active and social |
| [intention_73dnt604](https://psycore.one/construct/?ucid=intention_73dnt604)  [motivation_79n2fh4q](https://psycore.one/construct/?ucid=motivation_79n2fh4q) | To lower my risk for dementia I would... | not/barely be willing to be more active and social  1  2  3  4  5  be willing to be more active and social |
| [attitude_73dnt5zc](https://psycore.one/construct/?ucid=attitude_73dnt5zc) | An active and social life is... | not pleasurable  1  2  3  4  5  pleasurable |

| [risk_perception_79n2fh4t](https://psycore.one/construct/?ucid=risk_perception_79n2fh4t) | An active and social life has… | not/barely an effect on my brain health  1  2  3  4  5  enormous effect on my brain health |
| --- | --- | --- |
| [inflEnvironment_79n2r0sy](https://psycore.one/construct/?ucid=inflEnvironment_79n2r0sy)  [perceivedBehavioralControl_73dnt603](https://psycore.one/construct/?ucid=perceivedBehavioralControl_73dnt603)  [self_control_79n2fh4t](https://psycore.one/construct/?ucid=self_control_79n2fh4t) | When and how active and social I am is… | not/barely influence by people around me  1  2  3  4  5  enormously influenced by people around me |
| [perceivedBehavioralControl_73dnt603](https://psycore.one/construct/?ucid=perceivedBehavioralControl_73dnt603)  [self_control_79n2fh4t](https://psycore.one/construct/?ucid=self_control_79n2fh4t) | When and how active and social I am is… | not/barely under my control  1  2  3  4  5  completely under my control |
| [capacity_73dnt602](https://psycore.one/construct/?ucid=capacity_73dnt602) | If I intent to be more active and social this will be... | very difficult  1  2  3  4  5  very easy |
| [goal_79n2r0sz](https://psycore.one/construct/?ucid=goal_79n2r0sz) | To have a more active and social life, I need a clear goal | Strongly Disagree  1  2  3  4  5  Strongly agree |

| [action_plan_79n2w1bh](https://psycore.one/construct/?ucid=action_plan_79n2w1bh) | To have a more active and social life, I need a good plan | Strongly Disagree  1  2  3  4  5  Strongly agree |
| --- | --- | --- |
| [need_79n2fh4r](https://psycore.one/construct/?ucid=need_79n2fh4r) | To have a more active and social life, I need a lot of personal guidance | Strongly Disagree  1  2  3  4  5  Strongly agree |
| [need_79n2fh4r](https://psycore.one/construct/?ucid=need_79n2fh4r) | To have a more active and social life, I need good advice | Strongly Disagree  1  2  3  4  5  Strongly agree |
| [attitude_73dnt5zc](https://psycore.one/construct/?ucid=attitude_73dnt5zc) | Digital tools (such as a smartwatch or an app) can help me … to have a more active and social life | not or barely  1  2  3  4  5  very much |

Condition: Drinking alcohol ≠ never

The following questions are about consuming alcoholic beverages.

| [awareness_79n2w1bj](https://psycore.one/construct/?ucid=awareness_79n2w1bj) | Do you think you overconsume alcohol? | | Yes  Sometimes  No | |
| --- | --- | --- | --- | --- |
| You can indicate with a single grade between 1 and 5 what best represents your experience | | | | |
| [intention_73dnt604](https://psycore.one/construct/?ucid=intention_73dnt604)  [motivation_79n2fh4q](https://psycore.one/construct/?ucid=motivation_79n2fh4q)  [action_plan_79n2w1bh](https://psycore.one/construct/?ucid=action_plan_79n2w1bh) | I intent to... | | not/barely decrease my alcohol intake  1  2  3  4  5  decrease my alcohol intake a lot | |
| [intention_73dnt604](https://psycore.one/construct/?ucid=intention_73dnt604)  [motivation_79n2fh4q](https://psycore.one/construct/?ucid=motivation_79n2fh4q) | To lower my risk for dementia I would... | | not/barely be willing to decrease my alcohol intake  1  2  3  4  5  be willing to decrease my alcohol intake a lot | |
| [attitude_73dnt5zc](https://psycore.one/construct/?ucid=attitude_73dnt5zc) | Drinking alcohol is... | | not pleasurable  1  2  3  4  5  pleasurable | |
| [awareness_79n2w1bj](https://psycore.one/construct/?ucid=awareness_79n2w1bj) | Drinking alcohol is... | | not/barely a risk for my health  1  2  3  4  5  an enormous risk for my health | |
| [risk_perception_79n2fh4t](https://psycore.one/construct/?ucid=risk_perception_79n2fh4t)  [awareness_79n2w1bj](https://psycore.one/construct/?ucid=awareness_79n2w1bj) | Drinking alcohol is a... | | small health risk  1  2  3  4  5  enormous health risk | |
| [risk_perception_79n2fh4t](https://psycore.one/construct/?ucid=risk_perception_79n2fh4t) | | Drinking alcohol has... | | not/barely an effect on my brain health  1  2  3  4  5  enormous effect on my brain health |
| [risk_perception_79n2fh4t](https://psycore.one/construct/?ucid=risk_perception_79n2fh4t)  [instrAttitude_expectation_73dnt5z6](https://psycore.one/construct/?ucid=instrAttitude_expectation_73dnt5z6) | | Drinking alcohol increases my risk for dementia... | | not/barely  1  2  3  4  5  enormously |
| [referentBehavior_73dnt5zk](https://psycore.one/construct/?ucid=referentBehavior_73dnt5zk)  [perceivedNorms_73dnt5zq](https://psycore.one/construct/?ucid=perceivedNorms_73dnt5zq) | | Compared to the people around me, I drink... | | very little alcohol  1  2  3  4  5  very much alcohol |
| [inflEnvironment_79n2r0sy](https://psycore.one/construct/?ucid=inflEnvironment_79n2r0sy)  [perceivedBehavioralControl_73dnt603](https://psycore.one/construct/?ucid=perceivedBehavioralControl_73dnt603)  [self_control_79n2fh4t](https://psycore.one/construct/?ucid=self_control_79n2fh4t) | | When and how much alcohol I drink is... | | not/barely influenced by people around me  1  2  3  4  5  enormously influenced by people around me |
| [habit_79n2r0sz](https://psycore.one/construct/?ucid=habit_79n2r0sz)  [perceivedBehavioralControl_73dnt603](https://psycore.one/construct/?ucid=perceivedBehavioralControl_73dnt603)  [self_control_79n2fh4t](https://psycore.one/construct/?ucid=self_control_79n2fh4t) | | When and how much alcohol I drink is... | | not/barely influenced by habits  1  2  3  4  5  enormously influenced by habits |
| [perceivedBehavioralControl_73dnt603](https://psycore.one/construct/?ucid=perceivedBehavioralControl_73dnt603)  [self_control_79n2fh4t](https://psycore.one/construct/?ucid=self_control_79n2fh4t) | | When and how much alcohol I drink is... | | not/barely under my control  1  2  3  4  5  completely under my control |
| [goal_79n2r0sz](https://psycore.one/construct/?ucid=goal_79n2r0sz) | To decrease my alcohol intake, I need a clear goal | | Strongly Disagree  1  2  3  4  5  Strongly agree | |
| [action_plan_79n2w1bh](https://psycore.one/construct/?ucid=action_plan_79n2w1bh) | To decrease my alcohol intake, I need a good plan | | Strongly Disagree  1  2  3  4  5  Strongly agree | |
| [capacity_73dnt602](https://psycore.one/construct/?ucid=capacity_73dnt602) | Decreasing my alcohol consumption will be... | | very difficult  1  2  3  4  5  very easy | |
| [need_79n2fh4r](https://psycore.one/construct/?ucid=need_79n2fh4r) | To decrease my alcohol intake, I need a lot of personal guidance | | Strongly Disagree  1  2  3  4  5  Strongly agree | |
| [need_79n2fh4r](https://psycore.one/construct/?ucid=need_79n2fh4r) | To decrease my alcohol intake, I need good advice | | Strongly Disagree  1  2  3  4  5  Strongly agree | |
| [attitude_73dnt5zc](https://psycore.one/construct/?ucid=attitude_73dnt5zc) | Digital tools (such as a smartwatch or an app) can help me … to decrease my alcohol intake | | not or barely  1  2  3  4  5  very much | |

We thank you for your participation! If you have any questions regarding your lifestyle, (brain) health, or dementia, please discuss them with your primary care physician. For any further inquiries regarding the research, feel free to contact [jeroen.bruinsma@maastrichtuniversity.nl](mailto:jeroen.bruinsma@maastrichtuniversity.nl)

We would like to ask you a few more questions about the questionnaire you have just completed. Your feedback can help us further improve future questionnaires. If you wish to skip this question, simply click 'Next' to proceed to the end of the questionnaire.

What did you think of the questionnaire?

| interesting topic | o | o | o | o | o | uninteresting topic |
| --- | --- | --- | --- | --- | --- | --- |
| too short | o | o | o | o | o | too long |
| clear questions | o | o | o | o | o | unclear questions |
| pleasant to fill out | o | o | o | o | o | unpleasant to fill out |

**If you have any additional comments regarding the topic of this questionnaire, please use the space below.**

|  |
| --- |

Please review your details. If the information is no longer correct, after submitting the questionnaire, you will be automatically redirected to a page where you can make changes.

[link to personal profile]

Thank you very much for your cooperation! Click 'Next' to submit your answers.

Click 'next' to finish this questionnaire.
