# Supplementary material for: Socio-Cognitive Determinants of Lifestyle Behavior in the Context of Dementia Risk Reduction: A Population-Based Study in the Netherlands
Source: J Alzheimers Dis. 2024 May 28;99(3):941–52. doi: 10.3233/JAD-231369 (PMC11191482; doi:10.3233/JAD-231369)
Supplement: Supplementary Material — Flycatcher Research Report follow-up questionnaire [file jad-99-jad231369-s005.pdf]

Onderzoeksverantwoording levensstijl en hersengezond-heid meting 2

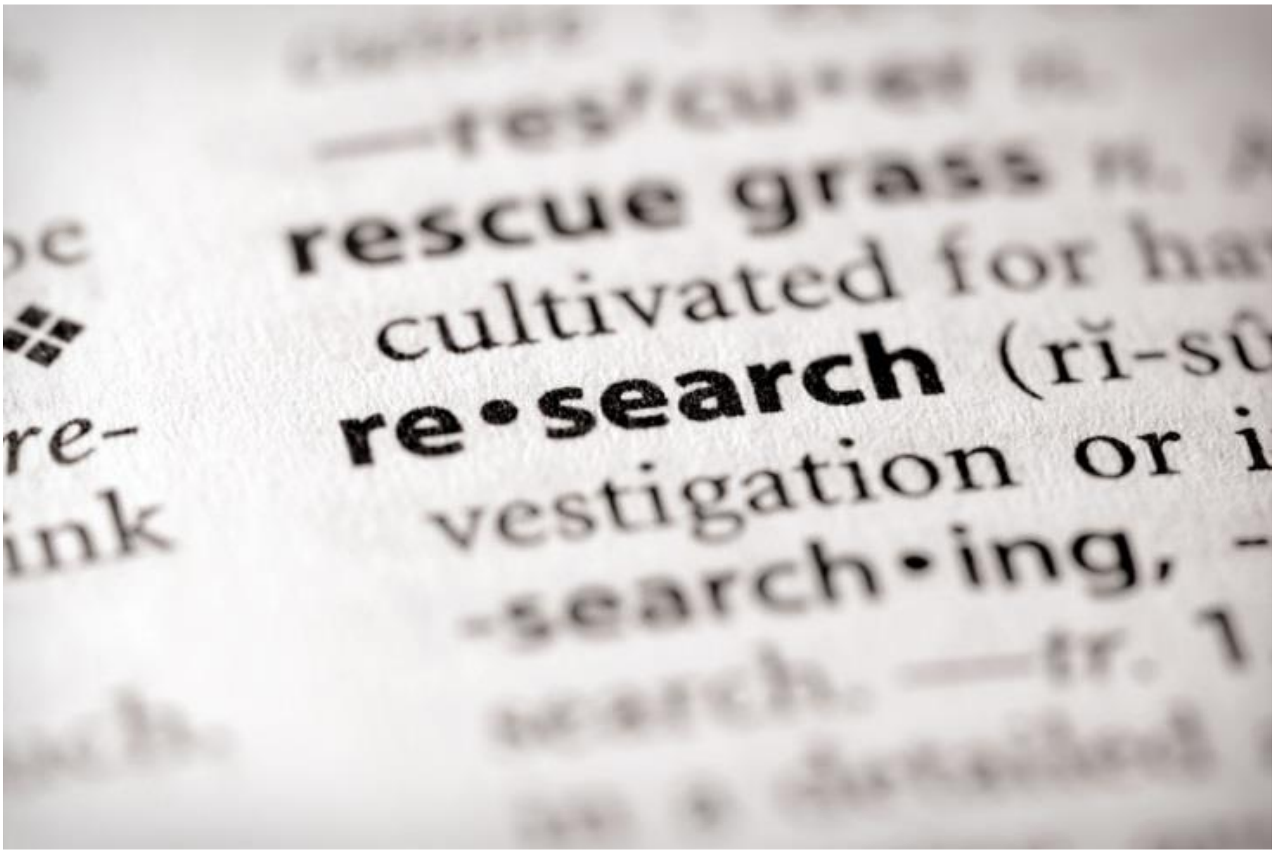

Voor de Universiteit Maastricht (UM) heeft Flycatcher de dataverzameling uitgevoerd voor de tweede meting van een onderzoek naar levensstijl en hersengezondheid.
In totaal hebben 3065 respondenten zowel de eerste als de tweede meting volledig ingevuld.

Postbus 380
6200 AJ Maastricht

tel. 043-326 29 92
info@flycatcher.nl
www.flycatcher.eu

volg ons op twitter:
@FlycatcherLive

© Flycatcher Internet Research, 2022
Dit document is auteursrechtelijk beschermd. Dit document en het middels deze opdracht verkregen databestand zijn enkel voor eigen gebruik door onze formele opdrachtgever. Publicatie van dit document en/of het databestand, inzage hiervan aan derden (geheel of gedeeltelijk) of het doorverkopen van de data is zonder schriftelijke toestemming van Flycatcher beslist niet toegestaan. Flycatcher kan niet verantwoordelijk gehouden worden voor de verdere verwerking en interpretatie van de data en de publicatie van resultaten.

Onderzoeksmethode

Voor de dataverzameling is gebruik gemaakt van een online vragenlijst. De geselecteerde respondenten zijn benaderd via e-mail om mee te doen aan het onderzoek. Door te klikken op een, voor elke respondent unieke, hyperlink in de uitnodiging kwam men bij de vragenlijst terecht. Respondenten beantwoordden eerst een verificatievraag, waarmee zoveel mogelijk voorkomen is dat eventuele huisgenoten of anderen de vragenlijst invulden. De vragenlijst was alleen toegankelijk voor panelleden die hiervoor een uitnodiging kregen.

Onderzoeksgroep

De doelgroep van het onderzoek bestond uit Nederlanders van 40 t/m 79 jaar. Voor het bepalen van de onderzoeksgroep voor de eerste meting is een steekproef geselecteerd uit ons ISO-gecertificeerde Flycatcher panel. Alleen respondenten die de eerste vragenlijst volledig en serieus hadden ingevuld en die volgens de UM uitgenodigd mochten worden voor de tweede meting zijn uitgenodigd voor de tweede meting.

Het Flycatcher panel

Ons panel bestaat uit meer dan 10.000 mensen van 18 jaar en ouder die zich via ‘double-active-opt-in’ vrijwillig en actief bereid hebben verklaard om deel te nemen aan online onderzoeken. Panelleden ontvangen voor elke volledig ingevulde vragenlijst een vooraf bepaald aantal punten en 1 lot in de Flycatcher Kwartaal Loterij. Indien men voldoende punten gespaard heeft, kunnen deze ingewisseld worden voor een cadeaubon. Panelleden spelen met het aantal verdiende loten tevens mee in de Flycatcher Kwartaal Loterij, waarbij onder andere deelname aan onderzoeken de winkans in de loterij bepaalt.

Deelname aan elk afzonderlijk onderzoek is geheel vrijwillig en panelleden kunnen op elk gewenst moment hun lidmaatschap van het panel beëindigen. Panelleden kunnen niet kiezen voor welk soort onderzoeken zij wel of niet uitgenodigd willen worden.

Op aanvraag wordt een document beschikbaar gesteld met een verantwoording van het Flycatcher panel. Hierin is alle informatie over de kwaliteit van het panel te vinden. Deze informatie kan gebruikt worden ter verantwoording aan tijdschriften waarin de onderzoeksresultaten gepubliceerd worden of ten behoeve van de Medisch Ethische Commissie. De verantwoording is desgewenst in het Engels beschikbaar.

De vragenlijst

De vragenlijst is opgesteld door de UM in overleg met Flycatcher. De definitieve vragenlijst is vervolgens door Flycatcher online geprogrammeerd. Om te verhinderen dat respondenten vragen oversloegen of onmogelijke antwoorden invulden, zijn hierbij de volgende regels toegepast:

- alle vragen moesten verplicht ingevuld worden om de vragenlijst te kunnen insturen;
- wijzigen van antwoorden was toegestaan;
- er is routing toegepast zodat respondenten vragen die niet van toepassing waren op hun situatie automatisch oversloegen.

De vragenlijst is door twee onderzoekers (anders dan de verantwoordelijke onderzoeker) inhoudelijk en technisch uitgebreid intern gepretest. Daarnaast heeft Flycatcher de UM de mogelijkheid geboden om de gedigitaliseerde vragenlijst via een proefaccount in te zien en te doorlopen, voordat deze verstuurd is. De online vragenlijst is verstuurd na definitief akkoord van de UM.

Veldwerkperiode en respons

De vragenlijst is verstuurd op maandag 10 oktober en kon ingevuld worden tot maandag 17 oktober 2022. Op donderdag 13 oktober is een reminder gestuurd naar alle panelleden die op dat moment de vragenlijst nog niet of niet volledig ingevuld hadden.

Een overzicht van de respons is te vinden in de volgende tabel.

Responsoverzicht:	
aantal panelleden in steekproef	3745
foutmeldingen (onjuist e-mailadres, mailbox vol)	0
netto verstuurd (= aantal steekproef - foutmeldingen)	3745
*verwijderd wegens slechte responskwaliteit**	14
*vragenlijst onvolledig ingevuld / drop-out**	88
respons	3065
respons % (= respons / netto verstuurd)	81,8%

* De gegevens van deze respondenten zijn niet meegenomen in de respons en de resultaten.

Resultaten

Na het afsluiten van de veldwerkperiode zijn de data opgeschoond, gecontroleerd en verwerkt. Er zijn kwaliteitscontroles uitgevoerd op open antwoorden, consistentie van antwoorden, straightlining en invultijd. Van straightlining is sprake indien bij een reeks stellingen overal dezelfde antwoordoptie gekozen wordt. In geval van een slechte responskwaliteit zijn de resultaten van het betreffende panellid uit het databestand verwijderd (zie responsoverzicht). Panelleden bij wie meerdere malen een slechte responskwaliteit wordt waargenomen, worden uit het panel verwijderd.

De resultaten van het onderzoek zijn aangeleverd in een SPSS-databestand. Het databestand is voorzien van duidelijke labels.

Voor het beschikbaar stellen van het databestand aan of het gebruik van data van onze panelleden door de UM is geen toestemming nodig van de respondenten, aangezien de leden van het Flycatcher panel bij inschrijving akkoord zijn gegaan met de deelnamevoorwaarden. Hierin staat beschreven dat in het kader van (wetenschappelijk) onderzoek data mag worden gedeeld met en/of gebruikt door de opdrachtgever, mits gecategoriseerd, anoniem en niet herleidbaar. Tot slot dient voor het delen en/of gebruik van de data met of door derden of in het geval van publicatie toestemming te worden gevraagd aan Flycatcher.

Evaluatie vragenlijst

Aan het einde van de vragenlijst is aan de respondenten gevraagd wat zij van de vragenlijst vonden. Het resultaat hiervan is weergegeven in de volgende grafiek. Hierin is tevens te zien hoe de vragenlijst beoordeeld werd ten opzichte van andere vragenlijsten die de panelleden ingevuld hebben.

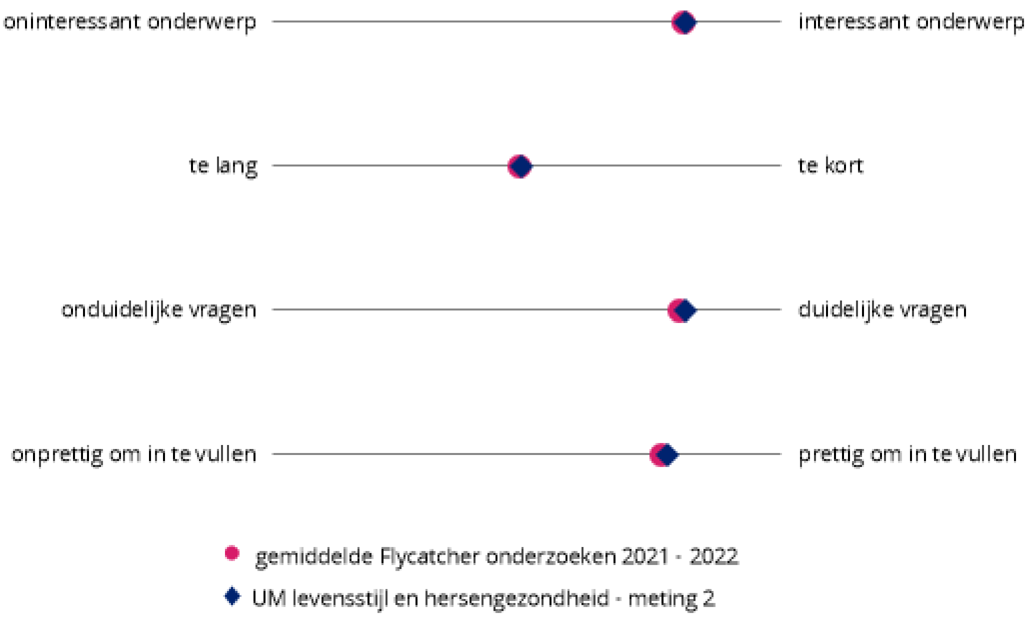

Over Flycatcher

Onderzoeksbureau Flycatcher is in 2000 ontstaan als spin-off van de Universiteit Maastricht. Inmiddels is Flycatcher uitgegroeid tot een middelgroot onderzoeksbureau met een enthousiast team van academisch geschoolde onderzoekers en innovatieve IT-specialisten. Het uitgangspunt is dat onderzoek aantoonbaar moet bijdragen aan het nemen van de juiste beslissingen op basis van betrouwbare, compacte en beleidsrelevante informatie. In onze visie zijn kennis en inzicht onmiskenbare succesfactoren voor alle organisaties, zowel profit als non-profit.

We zijn actief in diverse sectoren en bestrijken een breed onderzoeksveld van evaluatie-onderzoek en werkbelevingsonderzoek tot communicatie-onderzoek en klanttevredenheidsonderzoek. Wij denken graag mee over de opzet en uitvoering van een onderzoek, bijvoorbeeld in de vorm van advies over de wijze van benadering van respondenten, de wijze van dataverzameling (bijv. online of face-to-face), het opstellen van de vragenlijst of het uitvoeren van statistische analyses. Voor elke vraag zoeken we naar de beste oplossing waar onze opdrachtgevers verder mee kunnen.

Wij zijn lid van de MOA en van de wereldwijde branchevereniging van onderzoeksbureaus ESOMAR. Verder zijn we kennispartner van de Stichting INK en VNG Realisatie en sponsoren wij de NPSO (Nederlandstalig Platform voor Survey Onderzoek).

Kwaliteitseisen onderzoek

Flycatcher is in het bezit van een kwaliteitskeurmerk voor markt-, opinie- en maatschappelijk onderzoek (ISO 20252). Het keurmerk bevestigt dat de onderzoeksactiviteiten van Flycatcher, inclusief het Flycatcher panel, voldoen aan de ISO-kwaliteitseisen met betrekking tot onder andere vertrouwelijkheid van onderzoek, competenties en opleiding van de medewerkers, transparantie en richtlijnen voor alle aspecten van een onderzoekstraject (van projectplanning en vragenlijsten tot rapportage). Wij zijn tevens gecertificeerd volgens de milieunorm ISO 14001.

Privacybescherming en beveiliging

Wij hanteren hoge normen als het gaat om de bescherming van privacy. Wij onderschrijven de Integriteitscode van de MOA en hanteren de 10 regels uit de Fair Data Privacy Code. Verder zijn alle medewerkers middels hun contract gebonden aan geheimhoudingsplicht wat betreft vertrouwelijke informatie van opdrachtgevers, vertrouwelijke onderzoeksresultaten en individuele antwoorden van respondenten.

Informatie en data vormen de kern van onze activiteiten. Wij hebben onze werkzaamheden daarom tevens vastgelegd in strenge procedures conform ISO 27001. Deze internationaal erkende ISO-norm is het kwaliteitskeurmerk voor informatiebeveiliging. Het borgt de kwaliteit van de beveiliging en is een uitstekend middel om de beveiliging te beheren en voortdurend te verbeteren. Flycatcher maakt bijvoorbeeld gebruik van “secure server”-technologie (SSL-encryptie). Dit betekent dat al het dataverkeer versleuteld wordt.

FLYCATCHER
Internet Research

Verder door vragen

www.flycatcher.eu

Postbus 380 6200 AJ Maastricht tel. 043 326 29 92 info@flycatcher.nl

Volg ons op Twitter @FlycatcherLive
