# Supplementary material for: Socio-Cognitive Determinants of Lifestyle Behavior in the Context of Dementia Risk Reduction: A Population-Based Study in the Netherlands
Source: J Alzheimers Dis. 2024 May 28;99(3):941–52. doi: 10.3233/JAD-231369 (PMC11191482; doi:10.3233/JAD-231369)
Supplement: Supplementary Material — 1. Section 3.1 Sample characteristics [file jad-99-jad231369-s007.pdf]

```
 ***************
Result paragraph 3.1 Sample Characteristics
***************

COMPUTE inclusion_total=sum (inclusion_smoke, inclusion_alcohol, inclusion_
physical_activity_norm,
    inclusion_MEDAS, inclusion_activities_week).
EXECUTE.

RECODE inclusion_total (0=0) (1=1) (2=1) (3=1) (4=1) (5=1) (SYSMIS=SYSMIS)
INTO
    Improve_one_or_more_domains.
VARIABLE LABELS  Improve_one_or_more_domains 'can improve at least one life
style behavior to '+
    'lower future risk for dementia'.
EXECUTE.

RECODE inclusion_total (0=0) (1=0) (2=1) (3=1) (4=1) (5=1) (SYSMIS=SYSMIS)
INTO
    Improve_two_or_more_domains.
VARIABLE LABELS  Improve_one_or_more_domains 'can improve at least one life
style behavior to '+
    'lower future risk for dementia'.
EXECUTE.

FREQUENCIES VARIABLES=inclusion_total Improve_one_or_more_domains Improve_t
wo_or_more_domains
  /ORDER=ANALYSIS.
```

Frequencies

Notes

Output Created		11-OCT-2023 10:42:...
Comments		
Input	Data	/Users/jeroenbruinsma/surfdrive - Bruinsma, Jeroen (GB)@surfdrive.surf.nl/analyze/11.CIBER.sav
	Active Dataset	DataSet1
	Filter	<none>
	Weight	<none>
	Split File	<none>
	N of Rows in Working Data File	4104
Missing Value Handling	Definition of Missing	User-defined missing values are treated as missing.
	Cases Used	Statistics are based on all cases with valid data.
Syntax		FREQUENCIES VARIABLES=inclusion_total Improve_one_or_more_domains Improve_two_or_more_domains /ORDER=ANALYSIS.
Resources	Processor Time	00:00:00.19
	Elapsed Time	00:00:00.00

Statistics

		inclusion_total	can improve at least one lifestyle behavior to lower future risk for dementia	Improve_two_or_more_domains
N	Valid	4104	4104	4104
	Missing	0	0	0

Frequency Table

inclusion_total

		Frequency	Percent	Valid Percent	Cumulative Percent
Valid	.00	358	8.7	8.7	8.7
	1.00	1080	26.3	26.3	35.0
	2.00	1444	35.2	35.2	70.2
	3.00	951	23.2	23.2	93.4
	4.00	249	6.1	6.1	99.5
	5.00	22	.5	.5	100.0
	Total	4104	100.0	100.0	

can improve at least one lifestyle behavior to lower future risk for dementia

		Frequency	Percent	Valid Percent	Cumulative Percent
Valid	.00	358	8.7	8.7	8.7
	1.00	3746	91.3	91.3	100.0
	Total	4104	100.0	100.0	

Improve_two_or_more_domains

		Frequency	Percent	Valid Percent	Cumulative Percent
Valid	.00	1438	35.0	35.0	35.0
	1.00	2666	65.0	65.0	100.0
	Total	4104	100.0	100.0	

```
***************
Result paragraph 3.1 Sample Characteristics --> for inclusion rates see sup
plementary materials 'onderzoeksverantwoording1 and 2".
***************.
```
