# Supplementary material for: Socio-Cognitive Determinants of Lifestyle Behavior in the Context of Dementia Risk Reduction: A Population-Based Study in the Netherlands
Source: J Alzheimers Dis. 2024 May 28;99(3):941–52. doi: 10.3233/JAD-231369 (PMC11191482; doi:10.3233/JAD-231369)
Supplement: Supplementary Material — 2. Section 3.1 Table 1 [file jad-99-jad231369-s008.pdf]

```
FREQUENCIES VARIABLES=age
  /NTILES=4
  /STATISTICS=MINIMUM MAXIMUM MEAN MEDIAN STDDEV
  /ORDER=ANALYSIS.
```

Frequencies

Notes

Output Created		10-OCT-2023 15:27:...
Comments		
Input	Data	/Users/jeroenbruinsma/surfdrive - Bruinsma, Jeroen (GB)@surfdrive.surf.nl/analyze/11. CIBER.sav
	Active Dataset	DataSet1
	Filter	<none>
	Weight	<none>
	Split File	<none>
	N of Rows in Working Data File	4104
Missing Value Handling	Definition of Missing	User-defined missing values are treated as missing.
	Cases Used	Statistics are based on all cases with valid data.
Syntax		FREQUENCIES VARIABLES=age /NTILES=4 /STATISTICS=MINIMUM MAXIMUM MEAN MEDIAN STDDEV /ORDER=ANALYSIS.
Resources	Processor Time	00:00:00.19
	Elapsed Time	00:00:00.00

Statistics

age in years

N	Valid	4104
	Missing	0
Mean		59.13
Median		59.00
Std. Deviation		10.573
Minimum		40
Maximum		79
Percentiles	25	51.00
	50	59.00
	75	68.00

age in years

		Frequency	Percent	Valid Percent	Cumulative Percent
Valid	40	94	2.3	2.3	2.3
	41	94	2.3	2.3	4.6
	42	97	2.4	2.4	6.9
	43	101	2.5	2.5	9.4
	44	87	2.1	2.1	11.5
	45	78	1.9	1.9	13.4
	46	96	2.3	2.3	15.8
	47	71	1.7	1.7	17.5
	48	100	2.4	2.4	19.9
	49	85	2.1	2.1	22.0
	50	112	2.7	2.7	24.7
	51	97	2.4	2.4	27.1
	52	116	2.8	2.8	29.9
	53	106	2.6	2.6	32.5
	54	107	2.6	2.6	35.1
	55	134	3.3	3.3	38.4
	56	138	3.4	3.4	41.7
	57	98	2.4	2.4	44.1
	58	114	2.8	2.8	46.9
	59	128	3.1	3.1	50.0
	60	100	2.4	2.4	52.5
	61	138	3.4	3.4	55.8
	62	126	3.1	3.1	58.9
	63	132	3.2	3.2	62.1
	64	116	2.8	2.8	64.9
	65	125	3.0	3.0	68.0
	66	125	3.0	3.0	71.0

age in years

	Frequency	Percent	Valid Percent	Cumulative Percent
67	121	2.9	2.9	74.0
68	122	3.0	3.0	76.9
69	124	3.0	3.0	80.0
70	136	3.3	3.3	83.3
71	103	2.5	2.5	85.8
72	96	2.3	2.3	88.1
73	109	2.7	2.7	90.8
74	79	1.9	1.9	92.7
75	83	2.0	2.0	94.7
76	76	1.9	1.9	96.6
77	56	1.4	1.4	98.0
78	47	1.1	1.1	99.1
79	37	.9	.9	100.0
Total	4104	100.0	100.0	

```
FREQUENCIES VARIABLES=gender
  /ORDER=ANALYSIS.
```

Frequencies

Notes

Output Created		10-OCT-2023 15:27:...
Comments		
Input	Data	/Users/jeroenbruinsma/surfdrive - Bruinsma, Jeroen (GB)@surfdrive.surf.nl/analyze/11.CIBER.sav
	Active Dataset	DataSet1
	Filter	<none>
	Weight	<none>
	Split File	<none>
	N of Rows in Working Data File	4104
Missing Value Handling	Definition of Missing	User-defined missing values are treated as missing.
	Cases Used	Statistics are based on all cases with valid data.
Syntax		FREQUENCIES VARIABLES=gender /ORDER=ANALYSIS.
Resources	Processor Time	00:00:00.20
	Elapsed Time	00:00:00.00

Statistics

gender

N	Valid	4104
	Missing	0

gender

		Frequency	Percent	Valid Percent	Cumulative Percent
Valid	Male	1710	41.7	41.7	41.7
	Female	2394	58.3	58.3	100.0
	Total	4104	100.0	100.0	

```
FREQUENCIES VARIABLES=migrant second_gen_migrant
  /ORDER=ANALYSIS.
```

Frequencies

Notes

Output Created		10-OCT-2023 15:28:...
Comments		
Input	Data	/Users/jeroenbruinsma/surfdrive - Bruinsma, Jeroen (GB)@surfdrive.surf.nl/analyze/11.CIBER.sav
	Active Dataset	DataSet1
	Filter	<none>
	Weight	<none>
	Split File	<none>
	N of Rows in Working Data File	4104
Missing Value Handling	Definition of Missing	User-defined missing values are treated as missing.
	Cases Used	Statistics are based on all cases with valid data.
Syntax		FREQUENCIES VARIABLES=migrant second_gen_migrant /ORDER=ANALYSIS.
Resources	Processor Time	00:00:00.18
	Elapsed Time	00:00:00.00

Statistics

		not born in the Netherlands	one or two parents born outside NL
N	Valid	4104	4104
	Missing	0	0

Frequency Table

not born in the Netherlands

		Frequency	Percent	Valid Percent	Cumulative Percent
Valid	.00	3946	96.2	96.2	96.2
	1.00	158	3.8	3.8	100.0
	Total	4104	100.0	100.0	

one or two parents born outside NL

		Frequency	Percent	Valid Percent	Cumulative Percent
Valid	.00	3757	91.5	91.5	91.5
	1.00	347	8.5	8.5	100.0
	Total	4104	100.0	100.0	

```
FREQUENCIES VARIABLES=education_level_cat
 /ORDER=ANALYSIS.
```

Frequencies

Notes

Output Created		10-OCT-2023 15:28:...
Comments		
Input	Data	/Users/jeroenbruinsma/surfdrive - Bruinsma, Jeroen (GB)@surfdrive.surf.nl/analyze/11.CIBER.sav
	Active Dataset	DataSet1
	Filter	<none>
	Weight	<none>
	Split File	<none>
	N of Rows in Working Data File	4104
Missing Value Handling	Definition of Missing	User-defined missing values are treated as missing.
	Cases Used	Statistics are based on all cases with valid data.
Syntax		FREQUENCIES VARIABLES=education_level_cat /ORDER=ANALYSIS.
Resources	Processor Time	00:00:00.19
	Elapsed Time	00:00:00.00

Statistics

highest educational level catogory

N	Valid	4104
	Missing	0

highest educational level catogory

		Frequency	Percent	Valid Percent	Cumulative Percent
Valid	low	823	20.1	20.1	20.1
	middle	1545	37.6	37.6	57.7
	high	1736	42.3	42.3	100.0
	Total	4104	100.0	100.0	

```
FREQUENCIES VARIABLES=LIBRA_inactivity LIBRA_diet LIBRA_alc LIBRA_smoke LIB
RA_act
    LIBRA_cardiovascular LIBRA_kidney LIBRA_diabetes LIBRA_cholestorol LIBR
A_Obesity LIBRA_hypertension
    LIBRA_depression
  /ORDER=ANALYSIS.
```

Frequencies

Notes

Output Created		10-OCT-2023 15:29:...
Comments		
Input	Data	/Users/jeroenbruinsma/surfdrive - Bruinsma, Jeroen (GB)@surfdrive.surf.nl/analyze/11.CIBER.sav
	Active Dataset	DataSet1
	Filter	<none>
	Weight	<none>
	Split File	<none>
	N of Rows in Working Data File	4104
Missing Value Handling	Definition of Missing	User-defined missing values are treated as missing.
	Cases Used	Statistics are based on all cases with valid data.
Syntax		FREQUENCIES VARIABLES=LIBRA_inactivity LIBRA_diet LIBRA_alc LIBRA_smoke LIBRA_act LIBRA_cardiovascular LIBRA_kidney LIBRA_diabetes LIBRA_cholestorol LIBRA_Obesity LIBRA_hypertension LIBRA_depression /ORDER=ANALYSIS.

Notes

Resources	Processor Time	00:00:00.20
	Elapsed Time	00:00:01.00

Statistics

		PA norm = 150 min moderate PA per week (5 days) OR 60 min vigorous PA per week (3 days)	diet -1.7 for score 0	low alcohol consumption -1 for score 0 (<1 glass a day)	smoking +1.5 for score 1 (yes, I smoke)	social/cognitive activities -3.2 for score 0 (>median n activities per day)
N	Valid	4104	4104	4104	4104	4104
	Missing	0	0	0	0	0

Statistics

		cardiovascular_disease+1.0 for score 1 (yes)	kidney_disease +1.1 for score 1 (yes)	diabetes +1.3 for score 1 (yes)	high cholestorol+1.4 for score 1 (yes)	Obesity+1.6 for score 1 (yes)
N	Valid	4104	4104	4104	4104	4099
	Missing	0	0	0	0	5

Statistics

		high_blood_pressure+1.6 for score 1 (yes)	depression +2.1 for score 1 (yes)
N	Valid	4104	4104
	Missing	0	0

Frequency Table

PA norm = 150 min moderate PA per week (5 days) OR 60 min vigorous PA per week (3 days)

		Frequency	Percent	Valid Percent	Cumulative Percent
Valid	adherence to PA norm	1932	47.1	47.1	47.1
	non-adherence to PA norm	2172	52.9	52.9	100.0
	Total	4104	100.0	100.0	

diet -1.7 for score 0

		Frequency	Percent	Valid Percent	Cumulative Percent
Valid	-1.70	1867	45.5	45.5	45.5
	.00	2237	54.5	54.5	100.0
	Total	4104	100.0	100.0	

low alcohol consumption -1 for score 0 (<1 glass a day)

		Frequency	Percent	Valid Percent	Cumulative Percent
Valid	-1.00	3099	75.5	75.5	75.5
	.00	1005	24.5	24.5	100.0
	Total	4104	100.0	100.0	

smoking +1.5 for score 1 (yes, I smoke)

		Frequency	Percent	Valid Percent	Cumulative Percent
Valid	.00	3648	88.9	88.9	88.9
	1.50	456	11.1	11.1	100.0
	Total	4104	100.0	100.0	

social/cognitive activities -3.2 for score 0 (>median n activities per day)

		Frequency	Percent	Valid Percent	Cumulative Percent
Valid	-3.20	2047	49.9	49.9	49.9
	.00	2057	50.1	50.1	100.0
	Total	4104	100.0	100.0	

cardiovascular_disease+1.0 for score 1 (yes)

		Frequency	Percent	Valid Percent	Cumulative Percent
Valid	.00	3452	84.1	84.1	84.1
	1.00	652	15.9	15.9	100.0
	Total	4104	100.0	100.0	

kidney_disease +1.1 for score 1 (yes)

		Frequency	Percent	Valid Percent	Cumulative Percent
Valid	.00	4020	98.0	98.0	98.0
	1.10	84	2.0	2.0	100.0
	Total	4104	100.0	100.0	

diabetes +1.3 for score 1 (yes)

		Frequency	Percent	Valid Percent	Cumulative Percent
Valid	.00	3670	89.4	89.4	89.4
	1.30	434	10.6	10.6	100.0
	Total	4104	100.0	100.0	

high cholestorol+1.4 for score 1 (yes)

		Frequency	Percent	Valid Percent	Cumulative Percent
Valid	.00	2847	69.4	69.4	69.4
	1.40	1257	30.6	30.6	100.0
	Total	4104	100.0	100.0	

Obesity+1.6 for score 1 (yes)

		Frequency	Percent	Valid Percent	Cumulative Percent
Valid	.00	3181	77.5	77.6	77.6
	1.60	918	22.4	22.4	100.0
	Total	4099	99.9	100.0	
Missing	System	5	.1		
Total		4104	100.0		

high_blood_pressure+1.6 for score 1 (yes)

		Frequency	Percent	Valid Percent	Cumulative Percent
Valid	.00	2704	65.9	65.9	65.9
	1.60	1400	34.1	34.1	100.0
	Total	4104	100.0	100.0	

depression +2.1 for score 1 (yes)

		Frequency	Percent	Valid Percent	Cumulative Percent
Valid	.00	3370	82.1	82.1	82.1
	2.10	734	17.9	17.9	100.0
	Total	4104	100.0	100.0	

```
FREQUENCIES VARIABLES=LIBRA_tot
  /NTILES=4
  /STATISTICS=STDDEV MINIMUM MAXIMUM MEAN MEDIAN SKEWNESS SESKEW KURTOSIS S
EKURT
  /HISTOGRAM NORMAL
  /ORDER=ANALYSIS.
```

Frequencies

Notes

Output Created		10-OCT-2023 15:30:...
Comments		
Input	Data	/Users/jeroenbruinsma/surfdrive - Bruinsma, Jeroen (GB)@surfdrive.surf.nl/analyze/11.CIBER.sav
	Active Dataset	DataSet1
	Filter	<none>
	Weight	<none>
	Split File	<none>
	N of Rows in Working Data File	4104
Missing Value Handling	Definition of Missing	User-defined missing values are treated as missing.
	Cases Used	Statistics are based on all cases with valid data.
Syntax		FREQUENCIES VARIABLES=LIBRA_tot /NTILES=4 /STATISTICS=STDDEV MINIMUM MAXIMUM MEAN MEDIAN SKEWNESS SESKEW KURTOSIS SEKURT /HISTOGRAM NORMAL /ORDER=ANALYSIS.
Resources	Processor Time	00:00:02.60
	Elapsed Time	00:00:02.00

Statistics

LIBRA_tot

N	Valid	4104
	Missing	0
Mean		-.3488
Median		-.5000
Std. Deviation		2.89889
Skewness		.324
Std. Error of Skewness		.038
Kurtosis		-.161
Std. Error of Kurtosis		.076
Minimum		-5.90
Maximum		10.60
Percentiles	25	-2.6000
	50	-.5000
	75	1.6000

LIBRA_tot

		Frequency	Percent	Valid Percent	Cumulative Percent
Valid	-5.90	123	3.0	3.0	3.0
	-4.90	66	1.6	1.6	4.6
	-4.80	99	2.4	2.4	7.0
	-4.50	21	.5	.5	7.5
	-4.40	7	.2	.2	7.7
	-4.30	52	1.3	1.3	9.0
	-4.20	108	2.6	2.6	11.6
	-3.90	8	.2	.2	11.8
	-3.80	1	.0	.0	11.8
	-3.80	51	1.2	1.2	13.1
	-3.70	1	.0	.0	13.1
	-3.60	4	.1	.1	13.2
	-3.50	1	.0	.0	13.2
	-3.50	22	.5	.5	13.7
	-3.40	17	.4	.4	14.2
	-3.40	9	.2	.2	14.4
	-3.30	4	.1	.1	14.5
	-3.30	19	.5	.5	14.9
	-3.20	30	.7	.7	15.7
	-3.20	39	1.0	1.0	16.6
	-3.10	106	2.6	2.6	19.2
	-3.00	5	.1	.1	19.3
	-2.90	5	.1	.1	19.4

LIBRA_tot

	Frequency	Percent	Valid Percent	Cumulative Percent
-2.90	28	.7	.7	20.1
-2.80	33	.8	.8	20.9
-2.70	28	.7	.7	21.6
-2.70	138	3.4	3.4	25.0
-2.60	49	1.2	1.2	26.2
-2.50	1	.0	.0	26.2
-2.50	2	.0	.0	26.2
-2.40	2	.0	.0	26.3
-2.40	5	.1	.1	26.4
-2.40	8	.2	.2	26.6
-2.30	14	.3	.3	26.9
-2.20	18	.4	.4	27.4
-2.20	20	.5	.5	27.9
-2.10	3	.1	.1	27.9
-2.10	55	1.3	1.3	29.3
-1.90	10	.2	.2	29.5
-1.90	28	.7	.7	30.2
-1.90	1	.0	.0	30.2
-1.80	14	.3	.3	30.6
-1.80	12	.3	.3	30.9
-1.80	5	.1	.1	31.0
-1.70	5	.1	.1	31.1
-1.70	39	1.0	1.0	32.1
-1.70	51	1.2	1.2	33.3
-1.60	18	.4	.4	33.7
-1.60	140	3.4	3.4	37.2
-1.50	2	.0	.0	37.2
-1.50	1	.0	.0	37.2
-1.50	72	1.8	1.8	39.0
-1.40	4	.1	.1	39.1
-1.40	1	.0	.0	39.1
-1.30	9	.2	.2	39.3
-1.30	31	.8	.8	40.1
-1.30	4	.1	.1	40.2
-1.20	4	.1	.1	40.3
-1.20	40	1.0	1.0	41.3
-1.10	9	.2	.2	41.5
-1.10	46	1.1	1.1	42.6
-1.00	2	.0	.0	42.6
-1.00	157	3.8	3.8	46.5

LIBRA_tot

	Frequency	Percent	Valid Percent	Cumulative Percent
-.90	8	.2	.2	46.7
-.90	2	.0	.0	46.7
-.80	6	.1	.1	46.9
-.80	13	.3	.3	47.2
-.80	2	.0	.0	47.2
-.80	2	.0	.0	47.3
-.70	2	.0	.0	47.3
-.70	22	.5	.5	47.9
-.60	3	.1	.1	47.9
-.60	31	.8	.8	48.7
-.60	31	.8	.8	49.4
-.50	8	.2	.2	49.6
-.50	2	.0	.0	49.7
-.50	26	.6	.6	50.3
-.40	5	.1	.1	50.4
-.40	4	.1	.1	50.5
-.40	4	.1	.1	50.6
-.30	2	.0	.0	50.7
-.30	7	.2	.2	50.9
-.30	9	.2	.2	51.1
-.20	7	.2	.2	51.2
-.20	39	1.0	1.0	52.2
-.20	8	.2	.2	52.4
-.10	3	.1	.1	52.5
-.10	1	.0	.0	52.5
-.10	32	.8	.8	53.3
-.10	15	.4	.4	53.6
.00	1	.0	.0	53.7
.00	1	.0	.0	53.7
.00	3	.1	.1	53.8
.00	4	.1	.1	53.8
.00	103	2.5	2.5	56.4
.10	7	.2	.2	56.5
.10	181	4.4	4.4	60.9
.20	2	.0	.0	61.0
.20	14	.3	.3	61.3
.20	2	.0	.0	61.4
.30	2	.0	.0	61.4
.30	21	.5	.5	61.9
.30	5	.1	.1	62.1

LIBRA_tot

	Frequency	Percent	Valid Percent	Cumulative Percent
.40	3	.1	.1	62.1
.40	1	.0	.0	62.2
.40	33	.8	.8	63.0
.40	9	.2	.2	63.2
.50	5	.1	.1	63.3
.50	57	1.4	1.4	64.7
.60	2	.0	.0	64.7
.60	1	.0	.0	64.8
.60	69	1.7	1.7	66.4
.70	1	.0	.0	66.5
.70	6	.1	.1	66.6
.70	5	.1	.1	66.7
.80	4	.1	.1	66.8
.80	15	.4	.4	67.2
.80	6	.1	.1	67.3
.80	2	.0	.0	67.4
.90	35	.9	.9	68.3
.90	6	.1	.1	68.4
1.00	1	.0	.0	68.4
1.00	23	.6	.6	69.0
1.00	12	.3	.3	69.3
1.10	6	.1	.1	69.4
1.10	12	.3	.3	69.7
1.10	64	1.6	1.6	71.3
1.20	9	.2	.2	71.5
1.20	2	.0	.0	71.5
1.30	5	.1	.1	71.7
1.30	8	.2	.2	71.9
1.30	11	.3	.3	72.1
1.40	40	1.0	1.0	73.1
1.40	7	.2	.2	73.3
1.50	1	.0	.0	73.3
1.50	1	.0	.0	73.3
1.50	42	1.0	1.0	74.3
1.50	4	.1	.1	74.4
1.60	2	.0	.0	74.5
1.60	3	.1	.1	74.6
1.60	48	1.2	1.2	75.7
1.70	7	.2	.2	75.9
1.70	2	.0	.0	76.0

LIBRA_tot

	Frequency	Percent	Valid Percent	Cumulative Percent
1.70	71	1.7	1.7	77.7
1.80	7	.2	.2	77.9
1.80	4	.1	.1	77.9
1.90	1	.0	.0	78.0
1.90	1	.0	.0	78.0
1.90	26	.6	.6	78.6
1.90	6	.1	.1	78.8
2.00	1	.0	.0	78.8
2.00	31	.8	.8	79.6
2.10	2	.0	.0	79.6
2.10	5	.1	.1	79.7
2.10	26	.6	.6	80.4
2.20	1	.0	.0	80.4
2.20	42	1.0	1.0	81.4
2.30	13	.3	.3	81.7
2.30	1	.0	.0	81.7
2.40	12	.3	.3	82.0
2.40	13	.3	.3	82.4
2.50	1	.0	.0	82.4
2.50	40	1.0	1.0	83.4
2.60	2	.0	.0	83.4
2.60	1	.0	.0	83.4
2.60	23	.6	.6	84.0
2.60	3	.1	.1	84.1
2.70	36	.9	.9	84.9
2.80	12	.3	.3	85.2
2.80	2	.0	.0	85.3
2.90	2	.0	.0	85.3
2.90	13	.3	.3	85.6
2.90	10	.2	.2	85.9
3.00	44	1.1	1.1	87.0
3.10	7	.2	.2	87.1
3.10	36	.9	.9	88.0
3.10	2	.0	.0	88.1
3.20	1	.0	.0	88.1
3.20	32	.8	.8	88.9
3.30	10	.2	.2	89.1
3.30	24	.6	.6	89.7
3.40	6	.1	.1	89.8
3.50	17	.4	.4	90.3

LIBRA_tot

	Frequency	Percent	Valid Percent	Cumulative Percent
3.60	4	.1	.1	90.4
3.60	15	.4	.4	90.7
3.70	1	.0	.0	90.7
3.70	19	.5	.5	91.2
3.80	3	.1	.1	91.3
3.80	26	.6	.6	91.9
3.90	4	.1	.1	92.0
3.90	4	.1	.1	92.1
4.00	17	.4	.4	92.5
4.10	28	.7	.7	93.2
4.20	5	.1	.1	93.3
4.20	8	.2	.2	93.5
4.30	1	.0	.0	93.5
4.30	8	.2	.2	93.7
4.40	11	.3	.3	94.0
4.50	8	.2	.2	94.2
4.60	18	.4	.4	94.6
4.70	14	.3	.3	95.0
4.80	9	.2	.2	95.2
4.90	14	.3	.3	95.5
5.00	7	.2	.2	95.7
5.10	17	.4	.4	96.1
5.20	1	.0	.0	96.2
5.20	9	.2	.2	96.4
5.30	5	.1	.1	96.5
5.30	11	.3	.3	96.8
5.40	15	.4	.4	97.1
5.50	2	.0	.0	97.2
5.60	7	.2	.2	97.3
5.70	10	.2	.2	97.6
5.70	2	.0	.0	97.6
5.80	3	.1	.1	97.7
5.80	2	.0	.0	97.8
5.80	2	.0	.0	97.8
5.90	5	.1	.1	97.9
6.00	6	.1	.1	98.1
6.10	1	.0	.0	98.1
6.20	11	.3	.3	98.4
6.30	2	.0	.0	98.4
6.30	3	.1	.1	98.5

LIBRA_tot

	Frequency	Percent	Valid Percent	Cumulative Percent
6.40	5	.1	.1	98.6
6.50	3	.1	.1	98.7
6.60	6	.1	.1	98.8
6.70	7	.2	.2	99.0
6.80	1	.0	.0	99.0
6.80	2	.0	.0	99.1
6.90	4	.1	.1	99.2
7.00	6	.1	.1	99.3
7.10	1	.0	.0	99.3
7.20	1	.0	.0	99.4
7.30	1	.0	.0	99.4
7.40	1	.0	.0	99.4
7.50	3	.1	.1	99.5
7.60	2	.0	.0	99.5
7.80	1	.0	.0	99.6
7.90	1	.0	.0	99.6
7.90	1	.0	.0	99.6
8.00	3	.1	.1	99.7
8.10	2	.0	.0	99.7
8.50	1	.0	.0	99.8
8.70	1	.0	.0	99.8
8.80	1	.0	.0	99.8
9.10	2	.0	.0	99.9
9.50	1	.0	.0	99.9
9.60	2	.0	.0	99.9
10.20	2	.0	.0	100.0
10.60	1	.0	.0	100.0
Total	4104	100.0	100.0	

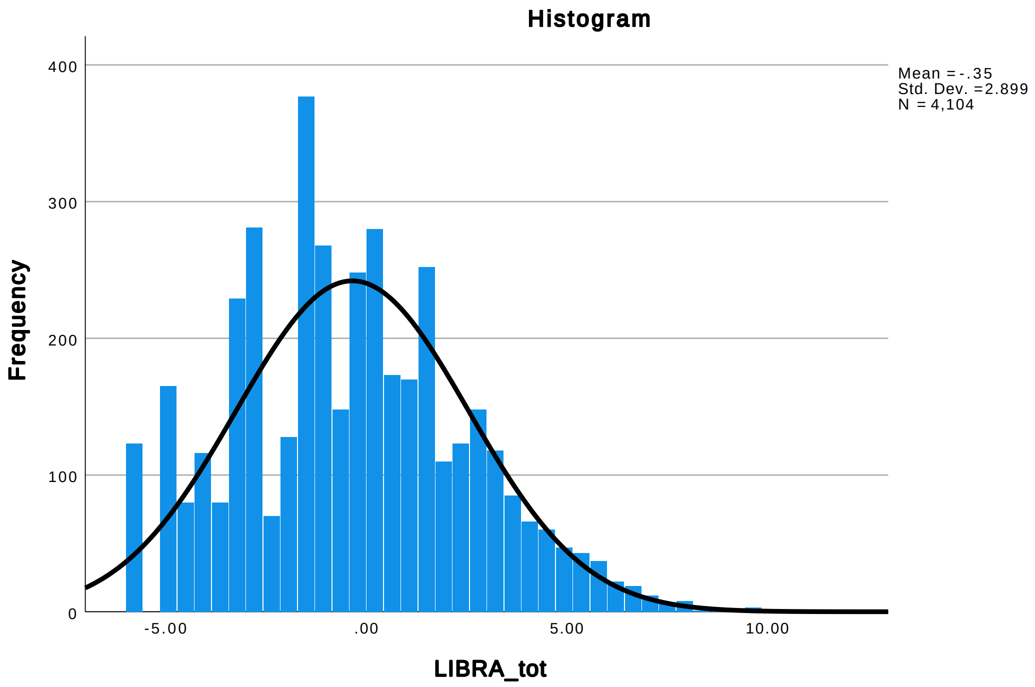
Histogram
Mean = -.35
Std. Dev. = 2.899
N = 4,104
400
300
200
100
0
Frequency
-5.00
.00
5.00
10.00
LIBRA_tot
