# Supplementary material for: Socio-Cognitive Determinants of Lifestyle Behavior in the Context of Dementia Risk Reduction: A Population-Based Study in the Netherlands
Source: J Alzheimers Dis. 2024 May 28;99(3):941–52. doi: 10.3233/JAD-231369 (PMC11191482; doi:10.3233/JAD-231369)
Supplement: Supplementary Material — 3. Section 3.2 Table 2 [file jad-99-jad231369-s009.pdf]

```
 ***************
Result paragraph 3.2 Misconceptions about adherence to recommendations and
intentions to change behavior
***************

***************
Translating needed items to English*
***************

VARIABLE LABELS PA_enough_T1 'Do you think you do enough exercise weekly?'.
ADD VALUE LABELS PA_enough_T1 1'yes' 2'somewhat' 3'no'.

VARIABLE LABELS Diet_Heatlhy 'Do you think you eat healthy?'.
ADD VALUE LABELS Diet_Heatlhy 1'yes' 2'somewhat' 3'no'.

VARIABLE LABELS Alc_Use_T1 'Do you think you overconsume alcohol?'.
ADD VALUE LABELS Alc_Use_T1 1'yes' 2'somewhat' 3'no'.

VARIABLE LABELS Cog_Heatlhy_T1 'Do you feel that you are socially and activ
ely engaged in life?'.
ADD VALUE LABELS Cog_Heatlhy_T1 1'yes' 2'somewhat' 3'no'.

***************
Crosstabs
***************
*Physical activity*

USE ALL.
COMPUTE filter_$=(Participated_T1 = 1).
VARIABLE LABELS filter_$ 'Participated_T1 = 1 (FILTER)'.
VALUE LABELS filter_$ 0 'Not Selected' 1 'Selected'.
FORMATS filter_$ (f1.0).
FILTER BY filter_$.
EXECUTE.

SORT CASES  BY inclusion_physical_activity_norm.
SPLIT FILE LAYERED BY inclusion_physical_activity_norm.

FREQUENCIES VARIABLES=PA_enough_T1
      /ORDER=ANALYSIS.
```

Frequencies

Notes

Output Created		11-OCT-2023 10:43:...
Comments		
Input	Data	/Users/jeroenbruinsma/surfdrive - Bruinsma, Jeroen (GB)@surfdrive.surf.nl/analyze/11.CIBER.sav
	Active Dataset	DataSet1
	Filter	Participated_T1 = 1 (FILTER)
	Weight	<none>
	Split File	0= adhere to norm, 1=do not adhere to norm
	N of Rows in Working Data File	3065
Missing Value Handling	Definition of Missing	User-defined missing values are treated as missing.
	Cases Used	Statistics are based on all cases with valid data.
Syntax		FREQUENCIES VARIABLES=PA_enough_T1 /ORDER=ANALYSIS.
Resources	Processor Time	00:00:00.18
	Elapsed Time	00:00:00.00

Statistics

Do you think you do enough exercise weekly?

.00	N	Valid	1287
		Missing	0
1.00	N	Valid	1778
		Missing	0

Do you think you do enough exercise weekly?

0= adhere to norm, 1=do not adhere to norm			Frequency	Percent	Valid Percent
.00	Valid	yes	985	76.5	76.5
		somewhat	256	19.9	19.9
		no	46	3.6	3.6
		Total	1287	100.0	100.0
1.00	Valid	yes	701	39.4	39.4
		somewhat	680	38.2	38.2
		no	397	22.3	22.3
		Total	1778	100.0	100.0

Do you think you do enough exercise weekly?

0= adhere to norm, 1=do not adhere to norm			Cumulative Percent
.00	Valid	yes	76.5
		somewhat	96.4
		no	100.0
		Total	
1.00	Valid	yes	39.4
		somewhat	77.7
		no	100.0
		Total	

```
 FILTER OFF.
USE ALL.
EXECUTE.

SPLIT FILE OFF.

*Diet*

USE ALL.
COMPUTE filter_$=(Participated_T1 = 1).
VARIABLE LABELS filter_$ 'Participated_T1 = 1 (FILTER)'.
VALUE LABELS filter_$ 0 'Not Selected' 1 'Selected'.
FORMATS filter_$ (f1.0).
FILTER BY filter_$.
EXECUTE.

SORT CASES  BY inclusion_MEDAS.
SPLIT FILE LAYERED BY  inclusion_MEDAS.

FREQUENCIES VARIABLES=Diet_Heatlhy
      /ORDER=ANALYSIS.
```

Frequencies

Notes

Output Created		11-OCT-2023 10:43:...
Comments		
Input	Data	/Users/jeroenbruinsma/surfdrive - Bruinsma, Jeroen (GB)@surfdrive.surf.nl/analyze/11.CIBER.sav
	Active Dataset	DataSet1
	Filter	Participated_T1 = 1 (FILTER)
	Weight	<none>
	Split File	0=>5, 1=5 (median) or lower
	N of Rows in Working Data File	3065
Missing Value Handling	Definition of Missing	User-defined missing values are treated as missing.
	Cases Used	Statistics are based on all cases with valid data.
Syntax		FREQUENCIES VARIABLES=Diet_Heatlhy /ORDER=ANALYSIS.
Resources	Processor Time	00:00:00.19
	Elapsed Time	00:00:00.00

Statistics

Do you think you eat healthy?

.00	N	Valid	1228
		Missing	0
1.00	N	Valid	1837
		Missing	0

Do you think you eat healthy?

0=>5, 1=5 (median) or lower			Frequency	Percent	Valid Percent	Cumulative Percent
.00	Valid	yes	943	76.8	76.8	76.8
		somewhat	273	22.2	22.2	99.0
		no	12	1.0	1.0	100.0
		Total	1228	100.0	100.0	
1.00	Valid	yes	1007	54.8	54.8	54.8
		somewhat	753	41.0	41.0	95.8
		no	77	4.2	4.2	100.0
		Total	1837	100.0	100.0	

```
 FILTER OFF.
USE ALL.
EXECUTE.

SPLIT FILE OFF.

*alc*

USE ALL.
COMPUTE filter_$=(Participated_T1 = 1).
VARIABLE LABELS filter_$ 'Participated_T1 = 1 (FILTER)'.
VALUE LABELS filter_$ 0 'Not Selected' 1 'Selected'.
FORMATS filter_$ (f1.0).
FILTER BY filter_$.
EXECUTE.

SORT CASES  BY inclusion_alcohol.
SPLIT FILE LAYERED BY inclusion_alcohol.

FREQUENCIES VARIABLES=Alc_Use_T1
```

Frequencies

Notes

Output Created		11-OCT-2023 10:43:...
Comments		
Input	Data	/Users/jeroenbruinsma/surfdrive - Bruinsma, Jeroen (GB)@surfdrive.surf.nl/analyze/11.CIBER.sav
	Active Dataset	DataSet1
	Filter	Participated_T1 = 1 (FILTER)
	Weight	<none>
	Split File	0=<1 glass a day, 1=1 or more glasses a day
	N of Rows in Working Data File	3065
Missing Value Handling	Definition of Missing	User-defined missing values are treated as missing.
	Cases Used	Statistics are based on all cases with valid data.
Syntax		FREQUENCIES VARIABLES=Alc_Use_T1 /ORDER=ANALYSIS.
Resources	Processor Time	00:00:00.18
	Elapsed Time	00:00:00.00

Statistics

Do you think you overconsume alcohol?

.00	N	Valid	1570
		Missing	655
1.00	N	Valid	840
		Missing	0

Do you think you overconsume alcohol?

0=<1 glass a day, 1= 1 or more glasses a day			Frequency	Percent	Valid Percent
.00	Valid	yes	4	.2	.3
		somewhat	122	5.5	7.8
		no	1444	64.9	92.0
		Total	1570	70.6	100.0
	Missing	System	655	29.4	
	Total		2225	100.0	
1.00	Valid	yes	93	11.1	11.1
		somewhat	384	45.7	45.7
		no	363	43.2	43.2
		Total	840	100.0	100.0

Do you think you overconsume alcohol?

0=<1 glass a day, 1= 1 or more glasses a day			Cumulative Percent
.00	Valid	yes	.3
		somewhat	8.0
		no	100.0
		Total	
	Missing	System	
	Total		
1.00	Valid	yes	11.1
		somewhat	56.8
		no	100.0
		Total	

```
 FILTER OFF.
USE ALL.
EXECUTE.

SPLIT FILE OFF.

*Social-cognitive activity (lowest percentile was used)*
```

```
FREQUENCIES VARIABLES=activities_per_week
  /NTILES=4
  /STATISTICS=MINIMUM MAXIMUM
  /ORDER=ANALYSIS.
```

Frequencies

Notes

Output Created		11-OCT-2023 10:43:...
Comments		
Input	Data	/Users/jeroenbruinsma/surfdrive - Bruinsma, Jeroen (GB)@surfdrive.surf.nl/analyze/11.CIBER.sav
	Active Dataset	DataSet1
	Filter	<none>
	Weight	<none>
	Split File	<none>
	N of Rows in Working Data File	4104
Missing Value Handling	Definition of Missing	User-defined missing values are treated as missing.
	Cases Used	Statistics are based on all cases with valid data.
Syntax		FREQUENCIES VARIABLES=activities_per_week /NTILES=4 /STATISTICS=MINIMUM MAXIMUM /ORDER=ANALYSIS.
Resources	Processor Time	00:00:00.28
	Elapsed Time	00:00:01.00

Statistics

activities_per_week

N	Valid	4104
	Missing	0
Minimum		.00
Maximum		63.54
Percentiles	25	11.5400
	50	17.6200
	75	24.0400

activities_per_week

		Frequency	Percent	Valid Percent	Cumulative Percent
Valid	.00	4	.1	.1	.1
	.08	1	.0	.0	.1
	.12	1	.0	.0	.1
	.20	2	.0	.0	.2
	.50	1	.0	.0	.2
	.54	3	.1	.1	.3
	.58	2	.0	.0	.3
	.62	1	.0	.0	.4
	.66	2	.0	.0	.4
	.74	1	.0	.0	.4
	1.00	1	.0	.0	.5
	1.04	2	.0	.0	.5
	1.08	4	.1	.1	.6
	1.12	1	.0	.0	.6
	1.16	1	.0	.0	.7
	1.50	5	.1	.1	.8
	1.54	2	.0	.0	.8
	1.58	1	.0	.0	.9
	1.62	5	.1	.1	1.0
	1.66	1	.0	.0	1.0
	1.66	1	.0	.0	1.0
	1.70	2	.0	.0	1.1
	1.74	1	.0	.0	1.1
	1.78	1	.0	.0	1.1
	1.82	1	.0	.0	1.1
	2.00	3	.1	.1	1.2
	2.04	1	.0	.0	1.2
	2.08	3	.1	.1	1.3
	2.12	2	.0	.0	1.4
	2.16	4	.1	.1	1.5
	2.20	6	.1	.1	1.6
	2.24	1	.0	.0	1.6
	2.28	1	.0	.0	1.7
	2.50	4	.1	.1	1.8
	2.54	3	.1	.1	1.8
	2.58	8	.2	.2	2.0
	2.62	4	.1	.1	2.1
	2.66	5	.1	.1	2.2
	2.70	1	.0	.0	2.3
	2.78	1	.0	.0	2.3

activities_per_week

	Frequency	Percent	Valid Percent	Cumulative Percent
3.00	5	.1	.1	2.4
3.04	5	.1	.1	2.5
3.08	4	.1	.1	2.6
3.12	2	.0	.0	2.7
3.16	5	.1	.1	2.8
3.20	1	.0	.0	2.8
3.24	4	.1	.1	2.9
3.50	3	.1	.1	3.0
3.54	6	.1	.1	3.1
3.58	3	.1	.1	3.2
3.62	3	.1	.1	3.3
3.66	5	.1	.1	3.4
3.70	2	.0	.0	3.5
3.74	2	.0	.0	3.5
3.78	1	.0	.0	3.5
4.00	6	.1	.1	3.7
4.04	5	.1	.1	3.8
4.08	7	.2	.2	4.0
4.12	10	.2	.2	4.2
4.16	8	.2	.2	4.4
4.20	3	.1	.1	4.5
4.24	3	.1	.1	4.6
4.28	1	.0	.0	4.6
4.50	5	.1	.1	4.7
4.54	2	.0	.0	4.8
4.58	8	.2	.2	4.9
4.62	6	.1	.1	5.1
4.66	7	.2	.2	5.3
4.70	4	.1	.1	5.4
4.74	2	.0	.0	5.4
5.00	3	.1	.1	5.5
5.04	6	.1	.1	5.6
5.08	7	.2	.2	5.8
5.12	10	.2	.2	6.0
5.16	8	.2	.2	6.2
5.20	6	.1	.1	6.4
5.24	2	.0	.0	6.4
5.28	1	.0	.0	6.5
5.50	8	.2	.2	6.7
5.54	5	.1	.1	6.8

activities_per_week

	Frequency	Percent	Valid Percent	Cumulative Percent
5.58	10	.2	.2	7.0
5.62	5	.1	.1	7.1
5.66	9	.2	.2	7.4
5.70	2	.0	.0	7.4
5.74	1	.0	.0	7.4
5.78	1	.0	.0	7.5
6.00	8	.2	.2	7.7
6.04	2	.0	.0	7.7
6.08	10	.2	.2	7.9
6.12	6	.1	.1	8.1
6.16	9	.2	.2	8.3
6.20	4	.1	.1	8.4
6.28	2	.0	.0	8.5
6.50	2	.0	.0	8.5
6.54	8	.2	.2	8.7
6.58	5	.1	.1	8.8
6.62	7	.2	.2	9.0
6.66	3	.1	.1	9.1
6.70	4	.1	.1	9.2
7.00	5	.1	.1	9.3
7.04	6	.1	.1	9.4
7.08	10	.2	.2	9.7
7.12	12	.3	.3	10.0
7.16	7	.2	.2	10.1
7.20	4	.1	.1	10.2
7.50	7	.2	.2	10.4
7.54	10	.2	.2	10.6
7.58	14	.3	.3	11.0
7.62	14	.3	.3	11.3
7.66	4	.1	.1	11.4
7.70	8	.2	.2	11.6
7.74	3	.1	.1	11.7
7.78	1	.0	.0	11.7
7.82	2	.0	.0	11.8
8.00	7	.2	.2	11.9
8.04	11	.3	.3	12.2
8.08	7	.2	.2	12.4
8.08	8	.2	.2	12.6
8.12	4	.1	.1	12.7
8.12	5	.1	.1	12.8

activities_per_week

	Frequency	Percent	Valid Percent	Cumulative Percent
8.12	8	.2	.2	13.0
8.16	1	.0	.0	13.0
8.16	2	.0	.0	13.1
8.16	8	.2	.2	13.3
8.20	1	.0	.0	13.3
8.20	5	.1	.1	13.4
8.24	1	.0	.0	13.4
8.24	3	.1	.1	13.5
8.24	1	.0	.0	13.5
8.28	1	.0	.0	13.5
8.28	1	.0	.0	13.6
8.50	10	.2	.2	13.8
8.54	12	.3	.3	14.1
8.58	8	.2	.2	14.3
8.58	6	.1	.1	14.4
8.62	3	.1	.1	14.5
8.62	1	.0	.0	14.5
8.62	5	.1	.1	14.7
8.66	6	.1	.1	14.8
8.66	2	.0	.0	14.9
8.66	2	.0	.0	14.9
8.70	4	.1	.1	15.0
8.70	1	.0	.0	15.0
8.70	5	.1	.1	15.2
8.74	4	.1	.1	15.3
8.78	1	.0	.0	15.3
9.00	12	.3	.3	15.6
9.04	12	.3	.3	15.9
9.08	7	.2	.2	16.0
9.08	4	.1	.1	16.1
9.12	8	.2	.2	16.3
9.12	3	.1	.1	16.4
9.12	1	.0	.0	16.4
9.16	2	.0	.0	16.5
9.16	2	.0	.0	16.5
9.16	4	.1	.1	16.6
9.20	3	.1	.1	16.7
9.20	2	.0	.0	16.7
9.20	3	.1	.1	16.8
9.24	4	.1	.1	16.9

activities_per_week

	Frequency	Percent	Valid Percent	Cumulative Percent
9.28	1	.0	.0	16.9
9.28	1	.0	.0	17.0
9.50	9	.2	.2	17.2
9.54	16	.4	.4	17.6
9.58	7	.2	.2	17.7
9.58	7	.2	.2	17.9
9.62	10	.2	.2	18.2
9.62	1	.0	.0	18.2
9.62	3	.1	.1	18.3
9.66	2	.0	.0	18.3
9.66	2	.0	.0	18.3
9.66	1	.0	.0	18.4
9.70	6	.1	.1	18.5
9.70	1	.0	.0	18.5
9.70	2	.0	.0	18.6
9.74	1	.0	.0	18.6
9.74	2	.0	.0	18.7
10.00	11	.3	.3	18.9
10.04	21	.5	.5	19.4
10.08	18	.4	.4	19.9
10.08	2	.0	.0	19.9
10.12	12	.3	.3	20.2
10.12	4	.1	.1	20.3
10.12	7	.2	.2	20.5
10.16	8	.2	.2	20.7
10.16	1	.0	.0	20.7
10.16	1	.0	.0	20.7
10.20	3	.1	.1	20.8
10.20	3	.1	.1	20.9
10.20	1	.0	.0	20.9
10.24	2	.0	.0	21.0
10.24	1	.0	.0	21.0
10.28	1	.0	.0	21.0
10.50	9	.2	.2	21.2
10.54	20	.5	.5	21.7
10.58	19	.5	.5	22.2
10.58	5	.1	.1	22.3
10.62	8	.2	.2	22.5
10.62	2	.0	.0	22.5
10.62	3	.1	.1	22.6

activities_per_week

	Frequency	Percent	Valid Percent	Cumulative Percent
10.66	6	.1	.1	22.8
10.66	2	.0	.0	22.8
10.66	1	.0	.0	22.8
10.70	4	.1	.1	22.9
10.70	3	.1	.1	23.0
10.70	1	.0	.0	23.0
10.74	1	.0	.0	23.1
11.00	10	.2	.2	23.3
11.04	11	.3	.3	23.6
11.08	6	.1	.1	23.7
11.08	5	.1	.1	23.8
11.12	10	.2	.2	24.1
11.12	1	.0	.0	24.1
11.12	4	.1	.1	24.2
11.16	8	.2	.2	24.4
11.16	2	.0	.0	24.4
11.16	2	.0	.0	24.5
11.20	4	.1	.1	24.6
11.20	1	.0	.0	24.6
11.24	3	.1	.1	24.7
11.50	10	.2	.2	24.9
11.54	19	.5	.5	25.4
11.58	20	.5	.5	25.9
11.58	4	.1	.1	26.0
11.62	12	.3	.3	26.3
11.62	4	.1	.1	26.4
11.62	1	.0	.0	26.4
11.66	7	.2	.2	26.6
11.66	1	.0	.0	26.6
11.66	3	.1	.1	26.7
11.70	6	.1	.1	26.8
11.74	2	.0	.0	26.9
11.74	1	.0	.0	26.9
11.74	1	.0	.0	26.9
11.82	1	.0	.0	26.9
12.00	11	.3	.3	27.2
12.04	16	.4	.4	27.6
12.08	19	.5	.5	28.0
12.08	2	.0	.0	28.1
12.12	13	.3	.3	28.4

activities_per_week

	Frequency	Percent	Valid Percent	Cumulative Percent
12.12	7	.2	.2	28.6
12.12	1	.0	.0	28.6
12.16	6	.1	.1	28.8
12.16	1	.0	.0	28.8
12.16	2	.0	.0	28.8
12.20	2	.0	.0	28.9
12.24	1	.0	.0	28.9
12.50	15	.4	.4	29.3
12.54	9	.2	.2	29.5
12.58	18	.4	.4	29.9
12.58	2	.0	.0	30.0
12.62	9	.2	.2	30.2
12.62	2	.0	.0	30.2
12.66	6	.1	.1	30.4
12.66	1	.0	.0	30.4
12.66	4	.1	.1	30.5
12.70	3	.1	.1	30.6
12.70	1	.0	.0	30.6
12.74	2	.0	.0	30.7
12.74	2	.0	.0	30.7
13.00	12	.3	.3	31.0
13.04	19	.5	.5	31.5
13.08	17	.4	.4	31.9
13.08	2	.0	.0	31.9
13.12	13	.3	.3	32.2
13.12	1	.0	.0	32.3
13.16	9	.2	.2	32.5
13.16	3	.1	.1	32.6
13.16	2	.0	.0	32.6
13.20	1	.0	.0	32.6
13.24	1	.0	.0	32.7
13.50	7	.2	.2	32.8
13.54	14	.3	.3	33.2
13.58	16	.4	.4	33.6
13.62	11	.3	.3	33.8
13.62	2	.0	.0	33.9
13.62	1	.0	.0	33.9
13.66	6	.1	.1	34.0
13.66	1	.0	.0	34.1
13.70	2	.0	.0	34.1

activities_per_week

	Frequency	Percent	Valid Percent	Cumulative Percent
13.70	1	.0	.0	34.1
13.70	1	.0	.0	34.2
13.74	1	.0	.0	34.2
14.00	14	.3	.3	34.5
14.04	17	.4	.4	34.9
14.08	19	.5	.5	35.4
14.08	2	.0	.0	35.5
14.12	9	.2	.2	35.7
14.12	2	.0	.0	35.7
14.16	5	.1	.1	35.8
14.16	2	.0	.0	35.9
14.16	1	.0	.0	35.9
14.20	4	.1	.1	36.0
14.20	2	.0	.0	36.1
14.24	1	.0	.0	36.1
14.24	1	.0	.0	36.1
14.24	1	.0	.0	36.1
14.28	1	.0	.0	36.2
14.28	1	.0	.0	36.2
14.50	14	.3	.3	36.5
14.54	22	.5	.5	37.1
14.58	15	.4	.4	37.4
14.58	2	.0	.0	37.5
14.62	11	.3	.3	37.7
14.62	4	.1	.1	37.8
14.62	3	.1	.1	37.9
14.66	6	.1	.1	38.1
14.66	2	.0	.0	38.1
14.70	4	.1	.1	38.2
14.70	1	.0	.0	38.2
14.74	1	.0	.0	38.3
14.78	1	.0	.0	38.3
15.00	11	.3	.3	38.5
15.04	16	.4	.4	38.9
15.08	15	.4	.4	39.3
15.08	1	.0	.0	39.3
15.12	21	.5	.5	39.8
15.12	1	.0	.0	39.9
15.16	5	.1	.1	40.0
15.16	1	.0	.0	40.0

activities_per_week

	Frequency	Percent	Valid Percent	Cumulative Percent
15.20	4	.1	.1	40.1
15.50	12	.3	.3	40.4
15.54	15	.4	.4	40.8
15.58	13	.3	.3	41.1
15.58	3	.1	.1	41.2
15.62	10	.2	.2	41.4
15.62	2	.0	.0	41.4
15.66	4	.1	.1	41.5
15.66	1	.0	.0	41.6
15.66	2	.0	.0	41.6
15.70	1	.0	.0	41.6
15.70	1	.0	.0	41.7
15.70	1	.0	.0	41.7
16.00	11	.3	.3	42.0
16.04	18	.4	.4	42.4
16.08	17	.4	.4	42.8
16.12	7	.2	.2	43.0
16.12	1	.0	.0	43.0
16.16	7	.2	.2	43.2
16.20	5	.1	.1	43.3
16.24	3	.1	.1	43.4
16.50	19	.5	.5	43.8
16.54	26	.6	.6	44.5
16.58	22	.5	.5	45.0
16.62	15	.4	.4	45.4
16.66	12	.3	.3	45.7
16.70	2	.0	.0	45.7
17.00	16	.4	.4	46.1
17.04	22	.5	.5	46.6
17.08	34	.8	.8	47.5
17.12	19	.5	.5	47.9
17.16	12	.3	.3	48.2
17.16	1	.0	.0	48.2
17.20	4	.1	.1	48.3
17.28	1	.0	.0	48.4
17.50	13	.3	.3	48.7
17.54	23	.6	.6	49.2
17.58	25	.6	.6	49.9
17.62	11	.3	.3	50.1
17.66	6	.1	.1	50.3

activities_per_week

	Frequency	Percent	Valid Percent	Cumulative Percent
17.66	2	.0	.0	50.3
17.70	4	.1	.1	50.4
18.00	24	.6	.6	51.0
18.04	24	.6	.6	51.6
18.08	16	.4	.4	52.0
18.12	22	.5	.5	52.5
18.12	1	.0	.0	52.5
18.16	10	.2	.2	52.8
18.16	2	.0	.0	52.8
18.20	7	.2	.2	53.0
18.24	3	.1	.1	53.1
18.24	2	.0	.0	53.1
18.28	1	.0	.0	53.1
18.50	16	.4	.4	53.5
18.54	28	.7	.7	54.2
18.58	15	.4	.4	54.6
18.62	11	.3	.3	54.8
18.66	10	.2	.2	55.1
18.66	1	.0	.0	55.1
18.70	1	.0	.0	55.1
19.00	18	.4	.4	55.6
19.04	28	.7	.7	56.3
19.08	20	.5	.5	56.7
19.12	16	.4	.4	57.1
19.16	2	.0	.0	57.2
19.20	5	.1	.1	57.3
19.24	2	.0	.0	57.4
19.50	11	.3	.3	57.6
19.54	20	.5	.5	58.1
19.58	18	.4	.4	58.6
19.62	10	.2	.2	58.8
19.62	1	.0	.0	58.8
19.66	9	.2	.2	59.0
19.70	1	.0	.0	59.1
20.00	23	.6	.6	59.6
20.04	23	.6	.6	60.2
20.08	21	.5	.5	60.7
20.12	12	.3	.3	61.0
20.16	7	.2	.2	61.2
20.24	1	.0	.0	61.2

activities_per_week

	Frequency	Percent	Valid Percent	Cumulative Percent
20.50	18	.4	.4	61.6
20.54	10	.2	.2	61.9
20.58	14	.3	.3	62.2
20.62	14	.3	.3	62.5
20.66	6	.1	.1	62.7
20.70	3	.1	.1	62.8
20.70	1	.0	.0	62.8
21.00	15	.4	.4	63.2
21.04	25	.6	.6	63.8
21.08	14	.3	.3	64.1
21.12	15	.4	.4	64.5
21.16	10	.2	.2	64.7
21.20	2	.0	.0	64.8
21.50	20	.5	.5	65.3
21.54	28	.7	.7	65.9
21.58	19	.5	.5	66.4
21.62	12	.3	.3	66.7
21.66	4	.1	.1	66.8
21.70	3	.1	.1	66.9
21.74	2	.0	.0	66.9
21.78	1	.0	.0	66.9
22.00	20	.5	.5	67.4
22.04	22	.5	.5	68.0
22.08	26	.6	.6	68.6
22.12	9	.2	.2	68.8
22.16	5	.1	.1	68.9
22.20	2	.0	.0	69.0
22.24	1	.0	.0	69.0
22.50	15	.4	.4	69.4
22.54	19	.5	.5	69.8
22.58	15	.4	.4	70.2
22.62	8	.2	.2	70.4
22.66	4	.1	.1	70.5
23.00	12	.3	.3	70.8
23.04	18	.4	.4	71.2
23.08	14	.3	.3	71.6
23.12	11	.3	.3	71.8
23.16	7	.2	.2	72.0
23.16	1	.0	.0	72.0
23.20	1	.0	.0	72.1

activities_per_week

	Frequency	Percent	Valid Percent	Cumulative Percent
23.24	1	.0	.0	72.1
23.50	25	.6	.6	72.7
23.54	27	.7	.7	73.3
23.58	13	.3	.3	73.7
23.62	7	.2	.2	73.8
23.62	1	.0	.0	73.9
23.66	1	.0	.0	73.9
23.70	2	.0	.0	73.9
24.00	27	.7	.7	74.6
24.04	18	.4	.4	75.0
24.08	22	.5	.5	75.6
24.12	13	.3	.3	75.9
24.16	3	.1	.1	76.0
24.20	2	.0	.0	76.0
24.50	18	.4	.4	76.4
24.54	17	.4	.4	76.9
24.58	12	.3	.3	77.1
24.62	9	.2	.2	77.4
24.66	3	.1	.1	77.4
25.00	24	.6	.6	78.0
25.04	17	.4	.4	78.4
25.08	17	.4	.4	78.8
25.12	8	.2	.2	79.0
25.16	1	.0	.0	79.1
25.24	1	.0	.0	79.1
25.50	11	.3	.3	79.4
25.54	20	.5	.5	79.8
25.58	14	.3	.3	80.2
25.62	5	.1	.1	80.3
25.66	1	.0	.0	80.3
25.66	1	.0	.0	80.4
25.70	3	.1	.1	80.4
26.00	15	.4	.4	80.8
26.04	18	.4	.4	81.2
26.08	14	.3	.3	81.6
26.12	6	.1	.1	81.7
26.16	1	.0	.0	81.7
26.20	1	.0	.0	81.8
26.50	10	.2	.2	82.0
26.54	15	.4	.4	82.4

activities_per_week

	Frequency	Percent	Valid Percent	Cumulative Percent
26.58	9	.2	.2	82.6
26.62	3	.1	.1	82.7
26.66	3	.1	.1	82.7
27.00	16	.4	.4	83.1
27.04	10	.2	.2	83.4
27.08	10	.2	.2	83.6
27.12	12	.3	.3	83.9
27.16	2	.0	.0	84.0
27.50	10	.2	.2	84.2
27.54	14	.3	.3	84.6
27.58	5	.1	.1	84.7
27.62	3	.1	.1	84.7
27.66	2	.0	.0	84.8
28.00	11	.3	.3	85.1
28.04	11	.3	.3	85.3
28.08	9	.2	.2	85.6
28.12	3	.1	.1	85.6
28.16	1	.0	.0	85.6
28.50	4	.1	.1	85.7
28.54	8	.2	.2	85.9
28.58	7	.2	.2	86.1
28.62	7	.2	.2	86.3
28.70	2	.0	.0	86.3
29.00	16	.4	.4	86.7
29.04	14	.3	.3	87.1
29.08	9	.2	.2	87.3
29.12	2	.0	.0	87.3
29.50	12	.3	.3	87.6
29.54	7	.2	.2	87.8
29.58	4	.1	.1	87.9
29.62	4	.1	.1	88.0
29.66	1	.0	.0	88.0
30.00	13	.3	.3	88.3
30.04	10	.2	.2	88.6
30.08	7	.2	.2	88.7
30.12	1	.0	.0	88.8
30.16	2	.0	.0	88.8
30.50	10	.2	.2	89.1
30.54	12	.3	.3	89.4
30.58	7	.2	.2	89.5

activities_per_week

	Frequency	Percent	Valid Percent	Cumulative Percent
30.62	5	.1	.1	89.6
30.70	1	.0	.0	89.7
31.00	9	.2	.2	89.9
31.04	14	.3	.3	90.2
31.08	7	.2	.2	90.4
31.16	1	.0	.0	90.4
31.20	1	.0	.0	90.4
31.50	5	.1	.1	90.6
31.54	9	.2	.2	90.8
31.58	4	.1	.1	90.9
31.62	1	.0	.0	90.9
31.70	1	.0	.0	90.9
32.00	12	.3	.3	91.2
32.04	8	.2	.2	91.4
32.08	4	.1	.1	91.5
32.16	1	.0	.0	91.5
32.50	8	.2	.2	91.7
32.54	15	.4	.4	92.1
32.58	4	.1	.1	92.2
32.62	2	.0	.0	92.3
33.00	13	.3	.3	92.6
33.04	7	.2	.2	92.7
33.08	6	.1	.1	92.9
33.50	9	.2	.2	93.1
33.54	9	.2	.2	93.3
33.58	1	.0	.0	93.3
33.62	2	.0	.0	93.4
34.00	8	.2	.2	93.6
34.04	3	.1	.1	93.7
34.08	4	.1	.1	93.8
34.12	1	.0	.0	93.8
34.50	14	.3	.3	94.1
34.54	3	.1	.1	94.2
34.58	1	.0	.0	94.2
34.62	1	.0	.0	94.2
35.00	9	.2	.2	94.5
35.04	7	.2	.2	94.6
35.08	6	.1	.1	94.8
35.12	2	.0	.0	94.8
35.50	8	.2	.2	95.0

activities_per_week

	Frequency	Percent	Valid Percent	Cumulative Percent
35.54	6	.1	.1	95.2
35.58	5	.1	.1	95.3
35.62	1	.0	.0	95.3
36.00	9	.2	.2	95.5
36.04	2	.0	.0	95.6
36.08	4	.1	.1	95.7
36.12	1	.0	.0	95.7
36.50	5	.1	.1	95.8
36.54	4	.1	.1	95.9
36.58	3	.1	.1	96.0
36.66	1	.0	.0	96.0
37.00	6	.1	.1	96.2
37.04	2	.0	.0	96.2
37.08	4	.1	.1	96.3
37.12	1	.0	.0	96.3
37.50	8	.2	.2	96.5
37.54	1	.0	.0	96.6
37.58	2	.0	.0	96.6
38.00	4	.1	.1	96.7
38.04	4	.1	.1	96.8
38.08	4	.1	.1	96.9
38.12	2	.0	.0	97.0
38.50	1	.0	.0	97.0
38.54	1	.0	.0	97.0
38.58	1	.0	.0	97.0
39.00	6	.1	.1	97.2
39.04	1	.0	.0	97.2
39.08	2	.0	.0	97.2
39.12	3	.1	.1	97.3
39.16	2	.0	.0	97.4
39.50	4	.1	.1	97.5
39.54	2	.0	.0	97.5
39.62	2	.0	.0	97.6
40.00	5	.1	.1	97.7
40.04	2	.0	.0	97.7
40.08	3	.1	.1	97.8
40.50	4	.1	.1	97.9
40.58	1	.0	.0	97.9
41.00	3	.1	.1	98.0
41.04	2	.0	.0	98.1

activities_per_week

	Frequency	Percent	Valid Percent	Cumulative Percent
41.12	2	.0	.0	98.1
41.16	1	.0	.0	98.1
41.50	2	.0	.0	98.2
41.54	1	.0	.0	98.2
41.58	1	.0	.0	98.2
42.00	1	.0	.0	98.2
42.04	4	.1	.1	98.3
42.08	1	.0	.0	98.4
42.50	3	.1	.1	98.4
42.58	4	.1	.1	98.5
42.62	2	.0	.0	98.6
43.00	1	.0	.0	98.6
43.04	2	.0	.0	98.7
43.08	1	.0	.0	98.7
43.50	5	.1	.1	98.8
44.00	4	.1	.1	98.9
44.08	1	.0	.0	98.9
45.00	3	.1	.1	99.0
45.04	1	.0	.0	99.0
45.50	1	.0	.0	99.0
45.54	1	.0	.0	99.1
45.58	2	.0	.0	99.1
46.00	2	.0	.0	99.2
46.50	1	.0	.0	99.2
46.54	1	.0	.0	99.2
47.00	4	.1	.1	99.3
47.04	2	.0	.0	99.4
47.58	2	.0	.0	99.4
48.00	2	.0	.0	99.5
48.54	1	.0	.0	99.5
50.50	2	.0	.0	99.5
51.00	2	.0	.0	99.6
51.04	1	.0	.0	99.6
51.54	1	.0	.0	99.6
52.54	1	.0	.0	99.7
53.00	2	.0	.0	99.7
53.04	1	.0	.0	99.7
54.00	1	.0	.0	99.8
54.04	1	.0	.0	99.8
55.00	1	.0	.0	99.8

activities_per_week

	Frequency	Percent	Valid Percent	Cumulative Percent
56.00	1	.0	.0	99.8
56.04	1	.0	.0	99.9
56.50	1	.0	.0	99.9
56.54	1	.0	.0	99.9
59.00	1	.0	.0	99.9
62.50	1	.0	.0	100.0
63.00	1	.0	.0	100.0
63.54	1	.0	.0	100.0
Total	4104	100.0	100.0	

```
RECODE activities_per_week(SYSMIS=SYSMIS) (Lowest thru 11.54=1) (ELSE=0) I
NTO
    activities_per_week_low
VARIABLE LABELS  activities_per_week_low'1 = lowest percentile (=<11.54)'.
EXECUTE.

USE ALL.
COMPUTE filter_$=(Participated_T1 = 1).
VARIABLE LABELS filter_$ 'Participated_T1 = 1 (FILTER)'.
VALUE LABELS filter_$ 0 'Not Selected' 1 'Selected'.
FORMATS filter_$ (f1.0).
FILTER BY filter_$.
EXECUTE.

SORT CASES  BY activities_per_week_low
SPLIT FILE LAYERED BY activities_per_week_low

FREQUENCIES VARIABLES=Cog_Heatlhy_T1
      /ORDER=ANALYSIS.
```

Frequencies

Notes

Output Created		11-OCT-2023 10:43:...
Comments		
Input	Data	/Users/jeroenbruinsma/surfdrive - Bruinsma, Jeroen (GB)@surfdrive.surf.nl/analyze/11.CIBER.sav
	Active Dataset	DataSet1
	Filter	Participated_T1 = 1 (FILTER)
	Weight	<none>
	Split File	1 = lowest percentile (=<11.54)
	N of Rows in Working Data File	3065
Missing Value Handling	Definition of Missing	User-defined missing values are treated as missing.
	Cases Used	Statistics are based on all cases with valid data.
Syntax		FREQUENCIES VARIABLES=Cog_Heatlhy_T1 /ORDER=ANALYSIS.
Resources	Processor Time	00:00:00.18
	Elapsed Time	00:00:00.00

Statistics

Do you feel that you are socially and actively engaged in life?

.00	N	Valid	2244
		Missing	0
1.00	N	Valid	821
		Missing	0

Do you feel that you are socially and actively engaged in life?

1 = lowest percentile (=<11.54)			Frequency	Percent	Valid Percent	Cumulative Percent
.00	Valid	yes	1506	67.1	67.1	67.1
		somewhat	601	26.8	26.8	93.9
		no	137	6.1	6.1	100.0
		Total	2244	100.0	100.0	
1.00	Valid	yes	423	51.5	51.5	51.5
		somewhat	321	39.1	39.1	90.6
		no	77	9.4	9.4	100.0
		Total	821	100.0	100.0	

```
 FILTER OFF.
USE ALL.
EXECUTE.

SPLIT FILE OFF.
```
